# On the Miocene *Cyprideis* species flock (Ostracoda; Crustacea) of Western Amazonia (Solimões Formation): Refining taxonomy on species level

**DOI:** 10.11646/zootaxa.3899.1.1

**Published:** 2014-12-18

**Authors:** Martin Gross, Maria Ines F. Ramos, Werner E. Piller

**Affiliations:** 1 Department for Geology and Palaeontology, Universalmuseum Joanneum, Weinzöttlstrasse 16, 8045 Graz, Austria. telephone: +43-316-8017-9733 martin.gross@museum-joanneum.at; 2 Coordenação de Ciências da Terra e Ecologia, Museu Paraense Emílio Goeldi, Avenida Perimetral, 1901, Terra Firme, Belém-PA 66077-830, Brazil mramos@museu-goeldi.br; 3 Institute of Earth Sciences, University of Graz, NAWI Graz, Heinrichstrasse 26, 8010 Graz, Austria werner.piller@uni-graz.at

**Keywords:** Brazil, fossil ostracods, Cytherideidae, taxonomy, morphology

## Abstract

The Miocene mega-wetland of western Amazonia holds a diverse, largely endemic ostracod fauna. Among them, especially the genus *Cyprideis* experienced a remarkable radiation. Micropalaeontologic investigations of a 400 m long sediment core (~62 km SW Benjamin Constant, Amazonia, Brazil) permitted a taxonomic revision of about two-thirds of hitherto described *Cyprideis* species. We evaluate the diagnostic value of shell characters and provide an extensive illustration of the intraspecific variability of species. Based on comparative morphology, the 20 recorded *Cyprideis* species are arranged in groups and subgroups. The “smooth” group comprises *C*. *amazonica*, *C*. *kotzianae*, *C*. *kroemmelbeini*, *C*. *machadoi*, *C*. *multiradiata*, *C*. *olivencai*, *C*. *paralela* and *C*. *simplex*; the “ornate” group *C*. *curucae* nom. nov., *C*. *cyrtoma*, *C*. aff. *graciosa*, *C*. *inversa*, *C*. *ituiae* **n. sp.,** *C*. *matorae* **n. sp.,** *C*. *minipunctata*, *C*. *munoztorresi* nom. nov., *C*. *pebasae*, *C*. *reticulopunctata*, *C*. *schedogymnos* and *C*. *sulcosigmoidalis*. Five species have been revalidated, two renamed, two synonymised and two are new descriptions. Along with 10 further formally established species, for which a review is pending, *Cyprideis* keeps at least 30 endemic species in that region during Miocene times. Up to 12 *Cyprideis* species have been found to occur sympatrically, representing >90 % of the entire ostracod fauna. Ostracod index species enable a biostratigraphic allocation of the well succession to the *Cyprideis minipunctata* to *Cyprideis cyrtoma* biozones, corresponding to a late Middle to early Late Miocene age (late Serravallian–early Tortonian).

## 1 Introduction

Species flocks (e.g. Greenwood 1984; Lecointre *et al.* 2013) as the result of accelerated divergence of closely related species within a certain ecosystem concern fundamental aspects of biologic evolution (i.e., modes, patterns and pace) and substantially affect past and current biodiversity (e.g. Glaubrecht & Köhler 2004; Schön & Martens 2004). Celebrated examples for such bursts of species originate from isolated islands and long-lived lakes as well, however, they are not restricted to them (e.g. Eastman & McCune 2000; Sullivan *et al.* 2002; Kocher 2004; Wilson *et al.* 2004; Wesselingh 2007; Grant & Grant 2008; Wilke *et al.* 2008; Köhler *et al.* 2010).

Among recent ostracods, the *Cyprideis* species flock of East African Lake Tanganyika forms an intensively studied example (e.g. Wouters & Martens 1992, 2001, 2008; Schön *et al.* 2000; Schön & Martens 2012). For the Neogene period, comparable radiations in fossil *Cyprideis* are well recognized from Lake Pannon (Central Europe; Kollmann 1960; Krstić 1968a, b; Van Harten 1990), the Palaeo-Mediterranean realm (Decima 1964; Bassiouni 1979; Ligios & Gliozzi 2012), and the Caribbean area (Van den Bold 1976) as well as from western Amazonia (Purper 1979; Whatley *et al.* 1998).

In Miocene times an enormous, predominately fluvio-lacustrine wetland (~1 million km^2^) shaped western Amazonia (Hoorn & Wesselingh 2010; Hoorn *et al.* 2010a; for diverging views see e.g. Westaway 2006; Latrubesse *et al.* 2010), which holds an amazingly diverse, largely endemic bivalve, gastropod and ostracod fauna. While detailed taxonomic evaluations (e.g. Nuttall 1990; Wesselingh 2006a) already provide a firm base for considerations on e.g. mollusc phylogeny and causes of speciation (Wesselingh 2006b, 2007; Anderson *et al.* 2010), ostracodologic research lags behind and still remains in the stage of alpha taxonomy.

Whereas first descriptions of fossil western Amazonian molluscs date well back to the 19^th^ century (Gabb 1869; see Wesselingh 2008 for a historical review), studies on ostracods started with the seminal monograph of Purper (1979; as conference proceedings: Purper 1977)—more than one century later. Subsequently, Sheppard & Bate (1980), Purper & Pinto (1983, 1985), Purper & Ornellas (1991) and Swain (1998) continued that work. Later, the comprehensive research of Muñoz-Torres *et al.* (1998, 2006) and Whatley *et al.* (1998, 2000) appreciably advanced ostracod taxonomy, leading to an ostracod-based biozonation as well as to initial phylogenetic hypotheses. It was up to these authors to formulate the western Amazonian *Cyprideis* species flock. Further publications come from Ramos (2006), Celestino & Ramos (2007), and Ramos *et al.* (2009). Wesselingh & Ramos (2010) reviewed the state of the art. Recent contributions are by Linhares *et al.* (2011), Gross *et al.* (2013) and Nascimento *et al.* (2013).

**Figure 1:**
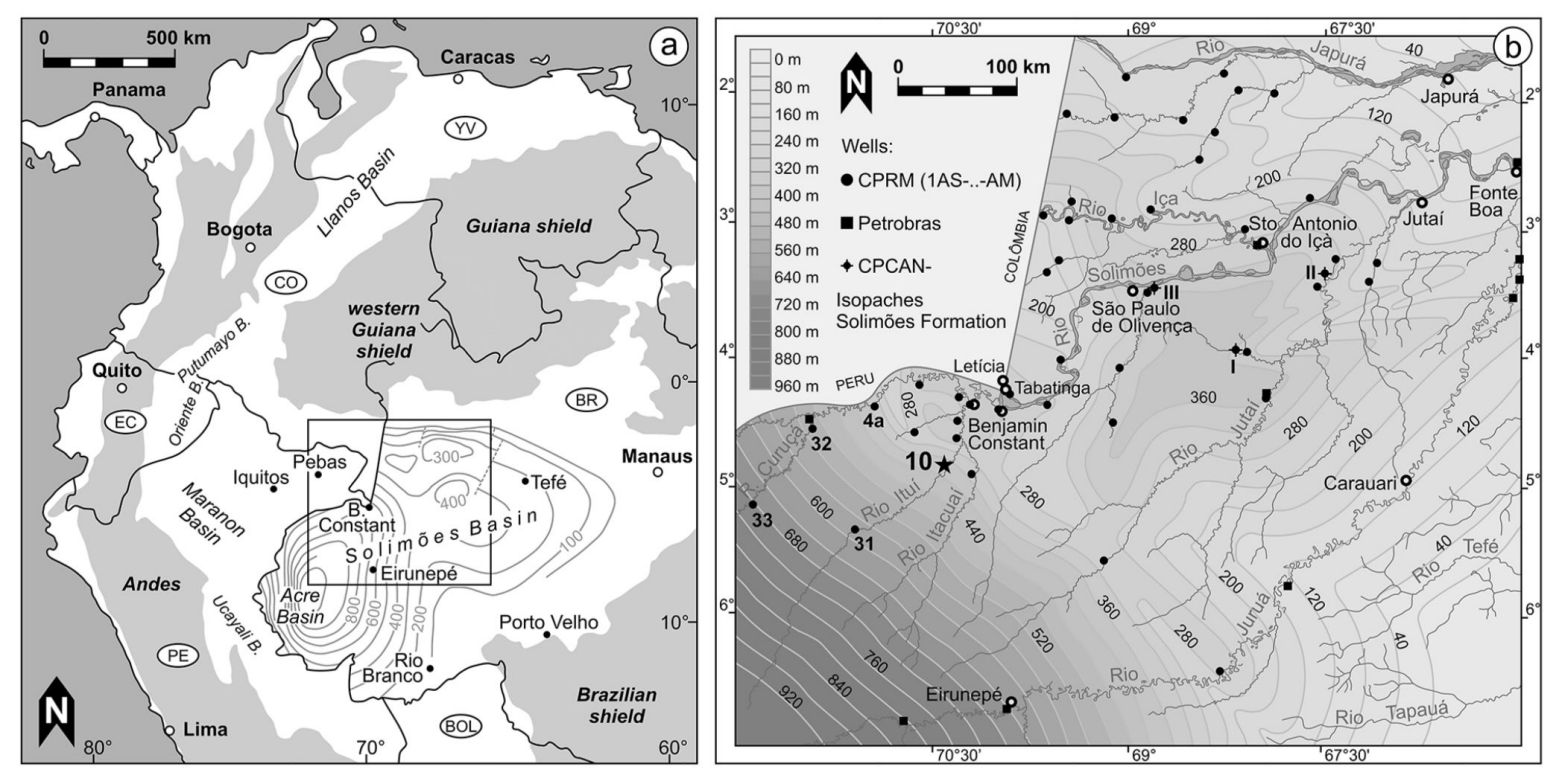
Location of the study area in western Amazonia. a: Isopatches [m] of the thickness of the Solimões Formation in the Acre and Solimões Basins (after Maia *et al.* 1977; Latrubesse *et al.* 2010); b: Coal-prospection area with isopatches [m] of the Solimões Fm. and position of exploration wells (after Maia *et al.* 1977; star = herein investigated core 1AS-10-AM; additional boreholes marked with numbers are mentioned in this study; location of CPCAN-I–III after Nuttall 1990).

The current paper investigates *Cyprideis* species from a ~400 m deep, continuously cored drill-hole from western Amazonia (Fig. 1). While the investigation of natural outcrops in this area (e.g. exposures along river banks) enables more detailed sedimentologic analyses (e.g. Hoorn 1994a, b; Gross *et al.* 2011), their stratigraphic range is usually limited, compared to such long drillings (Wesselingh *et al.* 2006a).

Based on our available material, about two-thirds of the western Amazonian *Cyprideis* species flock (~12 Ma) are revised, supplemented by an extensive documentation of the variability of species (including sexual and ontogenetic polymorphism as far as possible). We aim to improve and to facilitate reliable species identification, which forms the base for all further implications (phylogeny, biostratigraphy, palaeoecology, palaeogeography). To use the words of Wouters & Martens (2001: 111) in reference to Lake Tanganyika’s species flock: “Without such basic studies, […] advanced evolutionary and biodiversity research will remain difficult, if not impossible.”

## 2 Geological setting and short characterisation of CPRM well 1AS-10-AM

Our studied material originates from well 1AS-10-AM (Sucuriju, close to Rio Ituí; S 04°50’/W 70°22’; ~62 km SW Benjamin Constant; municipality Atalaia do Norte, state of Amazonia, Brazil; Figs. 1a–b). This core was drilled in the frame of a coal exploration campaign conducted by the Companhia de Pesquisa de Recursos Minerais (CPRM) in 1975 (Maia *et al.* 1977). Additional subsurface information was provided by earlier hydrocarbon (Petrobras) and lignite (Comissão do Plano do Carvão Nacional, CPCAN) prospection wells (Del’ Arco *et al.* 1977; Fernandes *et al.* 1977; Maia *et al.* 1977). Based on that, well 1AS-10-AM is located in the intracratonic Solimões Basin (e.g. Wanderley-Filho *et al.* 2010) and penetrates (except Holocene soils) sediments of the Solimões Formation. This formation comprises up to 1,000 m thick, largely pelitic–sandy alternations with intercalations of lignite as well as paleosols (e.g. Del’ Arco *et al.* 1977; Maia *et al.* 1977; Purper 1979; Hoorn 1994b; Latrubesse *et al.* 2010; Hoorn *et al.* 2010b; Silva-Caminha *et al.* 2010).

**Figure 2:**
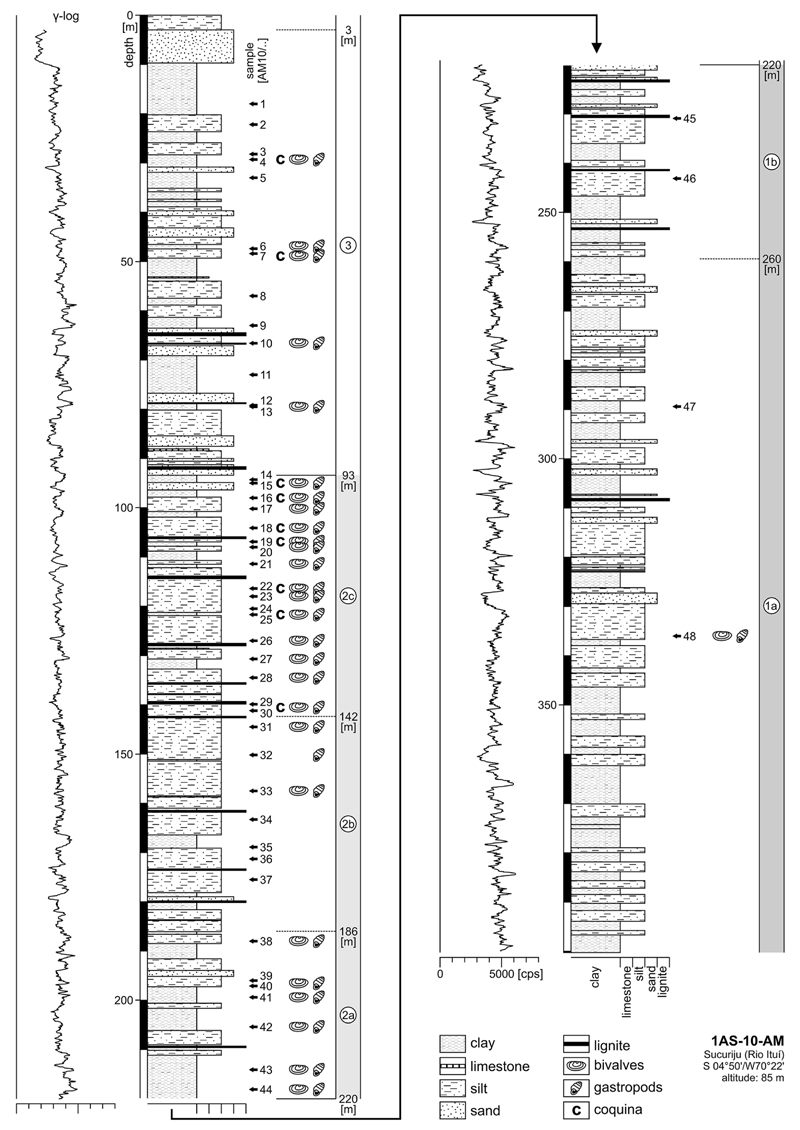
Section of well 1AS-10-AM (γ-log, lithology, samples, macrofossil content and core intervals; based on unpublished CPRM report and own observations).

Well 1AS-10-AM was continuously cored down to 400.25 m (Fig. 2). Its lithology consists of alternations of semi-indurated clay and silt. Up to metre-thick, sandy as well as decimetre-thick, lignite intercalations occur subordinately; centimetre-thick, clayey limestone layers may represent concretionary horizons. From the base to the top, sediments display a continuous coarsening-upward trend, which is equally expressed by the gamma ray-log. Based on the lithology and macrofossil content, the section is divided into three intervals (indication of colours follows the unpublished CPRM report):

**Interval 1** (~400–220 m). Up to ~260 m (interval 1a), the section is dominated by greenish-grey, reddish or yellowish mottled pelites (at least in part paleosols) with few sandy intercalations and one lignite layer (~308 m). Between ~260–220 m (interval 1b), lignite layers are more frequent. Only at ~336 m (sample AM10/48), a centimetre-thick layer with few mollusc remains is observed in interval 1.

**Interval 2** (~220–93 m). Above ~220 m, mollusc shells (bivalves, gastropods) become a common lithologic component. From ~220–186 m (interval 2a) greenish-grey, partly silty clay dominates, which contains some mollusc remains. Mainly grey, clayey silts follow up-section (~186–142 m, interval 2b), yielding only sporadically macrofossils (especially at the transition to interval 2c). Interval 2c (~142–93 m) is composed of grey silt with some clay interlayers and several lignite beds. Bivalve and gastropod remains are common, forming in part coquinas.

**Interval 3** (~93–0 m). At interval 3, sandy layers become more frequent, the fossil content decreases (except at samples AM10/7 and 4) and the last lignites are recorded at ~66 m. Brownish and lateritic pelites at the top (3–0 m) represent Holocene soil formation (unpublished CPRM report).

## 3 Material and methods

Core material as well as supplementary data are available at the Departamento Nacional da Produção Mineral (DNPM) in Manaus. For micropalaeontologic investigations 250 g of dried sediment (40° C, 24 h) were washed through standard sieves (63/125/250/500 µm) by using diluted hydrogen peroxide for disintegration (H_2_O_2_ : H_2_O = 1 : 5). Wet sieve residuals were washed with ethanol (70 %) before drying (40° C, 24 h). Residuals ≥250 μm were picked out completely for their biogenic content. The current study considers only specimens of the ostracod genus *Cyprideis* acquired from that sieve-fraction (ESM 1). From the ≥125 μm sieve residuals, 0.2 g/sample were picked, containing largely juvenile and/or fragmented ostracod valves (mainly *Cyprideis*). Specimens of ostracod taxa with minute body sizes (e.g. *Skopaeocythere*) as well as foraminifers are restricted to that fraction but will be the subject of further research.

Prior to scanning electron microscope photography (JEOL, JSM-6610LV), selected specimens were photographed in transmitted light (Leica M205C, camera: DFC290) and measured (Leica Application Suite V3.6.0; ESM 2). Focus stacked images were obtained by combining ~20 transmitted light photographs per specimen (external view; covered with distilled water) with the software Helicon Focus 5.3. For basic statistics, the software package PAST 1.97 (Hammer & Harper 2008) was used.

## 4 Systematic palaeontology

Suprageneric classification follows Meisch (2000) and Martens & Savatenalinton (2011). General descriptions of known species are provided by Purper (1979), Sheppard & Bate (1980), Muñoz-Torres *et al.* (1998), Whatley *et al.* (1998), and Ramos (2006). Further characteristics or deviations are discussed in the remarks. Type material as well as figured specimens are housed in the collection of the Museu Paraense Emílio Goeldi, Belém (Inv. No. MPEG-210-M to MPEG-495-M), additional material is stored at the Universalmuseum Joanneum, Department for Geology & Palaeontology, Graz (Inv. No. UMJG&P 211.038).

Abbreviations: L = left valve, R = right valve; ♀ = female, ♂ = male, A-1, … = juvenile stages; l = length, h = height (both in millimetres; length of spines not included in measurements), n = number of measured specimens.

**Class Ostracoda Latreille, 1802**

**Order Podocopida Sars, 1866**

**Superfamily Cytheroidea Baird, 1850**

**Family Cytherideidae Sars, 1925**

**Subfamily Cytherideinae Sars, 1925**

**Genus *Cyprideis* Jones, 1857**

Type species: *Candona torosa* Jones, 1850

### 4.1 Remarks to supraspecific taxonomy

Whatley *et al.* (1998) emended the diagnosis of *Cyprideis* and placed several genera into its synonymy. Among them are the following taxa, founded on fossil Amazonian species: *Amazonacytheridea* Purper, 1979, *Botulocyprideis* Sheppard & Bate, 1980 (emended by Purper & Pinto, 1983), *Chlamydocytheridea* Purper, 1979, *Nealecythere* Purper & Pinto, 1983, *Paulacoutoia* Purper, 1979, *Pseudoparakrithella* Purper, 1979 and *Sohnicythere* Purper & Pinto, 1983. In addition, several Amazonian species, originally assigned to *Cytheridea* Bosquet, 1852, were transferred to *Cyprideis* by these authors (see also Muñoz-Torres *et al.* 1998). In particular, the smooth, vestibulate species—formerly attributed to *Amazonacytheridea*, *Chlamydocytheridea*, *Paulacoutoia* and *Pseudoparakrithella* (with the possible synonyms *Botulocyprideis* and *Nealecythere*)—diverge significantly from *Cyprideis* as defined by earlier authors (e.g. Kollmann 1960; Benson *et al.* 1961; Van Morkhoven 1963; Sandberg 1964a). Since a discussion of this supraspecific concept is beyond the scope of the present investigation and subject of upcoming works, we apply it here with some reservation.

### 4.2 Conceptual notes to species identification herein

As usual, fossil species are based exclusively on morphologic characters, in this case on all features observable on the ostracods’ valves. Typically, a combination of traits is used for species definition (multidimensional species definition; Sbordoni 1993).

We are aware that specimens obtained from our samples are time-averaged to some degree and, e.g. seasonal or interannual variations (abundance, size, etc.) are blurred (e.g. Heip 1976; Schweitzer & Lohmann 1990). While disadvantageous on one side (e.g. obscuration of evolutionary patterns; increase of phenotypic variance of species), such fossil populations comprise an array of biologic generations and “random”, short-term variations are advantageously suppressed (Neil 2000; Bush *et al.* 2002; Hunt 2004, 2010; Hohenegger 2014).

In the present case, we usually found various ontogenetic stages of taxa within the samples, which signalise insignificant relocation or alteration of the assemblages. Therefore, we assume the ostracod faunas of each sample to represent a fossil biocoenosis (autochthonous thanatocoenosis; Whatley 1988; Boomer *et al.* 2003).

Recent *Cyprideis* reproduce sexually and—as ostracods in general—moult during their ontogeny. When females, males and as far as possible juvenile valves can be related to one morphospecies, we consider them as genetically isolated, sympatric population/species with reference to other morphotypes co-occurring in the same sample (best documented with both sexes and juveniles alike; Schweitzer & Lohmann 1990).

Shell characters, which show gradual transitions within one sample and/or vary (“randomly”) between samples (in this case in time), while the cluster of other features remains constant, are regarded as intraspecific variability. Some valve characters of modern *Cyprideis* species (best studied in *Cyprideis torosa* (Jones, 1850)) display substantial, environmentally cued variability, which complicates the recognition of phenotypic or species diagnostic traits (Gross *et al.* 2008; Ligios & Gliozzi 2012; for soft parts compare Wouters 2002, 2003):

**Shape.** The general shape (ovate, subrectangular, subtrapezoidal, etc.) of valves (especially of their outline in lateral view) is an essential character for (*Cyprideis*) species discrimination (among many others: Sandberg 1964a). This has been demonstrated successfully with geometric morphometric methods equally. Nonetheless, if outlines are very similar also quantitative methods fail and other traits must be considered (Gross *et al.* 2008; Ligios & Gliozzi 2012). Intraspecific variations of the outline—apart from sexual and ontogenetic polymorphism—largely concern the development of the females’ brood pouch (Kollmann 1960; Sandberg 1964a). For extant *C*. *torosa*, Carbonnel (1983) reported minor variations in shape correlated with different salinities, which, however, could not be confirmed by Frenzel *et al.* (2011).

**Size** *C*. *torosa* varies significantly in size. This has been found to be related to salinity (e.g. Van Harten 1975, 1996) or not (Kilenyi 1971; Vesper 1972a; Frenzel 1991) or discussed to be linked (additionally) to other parameters like organic contents (food supply; Vesper 1972a; Van Harten 1975). Lately, Boomer & Frenzel (2011) reported that *C*. *torosa* is larger below 8–9 psu and smaller above this, probably physiologically controlled threshold (Aladin 1993). However, the relative size of *Cyprideis* valves can be a valuable diagnostic feature, especially in cases where more than one species co-occur within one sample (e.g. Sandberg 1964a; Keen 1982; Gross *et al.* 2008; Ligios & Gliozzi 2012). Conversely, absolute dimensions of *Cyprideis* species—like recent *C*. *torosa*—fluctuate noticeably between localities (spatial) and as shown in the current study between samples (strata/temporal). Thus, absolute dimensions of species may significantly overlap and are a useful discriminating character on population level only (within one locality or one palaeontologic sample respectively).

**Ornament.** The ornament of *Cyprideis* is subject to serious intraspecific variation, ranging from smooth, punctated to reticulated surfaces. Parameters like calcium carbonate content (Van den Bold 1976), salinity (Sandberg 1964b) or the Mg/Ca ratio of the water (Carbonel 1982)—indirectly related to salinity—are under discussion to cause this variability. Compared to modern *Cyprideis*, we observed pronounced variations in ornamentation, which should not be over-interpreted (Sandberg 1964a, b). However, by examining a large number of valves, some stable ornamentation patterns can be recognized within species (e.g. for the current work about one thousand SEM-photographs of *Cyprideis* were taken, supplemented by the same amount of light microscopic pictures). Hence, under the assumption that variability is adequately covered, basic traits of the ornament (including the sulcus) can thoroughly form a diagnostic feature (note that keys to recent *Cyprideis* species likewise use the valves’ ornament as a diagnostic character; e.g. Smith & Delorme 2010; Karanovic 2012).

**Nodes.** Frequently, nodes (hollow protuberances) are developed on *Cyprideis*’ surface, which gave rise to long lasting debates about their taxonomic value (for reviews see e.g. Sandberg 1964a; Vesper 1972b, 1975; Van Harten 2000). Lately, the phenomenon of nodosity has been explained by Keyser & Aladin (2004) and Keyser (2005) to be a pathologic effect caused by osmoregulatory problems of the animals during moulting. However, the abundance of noded valves can be a useful proxy for salinity (noded valves dominate at a salinity below 7–8 psu) and/or Ca^2+^ contents of the water (a low amount of calcium leads to increased nodosity; Frenzel *et al.* 2012). Interestingly, not a single noded *Cyprideis* valve was found among the current material (~12,000 valves; ESM 1).

**Normal pores.** All *Cyprideis* species of the present investigation bear roundish normal pores of sieve type (a depressed sieve plate with a small, eccentric pore). The position and number of normal pores in *C*. *torosa* is rather stable and supposed to be an additional, genetically fixed, diagnostic character (Rosenfeld 1982). On the other hand, their shape varies along the salinity gradient (rounded shapes predominate in low saline settings), which is valuable for palaeosalinity calculations (e.g. Rosenfeld & Vesper 1976; Medley *et al.* 2008; Pint *et al.* 2012). No obvious differences in shape have been observed within the current specimens. Sometimes their investigation is hampered due to adhering sediment. For these reasons, the analysis of normal sieve pore pattern (shape, position, size, number) and their possible taxonomic as well as palaeoecological relevance is not considered in the present study.

**Marginal denticulations and spines.** Conversely to the assumption of Wouters (2002; for *C*. *torosa*), the basic pattern of anteromarginal and posteroventral spines/denticles (e.g. presence/absence, position, shape) is found several times to be an effective diagnostic character in *Cyprideis* (e.g. Kollmann 1960; Sandberg 1964a; Schweitzer & Lohmann 1990; Gross *et al.* 2008; Ligios & Gliozzi 2012; for recent *Cyprideis* see e.g. Smith & Delorme 2010; Karanovic 2012). Nevertheless, variations (e.g. strength, number) occur, which need to be as carefully examined as the preservation of valves. Marginal spines can be a useful character for species delineation, but should not be used as a single diagnostic trait and in a too tight manner (e.g. relying on the exact number of spines). The fundamental pattern of marginal spines is already developed in juvenile individuals, but the number of spines is frequently reduced in the adult stage (Sandberg 1964a; Gross *et al.* 2013; and present investigation).

**Inner lamella.** Intraspecific variability of the inner lamella (e.g. width, course, position of the selvage) as well as of marginal pore canals (shape, course, number) has been barely studied systematically in recent *Cyprideis*. Sandberg (1964a) noted only slight interspecific differences in the width of the anterior inner lamella (for a general statement see Van Morkhoven 1962). However, for some species with significantly narrower or wider inner lamella it can be an (additionally) characterising feature. For marginal pore canals Sandberg (1964a) observed minor differences in shape (note: his genus concept does not include the presence of a vestibulum) but mentioned that the density of (anterior) marginal pores can be a species diagnostic trait. In view of the Amazonian species and genera included by Whatley *et al.* (1998) in *Cyprideis*, striking interspecific differences in the inner lamella and (obviously linked with that) in marginal pore canal patterns are evident, which provide a useful diagnostic trait. Subordinate intraspecific variations of the vestibulum are observed between left and right valves as well as between both sexes (Whatley *et al.* 1998; and present work).

**Hinge.** Concerning recent *Cyprideis* little is known about the intraspecific variability of its hinge. Only Sandberg (1964a) noticed some variations in the crenulation of the (postero-)median element. Although the general pattern of the hinge (e.g. proportions of hinge elements, expression of teeth or denticles) is mainly of supraspecific significance (e.g. Kollmann 1960; Wouters & Martens 1992; Tibert *et al.* 2003), in combination with additional characters it can be of species diagnostic value (e.g. Gross *et al.* 2008; Ligios & Gliozzi 2012). Occasionally, within fossil *Cyprideis* material reversed valve overlap and inverse hinges respectively are documented (Krstić 1968a, b; Van den Bold 1976; Purper & Pinto 1983, 1985; Whatley *et al.* 1998; Gross *et al.* 2013; and herein). The taxonomic or environmental importance of this feature remains unclear (Kollmann 1960; Krstić 1968a, b, 1971; Van den Bold 1976; Purper & Pinto 1985; Whatley *et al.* 1998).

**Central muscle scars.** Variability is low in *Cyprideis’* central muscle scar pattern. Only the shape of the upper mandibular scar seems to be useful to some degree for species discrimination (Sandberg 1964a; Schweitzer & Lohmann 1990). No substantial intra- and interspecific differences in central muscle scar arrangement or shape were observed in our material.

Bringing the discussion above to the point: more than a single character is necessary for proper *Cyprideis* species definitions and species must be defined with a cluster of traits (“Gesamthabitus”; Kollmann 1960: 139).

### 4.3 Western Amazonian *Cyprideis* species groupings

**Figure 3:**
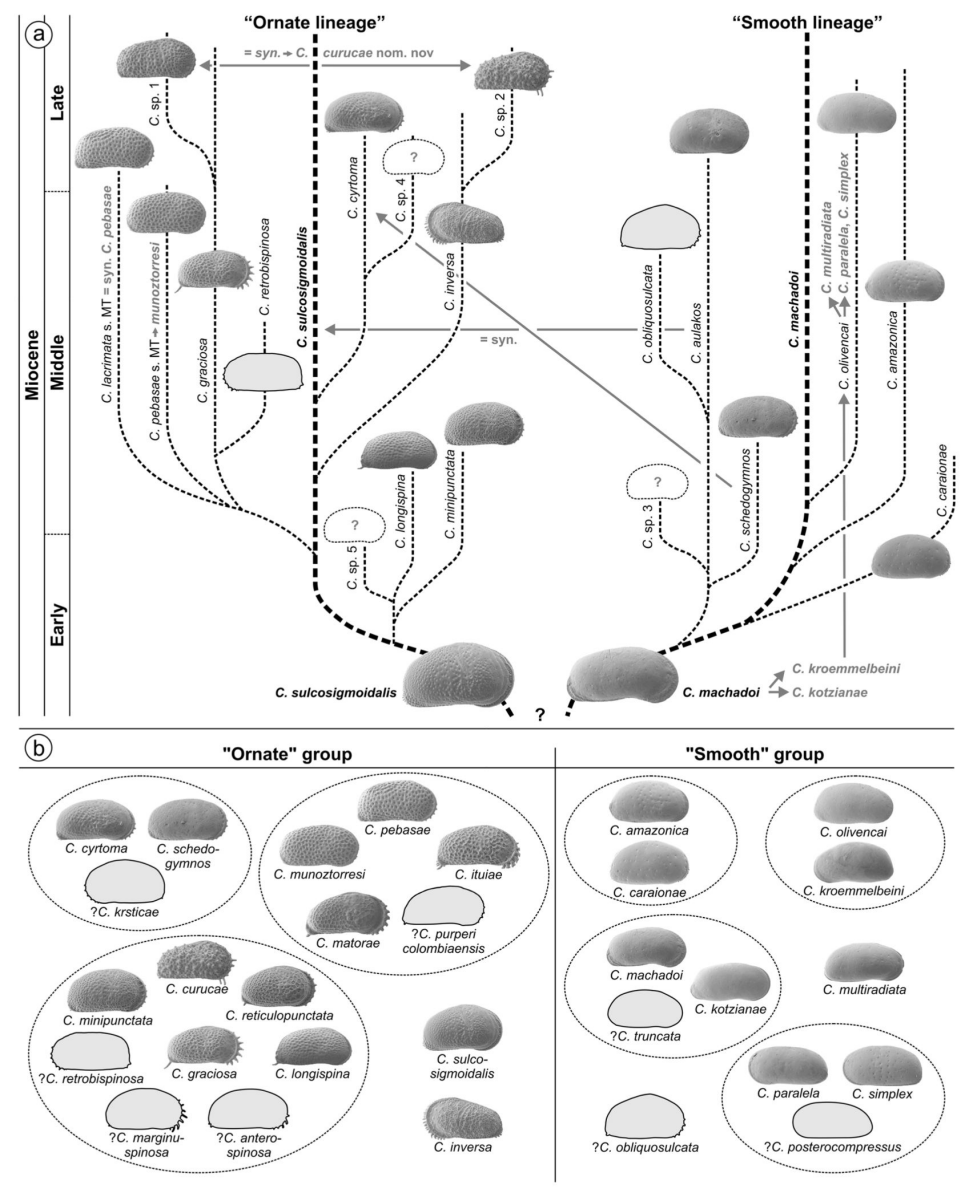
Western Amazonian *Cyprideis* species groups. a: Phylogenetic model of Muñoz-Torres *et al.* (2006; Whatley *et al.* 1998); chronology and phylogenetic relations (stippled lines) redrawn form Muñoz-Torres *et al.* (2006); taxonomic rearrangements in group allocations in grey (*C* = *Cyprideis*, s. MT = sensu Muñoz-Torres *et al.* (1998), syn. = synonymous); b: herein proposed grouping, based exclusively on morphologic similarities (species without photographs = revision pending).

Whatley *et al.* (1998) and Muñoz-Torres *et al.* (2006) proposed a phylogenetic scheme for endemic western Amazonian *Cyprideis* based on stratigraphic occurrence as well as on comparative morphology (Fig. 3a). These authors introduced two main lineages: the “ornate” and the “smooth” lineage with *C*. *sulcosigmoidalis* and *C*. *machadoi* as “core” species, respectively. Both are characterised by their surface ornament (ornamented vs. smooth). In addition, members of the “smooth” lineage generally have a wide inner lamella, principally bi- or polyfurcating (anterior) marginal pore canals and all develop a vestibulum. In “ornate” species, the inner lamella is comparably narrow; they lack polyfurcated radial pore canals as well as a vestibulum.

Our results confirm this generally applied grouping, here used for the basic arrangement of taxa (see chapter 4.5. and 4.6.), instead of a strictly alphabetic order, which would hamper a comparison between similar species. However, based on the current investigation some regroupings were necessary as well as the addition of some characters to refine both groups. The traits “size” (in both groups from small to large), “normal pores” (in all species of sieve type and roundish) and “central muscle scar pattern” (in all species generotypic; also the upper mandibular scar throughout round to elongate-oval) have no significance for this grouping.

#### 4.3.1 Nominal species and emended characteristics of the “smooth” *Cyprideis* group

**Species** (generic assignment sensu Whatley *et al.* 1998; alphabetic order; *not among the current material): *Cyprideis amazonica* Purper, 1979; *Cyprideis caraionae Purper & Pinto, 1985; *Cyprideis kotzianae* (Purper & Ornellas, 1991); *Cyprideis kroemmelbeini* (Purper, 1979); *Cyprideis machadoi* (Purper, 1979) [syn. *Otarocyprideis elegans* Sheppard & Bate, 1980; ?syn. *Cyprideis truncata Purper, 1979; re-examination pending]; *Cyprideis multiradiata* (Purper, 1979); *Cyprideis olivencai* (Purper, 1979); *Cyprideis paralela* (Purper, 1979) [?syn. *Cyprideis posterocompressus (Purper & Pinto, 1983); re-examination pending]; *Cyprideis simplex* (Sheppard & Bate, 1980); questionably: *Cyprideis obliquosulcata Muñoz-Torres, Whatley & Van Harten, 1998.

**Characters.** Shape: subovate, elongated-ovate or subtrapezoidal. Ornament: smooth (except normal pore openings); asulcate (except *C. *obliquosulcata*). Marginal spines/denticles: without marginal spines (except *C*. *amazonica* and juveniles of *C*. *machadoi* plus *C. *caraionae* and *C. *obliquosulcata*). Inner lamella: very to moderately wide; all with vestibulum (except *C*. *simplex*, *C. *obliquosulcata* and *C. *caraionae* according to original description). Marginal pore canals (anterior): short to long; basically bifurcated or polyfurcated, some simple. Hinge: generotypic; some with very short median element (*C*. *multiradiata*); sometimes (*C*. *kotzianae*) or constantly (*C*. *kroemmelbeini*, *C*. *paralela*, *C*. *simplex*) inverse hinges.

#### 4.3.2 Nominal species and emended characteristics of the “ornate” *Cyprideis* group

**Species** (generic assignment sensu Whatley *et al.* 1998; alphabetic order; *not among the current material): *Cyprideis curucae* nom. nov. [= *Sohnicythere tuberculata* Purper & Pinto, 1983; syn. *Cyprideis* sp. 1–2 of Muñoz-Torres *et al.* 1998 = *Cyprideis* sp. 2–3 of Whatley *et al.* 1998]; *Cyprideis cyrtoma* Muñoz-Torres, Whatley & Van Harten, 1998; *Cyprideis graciosa* (Purper, 1979); *Cyprideis inversa* (Purper & Pinto, 1983); *Cyprideis ituiae* **n. sp.;** *Cyprideis longispina (Purper, 1979); *Cyprideis matorae* **n. sp.** [?syn. *Cyprideis* sp. 3 of Linhares *et al.* 2011]; *Cyprideis minipunctata* (Purper & Ornellas, 1991) [?junior syn. *Cyprideis purperi purperi* Sheppard & Bate, 1980; re-examination pending]; *Cyprideis munoztorresi* nom. nov. [= *Cyprideis lacrimata* Muñoz-Torres, Whatley & Van Harten, 1998 sensu Ramos 2006]; *Cyprideis pebasae* (Purper, 1979) [syn. *Cyprideis lacrimata* Muñoz-Torres, Whatley & Van Harten, 1998]; *Cyprideis reticulopunctata* (Purper, 1979); *Cyprideis schedogymnos* Muñoz-Torres, Whatley & Van Harten, 1998; *Cyprideis sulcosigmoidalis* (Purper, 1979) [syn. *Cyprideis aulakos* Muñoz-Torres, Whatley & Van Harten, 1998; pars syn. *Cyprideis purperi purperi* Sheppard & Bate, 1980]; probably: *Cyprideis anterospinosa Purper & Ornellas, 1991; *Cyprideis krsticae Purper & Pinto, 1985; *Cyprideis marginuspinosa (Purper & Ornellas, 1991), *Cyprideis retrobispinosa Purper & Pinto, 1983 [for all re-examination pending]; status unclear: *Cyprideis purperi colombiaensis Sheppard & Bate, 1980; re-examination pending].

**Characters.** Shape: subrectangular, subordinately subtriangular, subtrapezoidal or subovate. Ornament: punctate to reticulate; some with reduced ornament (*C. *krsticae*, *C*. *schedogymnos*, variants of *C*. *cyrtoma* and *C*. *sulcosigmoidalis*); mainly sulcate. Marginal spines/denticles: essentially all with anteromarginal spines/denticles on both valves (in *C*. *sulcosigmoidalis* sometimes reduced); all with posteroventral spines in right valves (except *C*. *inversa*, *C. *krsticae* and *C. *retrobispinosa*; due to reversed overlap in left valves); posteroventral spines in both valves in some species (*C*. *graciosa*, *C*. *matorae*, *C*. *reticulopunctata*). Inner lamella: moderately wide (except *C. curucae* where it is rather wide); all avestibulate. Marginal pore canals: simple, bifurcate (never polyfurcate). Hinge: generotypic; sometimes inverse hinges (*C*. *inversa* plus *C. *krsticae*, *C. *retrobispinosa*).

### 4.4 Western Amazonian *Cyprideis* species subgroups

Several “sublineages” were suggested by Whatley *et al.* (1998), later modified by Muñoz-Torres *et al.* (2006; Fig. 3a).

“Ornate lineage”: *C*. *sulcosigmoidalis* gave rise to the following subgroups: i) *C*. *longispina*, *C*. *minipunctata* and *Cyprideis* sp. 5 (the latter never described or illustrated); ii) *C*. *graciosa*, *C*. *lacrimata*, *C*. *pebasae*, *C*. *retrobispinosa*, *Cyprideis* sp. 1, iii) *C*. *inversa*, *Cyprideis* sp. 2; and, iv) *C*. *cyrtoma*, *Cyprideis* sp. 4 (the latter never described or illustrated).

“Smooth lineage”: From *C*. *machadoi* evolved: i) *C*. *caraionae*, *C*. *amazonica*; ii) *C*. *aulakos*, *C*. *schedogymnos*, *C*. *obliquosulcata*, *Cyprideis* sp. 3 (the latter never described or illustrated); and, iii) *C*. *olivencai*.

Due to the current taxonomic revision, these sublineages are challenged (for details see taxonomic treatment of species in chapters 4.5. and 4.6.). For instance: *C*. *aulakos* (= synonym to *C*. *sulcosigmoidalis*) and *C*. *schedogymnos* (very similar to *C*. *cyrtoma*) are transferred to the “ornate” group here. *C*. *olivencai* sensu Muñoz-Torres *et al.* (1998) and sensu Whatley *et al.* (1998) comprises the valid taxa *C*. *multiradiata*, *C*. *paralela* and *C*. *simplex*. Among *C*. *machadoi*, *C*. *kotzianae* and *C*. *kroemmelbeini* were included, which are valid species; and, *C*. *kroemmelbeini* is virtually much closer to *C*. *olivencai*. *Cyprideis* sp. 1 (placed in the “*graciosa*” sublineage) and *Cyprideis* sp. 2 of Muñoz-Torres *et al.* (1998; referred to the “*inversa*” sublineage) actually belong to the same species (*C*. *curucae*). While *C*. *lacrimata* of Muñoz-Torres *et al.* (1998) and Whatley *et al.* (1998) is synonymous with *C*. *pebasae*, *C*. *pebasae* Muñoz-Torres *et al.* (1998) and sensu Whatley *et al.* (1998) is a discrete species, renamed here as *C*. *munoztorresi*. Apparently, these taxonomic changes affect not only possible phylogenetic relations but also the biostratigraphic concept of Muñoz-Torres *et al.* (2006; see chapter 5.1.).

As far as the current stratigraphy is settled down, core 1AS-10-AM comprises only late Middle and, possibly, early Late Miocene sediments (see chapter 5.1.). Thus, a short time-range (probably <2 Ma) of the entire evolutionary history of Amazonian *Cyprideis* is covered here, which hampers phylogenetic considerations. Hence, a revision of the currently available phylogeny for Amazonian *Cyprideis* is beyond our possibilities. Nevertheless, we decided to arrange the found species into (sub-)groups, exclusively based on their morphologic similarity (Fig. 3b). The “smooth” group comprises the *amazonica*, *machadoi*, *olivencai* and *paralela* subgroups, and the “ornate” *Cyprideis* species the *cyrtoma*, *graciosa* and *pebasae* subgroups. Among the “smooth” taxa, *C*. *multiradiata* as well as among the “ornate” species *C*. *inversa* and *C*. *sulcosigmoidalis* were not attributed to subgroups due to their impartial morphology.

### 4.5 “Smooth” *Cyprideis* group

#### 4.5.1 *amazonica* subgroup

**Species.** *C*. *amazonica* and *C*. *caraionae* (the latter not among the present material).

**Characters.** Subovate; medium sized; smooth, asulcate; with or without anteromarginal denticles, short posteroventral denticles in right valves; moderately wide inner lamella, narrow anterior vestibulum; anterior simple and some bifurcated marginal pore canals; hinge with short antero- and long posteromedian element.

***Cyprideis amazonica* Purper, 1979**

Figs. 4a–c; Pl. 1, Figs. 1–26

**Table:** 

	1977 *Cyprideis* sp.nov. B—Purper: 363; Pl. 3, Figs. 11–16.
*	1979 *Cyprideis amazonica* Purper, sp. nov.—Purper: 231–232; Pl. 4, Figs. 1–11.
	1998 *Cyprideis amazonica* Purper, 1979—Muñoz-Torres *et al.*: 94; Pl. 2, Figs. 4–6.
	1998 *Cyprideis amazonica* Purper, 1979—Whatley *et al.*: 234; Text-fig. 2; Pl. 1, Figs. 1–5.
non	1998 *Cyprideis amazonica* Purper—Swain: 3; Pl. 2, Fig. 7.
	2010 *Cyprideis amazonica* Purper, 1979—Wesselingh & Ramos: 308, 315; Figs. 18.5k–l.
	2011 *Cyprideis amazonica*—Linhares *et al.*: 95, 98; Figs. 3/1–2.

**Material.** 1,083 valves; samples AM10/4, 6, 7, 39, 40 (in AM10/6 and 7 about four times more material available but not counted).

**Dimensions** (total range over all samples). R♀ l = 0.72–0.90 (0.82), h = 0.40–0.47 (0.44; n = 14); L♀ l = 0.76–0.89 (0.83), h = 0.44–0.51 (0.47; n = 13); R♂ l = 0.85–0.96 (0.91), h = 0.43–0.47 (0.45; n = 8); L♂ l = 0.84–0.96 (0.91), h = 0.46–0.50 (0.48; n = 7); Rj(A-1) l = 0.59–0.69 (0.65), h = 0.35–0.38 (0.37; n = 11); Lj(A-1) l = 0.56–0.70 (0.65), h = 0.35–0.40 (0.38; n = 9); Lj(A-2) l = 0.53, h = 0.33 (n = 1).

**Figure 4:**
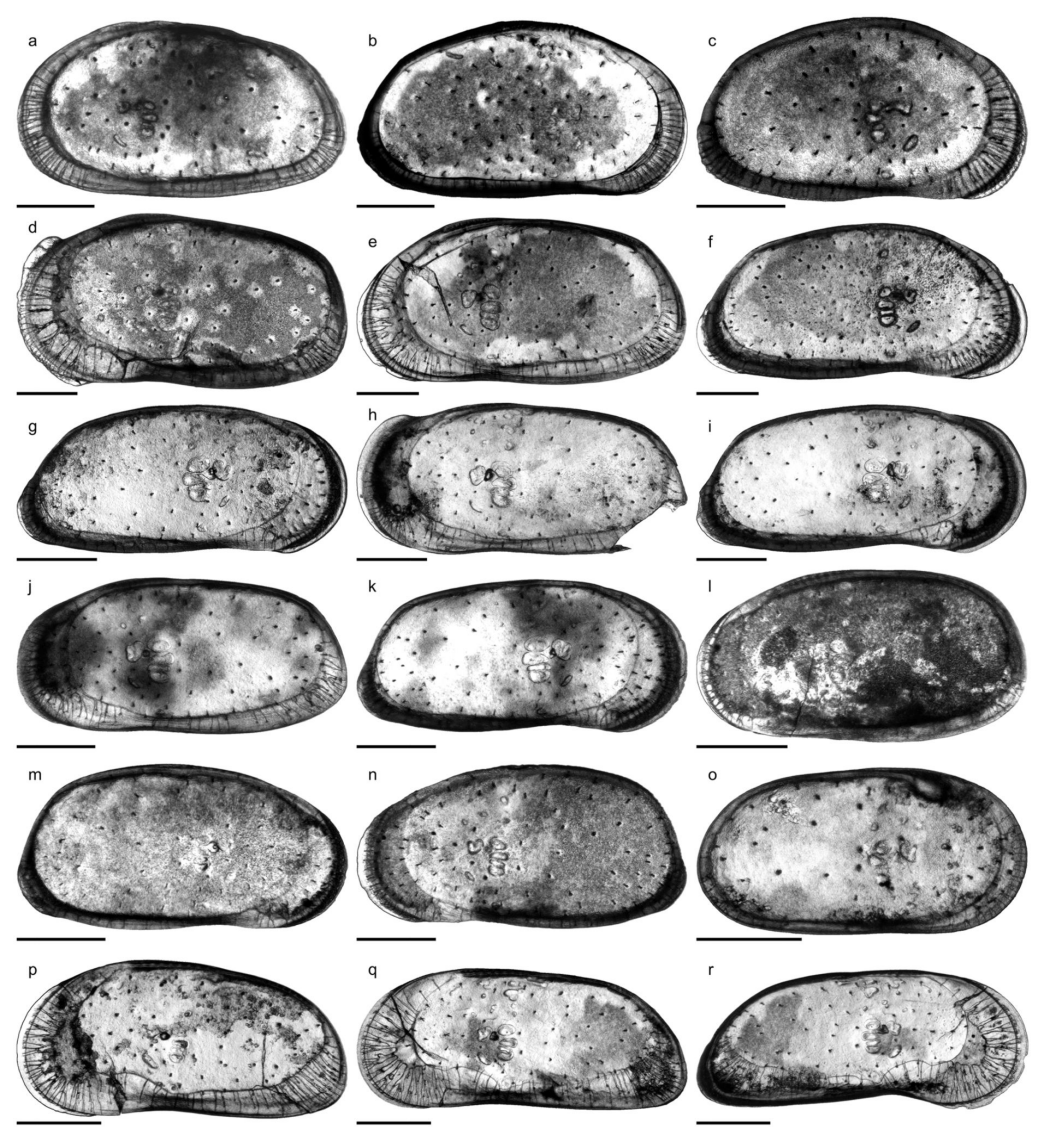
“Smooth” *Cyprideis* species of core 1AS-10-AM (except Figs. j–k = core 1AS-32-AM) photographed in transmitted light (external view) and focus stacked (scale bar = 200 µm; L/R = left/right valve; ♀/♂ = female/male; l/h = length/height [mm], specimen code = sample number_specimen number). a–c: *C*. *amazonica*, a: L♂ (0.84/0.46; AM10-39_44), b: R♀ (0.85/0.46; AM10-7_94), c: R♀ (0.74/0.42; AM10-39_42); d–f: *C*. *machadoi*, d: L♀ (1.09/0.58; AM10-23_66), e: L♀ (1.06/0.53; AM10-27_74), f: R♂ (1.08/0.51; AM10-27_73); g–k: *C*. *kotzianae*, g: R♂ (0.83/0.38; AM10-27_75), h: L♀ (1.00/0.46; AM10-30_36), i: R♂? (0.95/0.43; AM10-30_117), j: L♀ (0.87/0.41; AM32-5_26), k: R♀ (0.86/0.40; AM32-5_39); l–m: *C*. *olivencai*, l: L♀ (0.73/0.38; AM10-7_97), m: R♀ (0.75/0.38; AM10-7_96); n: *C*. *kroemmelbeini*, L♀ (0.83/0.41; AM10-7_100); o: *C*. *paralela*, R♀ (0.61/0.30; AM10-30_100); p–r: *C*. *multiradiata*, p: L♀ (0.78/0.37; AM10-30_116), q: L♂ (0.89/0.38; AM10-30_114), r: R♂ (0.89/0.37; AM10-30_113).

**Remarks.** *C*. *amazonica* is an asulcate species with a smooth surface except well-spaced, roundish puncta, which correspond to normal pore canal openings. Right valves have a “well developed posterior rim” (Muñoz-Torres *et al.* 1998: 234 = “postero-lateral concavity” in Purper 1979: 232). Both valves develop a narrow anterior vestibulum (Figs. 4a–c). Marginal pore canals are simple or—more frequently—bifurcating.

*C*. *amazonica* occurs in core 1AS-10-AM only in the lower (AM10/40–39) and in the upper part (AM10/7–6, 4). Especially, AM10/39 and 10/7 contained rich material, about 147 m apart from each other.

The material from samples AM10/7–6 matches well with the given synonyms (Pl. 1, Figs. 1–8). Valves from sample AM10/40–39 are slightly smaller, somewhat more roundish in outline and the extension of the flange at the lower half of the posterior margin is reduced (Pl. 1, Figs. 9–16). Here, we consider these differences as intraspecific variation of *C*. *amazonica*.

*C*. *amazonica* figured by Swain (1998) diverges considerably in outline and clearly belongs to another *Cyprideis* species.

In addition to the descriptions of Purper (1979), Muñoz-Torres *et al.* (1998) and Whatley *et al.* (1998), we observed in well-preserved right valves some (up to six) short posteroventral denticles (Pl. 1, Fig. 25b). Interestingly, in larvae—which have never been described or figured so far—these posteroventral denticles are more clearly visible (Pl. 1, Fig. 26b) and become almost fused with the flange in the adult stadium.

Moreover, juvenile valves from AM10/39 exhibit numerous anteroventral denticles (Pl. 1, Fig. 26a), which are incorporated in the selvage–flange zone in adult valves where only traces in form of marginal ripplets are left over (Pl. 1, Fig. 25a). Anteroventral marginal spines are missing in juvenile valves from the upper part of the core (Pl. 1, Fig. 20). Very faint crenulations of the flange could be a reminiscence of them, observable in some valves of AM10/7.

At first glance, the anteroventral denticles resemble the dentate margin of juveniles of *Cyprideis caraionae* Purper & Pinto, 1985, which is in its adult stage similar in outline and ornament to *C*. *amazonica* (Whatley *et al.* 1998). However, larvae of *C*. *caraionae* from topotypic material (1AS-33-AM; sample depth 290.1 m) display another kind of anterior denticle development. They have fewer, longer and more widely spaced denticles, in which further in-between denticles develop in the adult stadium in order to become the characteristic downward-turned, coalescent spines of *C*. *caraionae* (pers. observ., M.G.). Further investigations are obviously needed. Here, we just want to highlight the importance of the study of the ontogeny that may help determine relationships among different taxa.

**Occurrence.** Western Amazonia (Brazil, Colombia, Peru), early Middle to early Late Miocene (*C*. *aulakos–C*. *cyrtoma* zone; Muñoz-Torres *et al.* 2006; chronostratigraphic correlation after Wesselingh & Ramos 2010).

#### 4.5.2 *machadoi* subgroup

**Species.** *C*. *machadoi* and *C*. *kotzianae*; possibly *C*. *truncata* but re-examination pending.

**Characters.** Elongated-subtrapezoidal, with or without anterior “*Chlamydotheca*”-like extension; medium to large sized; smooth, asulcate; no marginal spines (in adults); very wide inner lamella with well-developed anterior vestibulum; anterior simple, bi- or polyfurcated marginal pore canals; hinge with moderately long antero- and long posteromedian element, sometimes inverse.

***Cyprideis machadoi* (Purper, 1979)**

Figs. 4d–f; Pl. 1, Figs. 27–41; Pl. 2, Figs. 1–23

**Table:** 

	1977 Ostracoda B n.g.,n.sp.—Purper: 359; Pl. 1, Figs. 9–14.
*	1979 *Chlamydocytheridea machadoi* Purper, gen. et sp. nov.—Purper: 237–238; Pl. 6, Figs. 1–6.
?	1979 *Cyprideis truncata* Purper, sp. nov.—Purper: 232–233; Pl. 4, Figs. 12–22.
non	1979 *Paulacoutoia kroemmelbeini* Purper, sp. nov.—Purper: 236–237; Pl. 5, Figs. 18–24.
	1980 *Otarocyprideis elegans* sp. nov.—Sheppard & Bate: 101–102; Pl. 8, Figs. 10–12; Pl. 9, Figs. 1–5, 7.
	1983 *Chlamydocytheridea machadoi* Purper, 1979—Purper & Pinto: 114; Pl. 1, Figs. 14–17.
pars	1991 *Chlamydocytheridea kotzianae* Purper & Ornellas, sp. nov.—Purper & Ornellas: 26; Pl. 1, Fig. 7. [non Pl. 1, Figs. 8–9].
	1998 *Cyprideis machadoi* (Purper, 1979)—Muñoz-Torres *et al.*: 98; Pl. 3, Figs. 15–17.
	1998 *Cyprideis machadoi* (Purper, 1979)—Whatley *et al.*: 235; Text-fig. 2; Pl. 2, Figs. 6–10 [sic].
?	2010 *Cyprideis machadoi* (Purper, 1979)—Wesselingh & Ramos: 308; Figs. 18.5m–n.
	2011 *Cyprideis machadoi*—Linhares *et al.*: 95; Figs. 3/13–14.
?	2013 *Cyprideis* aff. *machadoi* (Purper, 1979)—Gross *et al.*: 227–229; Pl. 6, Figs. 1–20, 22.

**Material.** 1,031 valves; samples AM10/15–16, 19, 21–31, 35, 40–42, 48.

**Dimensions** (total range over all samples). R♀ l = 0.90–1.25 (1.03), h = 0.46–0.64 (0.53; n = 95); L♀ l = 0.92–1.26 (1.08), h = 0.50–0.67 (0.58; n = 17); R♂ l = 1.08–1.39 (1.22), h = 0.51–0.66 (0.57; n = 8); L♂ l = 1.10–1.38 (1.25), h = 0.55–0.68 (0.61; n = 3); Rj(A-1) l = 0.69–0.90 (0.80), h = 0.37–0.47 (0.41; n = 5); Rj(A-2) l = 0.59–0.65, h = 0.28–0.33 (0.31; n = 7); Lj(A-1) l = 0.70–0.81 (0.77), h = 0.36–0.45 (0.41; n =3).

**Remarks.** These valves coincide with the type material of Purper (1977, 1979), with the junior synonym *O. elegans* of Sheppard & Bate (1980; see Purper & Pinto 1985) as well as with the specimens from Purper & Pinto (1983), Muñoz-Torres *et al.* (1998), Whatley *et al.* (1998) and Linhares *et al.* (2011).

**Figure 5:**
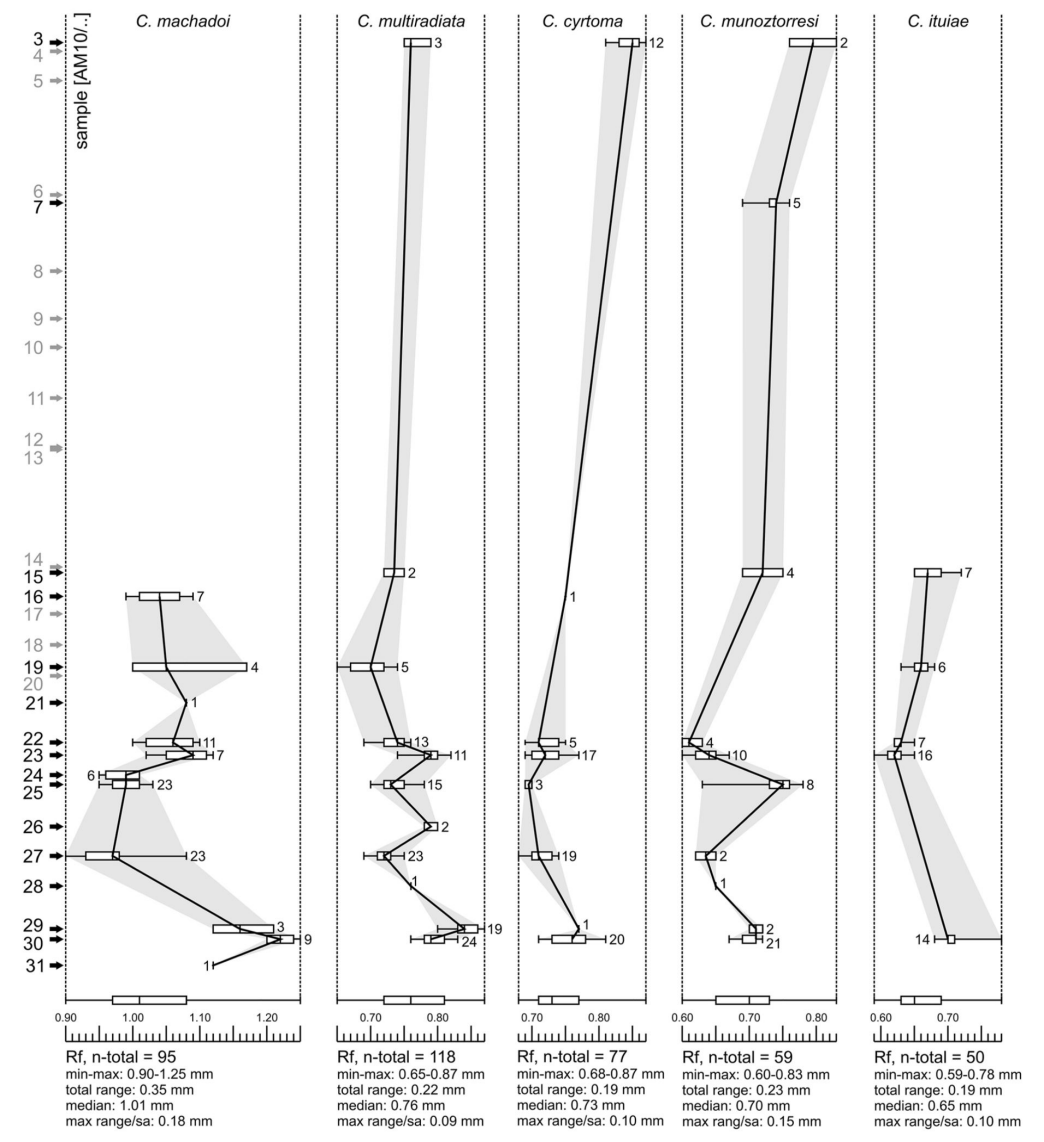
Variation in length of right female valves (Rf) in core 1AS-10-AM of some more frequent *Cyprideis* species (min = minimum, max = maximum, max range/sa = maximum range within one sample; aside the box plots the number of measured specimens per sample is indicated).

Muñoz-Torres *et al.* (1998: 98) characterise the very large, smooth, asulcate *C*. *machadoi* as “extremely variable, particularly in the curvature and width of the anterior part of the right valve, in the degree of development of the flange and in variation in the shape and outline of the anterior margin of the left valve, where a “*Chlamydotheca*”-like extension may or may not be present, or may be only partly developed; a reversed hinge occurs in some specimens.” Similarly, our observations demonstrate a strong variability of the “*Chlamydotheca*”-like flange in left valves, which goes along with substantial changes in outline (e.g. well-developed flange (AM10-23; Pl. 1, Figs. 27, 30), poorly developed flange (AM10/27; Pl. 2, Figs. 5, 8), intermediately developed flange (AM10/30; Pl. 2, Figs. 14, 17)). Additionally, considerable variations in valve size are evident between samples (Fig. 5 and figures on Pl. 1–2). Remarkably, right juvenile valves exhibit a tiny denticulation posteroventrally as well as one short posteroventral spine (Pl. 1, Figs. 37, 39 and especially 40). These denticles become integrated in the posteroventral flange extension in the adult stadium (Pl. 1, Fig. 41).

Muñoz-Torres *et al.* (1998) put *Paulacoutoia kroemmelbeini* Purper, 1979, *Chlamydocytheridea kotzianae* Purper & Ornellas, 1991 (both transferred to *Cyprideis*) and *Cyprideis truncata* Purper, 1979 into the synonymy of *C*. *machadoi*.

However, *C*. *kroemmelbeini* is more closely related to *Cyprideis olivencai* (Purper, 1979) (see chapter 4.5.3.) and cannot be included in *C*. *machadoi*. Compared to *C*. *machadoi*, *C*. *kroemmelbeini* and *C*. *olivencai* (see Pl. 4, Figs. 1–29) are much smaller, have an different outline (i.e., greatest height behind the ventral concavity; dorsal margin less sloping backwards), the anterior vestibulum is much larger, marginal pore canals are much shorter, simple or only anteroventrally branched or ramified (Figs. 4l–n), and the median hinge elements are shorter.

*C*. *kotzianae* is a valid taxon and only parts of the type series of this species belong to *C*. *machadoi* (see below).

The material from the Late Miocene of Eirunepé (Wesselingh & Ramos 2010; Gross *et al.* 2013) is close to *C*. *machadoi*, especially to the specimens from samples AM10/25 and 24. However, as reported by Gross *et al.* (2013), the Eirunepé valves are smaller, anterior and posterior hinge elements are weaker, the inner lamella is less wide, the branched character of the marginal pores is less developed and the area between flange and selvage anterior is narrower. Gross *et al.* (2013) discussed a possible synonymy of the Eirunepé specimens with *C*. *truncata*, which, however, needs to be reinvestigated. Based on the documented variability here, it is possible that *C*. *truncata* belongs to *C*. *machadoi*—at least in a wider sense.

**Occurrence** (of *C*. *machadoi* sensu Muñoz-Torres *et al.* 1998). Western Amazonia (Brazil, Colombia, Peru), early Middle to early Late Miocene (*C*. *aulakos–C*. *cyrtoma* zone; Muñoz-Torres *et al.* 2006; chronostratigraphic correlation after Wesselingh & Ramos 2010).

***Cyprideis kotzianae* (Purper & Ornellas, 1991)**

Figs. 4g–k; Pl. 3, Figs. 1–38

pars *1991 *Chlamydocytheridea kotzianae* Purper & Ornellas, sp. nov.—Purper & Ornellas: 26; Pl. 1, Figs. 8–9. [non Pl. 1, Fig. 7; holotype]

**Material.** 207 valves; samples AM10/3, 15, 19, 23–25, 27–30, 40–41.

**Dimensions** (total range over all samples; inverse forms not differentiated). R♀ l = 0.82–1.04 (0.93), h = 0.39–0.50 (0.45; n = 9); L♀ l = 0.79–1.00 (0.92), h = 0.40–0.49 (0.45; n = 8); R♂ l = 0.81–0.99 (0.89), h = 0.36–0.45 (0.41; n = 5); L♂ l = 0.81–1.01 (0.92), h = 0.38–0.46 (0.42; n = 4); Rj(A-1) l = 0.68, h = 0.34 (n = 1); Lj(A-1) l = 0.67–0.71 (0.69), h = 0.33–0.35 (0.34; n = 2).

**Remarks.** *C*. *kotzianae* is similar to *C*. *machadoi*, which stimulated Muñoz-Torres *et al.* (1998) and Whatley *et al.* (1998) to synonymise both species.

In core 1AS-10-AM both species co-occur with males, females and juveniles in several samples. For this reason and the following differences, we consider this species as a valid taxon: *C*. *kotzianae* is smaller; has a more elongated outline with a more equally rounded anterior margin; the hinge elements are weaker developed and characteristically, the selvage runs anteroventrally over a short distance in a straight course in normal right valves and inverse left valves, respectively (compare *C*. *machadoi*: Pl. 2, Fig. 21 with *C*. *kotzianae*: Pl. 3, Fig. 38). Although the vestibulum varies to some degree in *C*. *machadoi*, the anterior vestibulum is much larger in *C*. *kotzianae* (Figs. 4g–k). Due to a narrower fused zone in *C*. *kotzianae*, marginal pore canals are rather short and simple or bifurcated. In *C*. *machadoi*, the anterior marginal pore canal openings are large and open between the selvage and flange, flanked by typical marginal ripplets (e.g. Pl. 2, Fig. 21), which are constantly missing in *C*. *kotzianae* (e.g. Pl. 3, Fig. 38).

Similar to *C*. *machadoi*, the development of the “*Chlamydotheca*”-like flange (in normal left and inverse right valves, correspondingly) as well as size vary between samples (e.g. Pl. 3, Fig. 21, 33 (AM10/30): well-developed flange, large; Pl. 3, Fig. 14, 32 (AM10/25): less developed, small).

**Figure 6:**
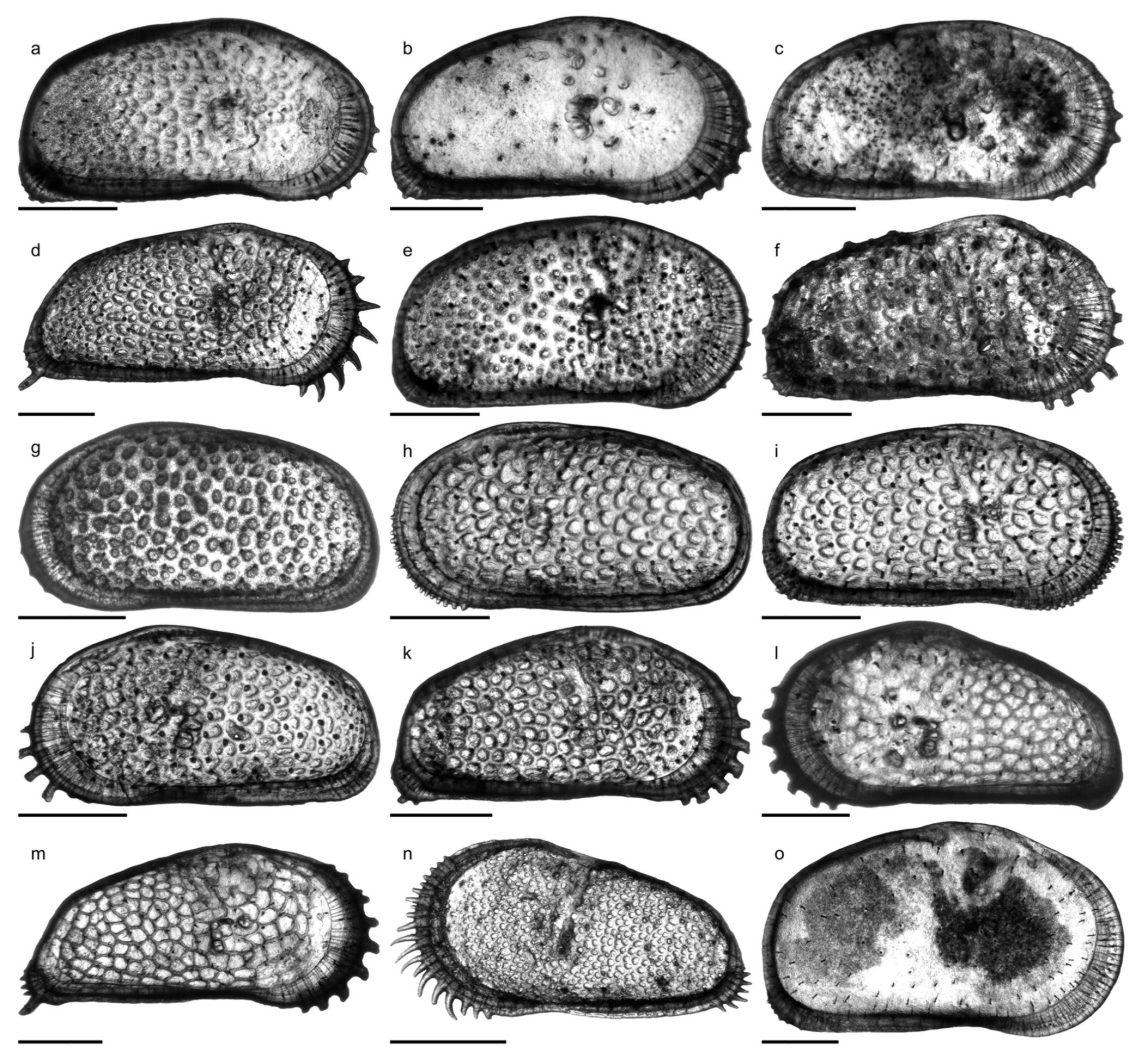
“Ornate” *Cyprideis* species of core 1AS-10-AM photographed in transmitted light (external view) and focus stacked (scale bar = 200 µm; for abbreviations, see Fig. 4). a–b: *C*. *cyrtoma*, a: R♀ (0.73/0.37; AM10-27_15), b: R♀ (0.79/0.41; AM10-30_51); c: *C*. *schedogymnos*, R♀ (0.77/0.39; AM10-42_20); d: *C*. aff. *graciosa*, R♂ (0.95/0.48; AM10-30_68); e: *C*. *minipunctata*, R♀ (0.80/0.44; AM10-42_26); f: *C*. *curucae*, R♀ (0.80/0.42; AM10-23_06); g: *C*. *pebasae*, L♀ (0.67/0.36; AM10-27_79); h–i: *C*. *munoztorresi*, h: L♀ (0.74/0.39; AM10-15_23), i: R♀ (0.75/0.38; AM10-15_25); j–k: *C*. *ituiae*, j: L♀ (0.64/0.33; AM10-23_105), k: R♀ (0.68/0.35; AM10-30_118); l–m: *C*. *matorae*, l: L♀ (0.80/0.43; AM10-30_121), m: R♂ (0.81/0.39; AM10-30_120); n: *C*. *inversa*, L♂ (0.58/0.32; AM10-30_122); o: *C*. *sulcosigmoidalis*, R♀ (1.01/0.59; AM10-7_47).

**Figure 7:**
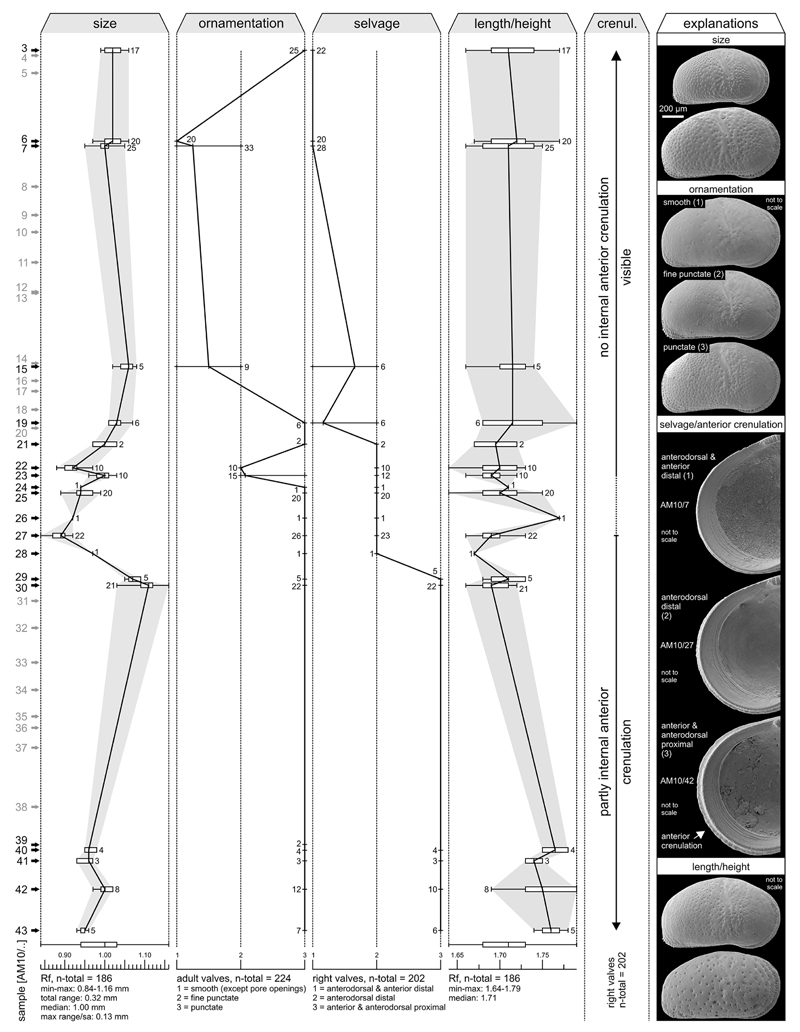
Variation of some traits in *C*. *sulcosigmoidalis* (for ornamentation and selvage position specimens were assigned to classes (1–3; ESM 2); if more than one class occur in one sample, the arithmetic mean is also displayed; the number of studied specimens per sample is indicated next to the bars; min = minimum, max = maximum, max range/sa = maximum range within one sample, Rf = right female valves).

**Figure 8:**
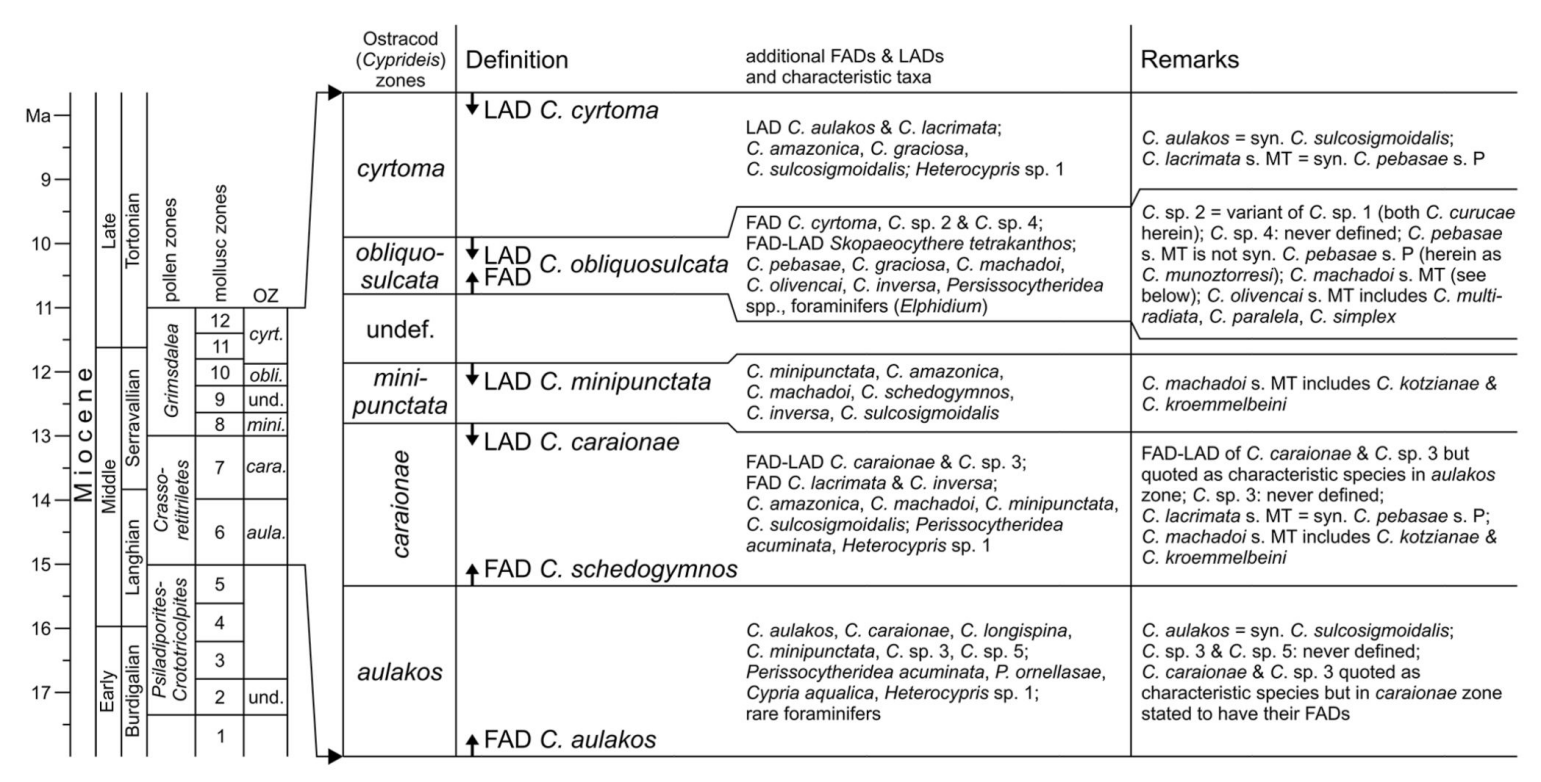
Ostracod based biozonation of Muñoz-Torres *et al.* (2006) and remarks (correlations of biozones and chronostratigraphy after Wesselingh *et al.* 2006b; Wesselingh & Ramos 2010; FAD = first appearance datum and LAD = last appearance datum sensu Muñoz-Torres *et al.* (2006); s. MT = sensu Muñoz-Torres *et al.* (1998), s. P = sensu Purper (1979), syn. = synonymous).

**Figure 9:**
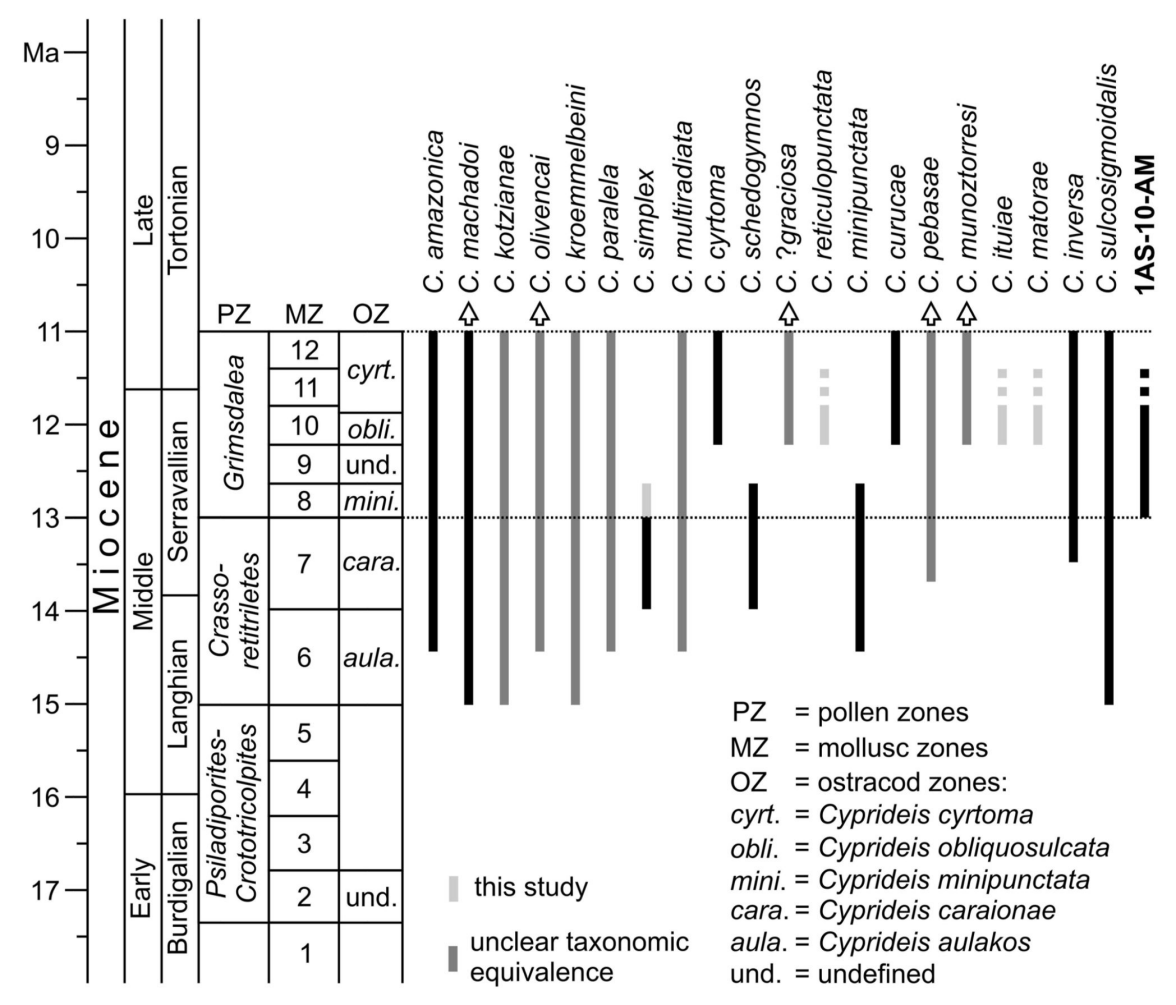
Stratigraphic range of herein treated *Cyprideis* species mainly based on Muñoz-Torres *et al.* (2006; for details see chapters 4.5. and 4.6.; correlations of biozones and chronostratigraphy after Wesselingh *et al.* 2006b; Wesselingh & Ramos 2010; arrows: probably further extending into the Late Miocene, see Gross *et al.* 2013).

**Figure 10:**
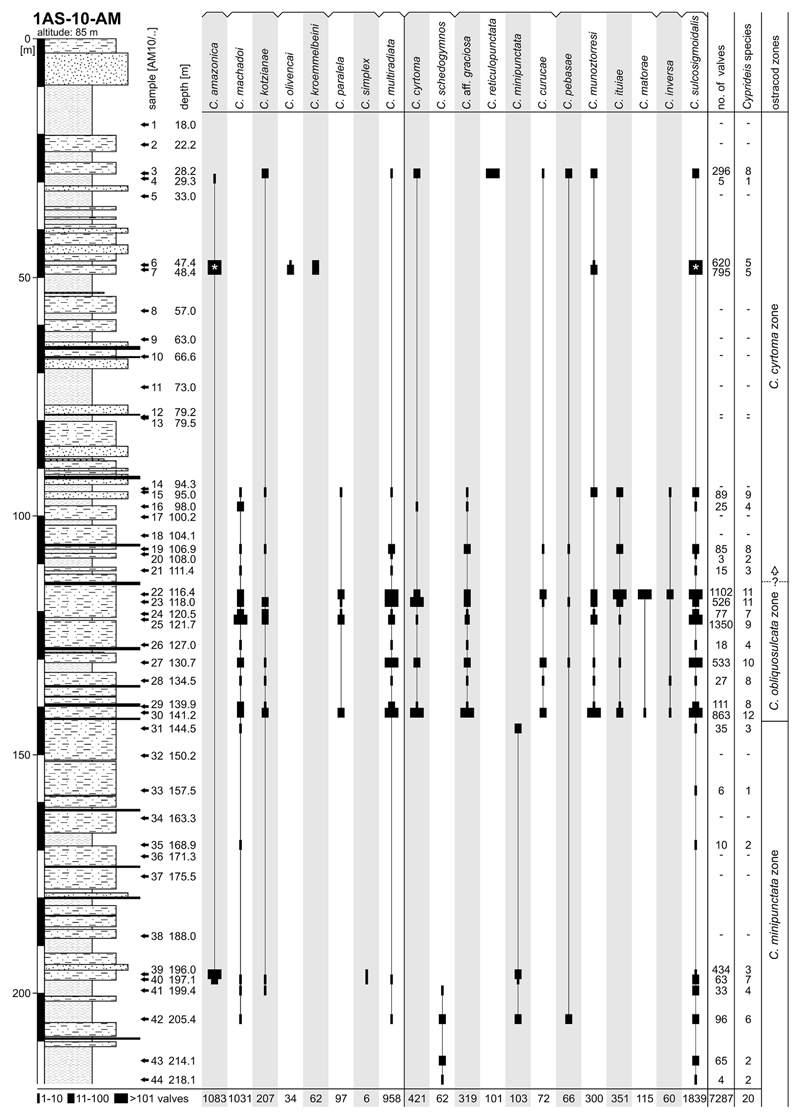
Distribution of *Cyprideis* species in core 1AS-10-AM and ostracod biozonation (barren samples AM10/45–47 as well as the single valve in AM10/48 not displayed; asterisk at samples AM10/6–7: more (~four times) material available but not counted; for legend of the lithology, see Fig. 1).

In some samples (AM10/23, 3; Pl. 3, Figs. 1–11) only inverse specimens occur (but never associated with regular valves), which are otherwise identical with normal valves of *C*. *kotzianae*. Since an inverse hinge (and reversed valve overlap) is the only difference, we consider this feature as intraspecific variability (but compare *C*. *kroemmelbeini*; see chapter 4.5.3.). Interestingly, inverse *C*. *machadoi*-valves have never been observed in the current material. Thus, the hint to inverse hinges in the description of *C*. *machadoi* in Muñoz-Torres *et al.* (1998) most probably refers to inverse *C*. *kotzianae*-specimens.

Purper & Ornellas (1991) established *C*. *kotzianae* based on material from core 1AS-32-AM (sample depth: 26 m). The holotype—a female left valve (MP-O-1237; Pl. 1, Fig. 7 in Purper & Ornellas 1991)—equals *C*. *machadoi* with a well-developed “*Chlamydotheca*”-like flange from the present core (e.g. Pl. 1, Fig. 27). On the other hand, the “male” paratype (carapace MP-O-1238; Pl. 1, Figs. 8–9 in Purper & Ornellas 1991) is identical with *C*. *kotzianae* females of our material (e.g. Pl. 3, Fig. 21 and Fig. 10, an inverse specimen, if mirrored).

We re-sampled the type layer (as far as possible) of core 1AS-32-AM (sample depth: 27.3 m) and found *C*. *kotzianae* associated with *C*. *machadoi*—analogous as in core 1AS-10-AM (unfortunately, only variants with less developed flange but identical with specimens from AM10/27, 25; compare e.g. *C*. *kotzianae* from the “type layer” on Pl. 3, Figs. 12–13 with specimens from AM10/25 on Pl. 3, Figs. 14, 32). We assume that both species have been mixed up during the description of *C*. *kotzianae*. Consequently, the female specimen (the holotype; MP-O-1237) belongs to *C*. *machadoi*; it has to be rejected as holotype for *C*. *kotzianae* and is excluded from the type series. Nonetheless, *C*. *kotzianae* is a distinct, sufficiently described nominal taxon to which the specimens under discussion here belong. We suggest the paratype MP-O-1238 of Purper & Ornellas 1991 (which we believe is a female) as neotype for *C*. *kotzianae* in order to clarify the taxonomic status of this species (ICZN 72.4.5, 75.3, recommendation 75A; MP-O-1238 is stored in the collection of the Universidade Federal do Rio Grande do Sul, Porto Alegre).

Finally, *Chlamydocytheridea* is considered by Whatley *et al.* (1998) as a junior synonym of *Cyprideis*. Here we follow their opinion with reservation and *Chlamydocytheridea kotzianae* Purper & Ornellas, 1991 turns into *Cyprideis kotzianae* (Purper & Ornellas, 1991).

**Occurrence** (of *C*. *machadoi* sensu Muñoz-Torres *et al.* 1998). Western Amazonia (Brazil, Colombia, Peru), early Middle to early Late Miocene (*C*. *aulakos–C*. *cyrtoma* zone; Muñoz-Torres *et al.* 2006; chronostratigraphic correlation after Wesselingh & Ramos 2010). Up to now only distinguished in Brazil (core 1AS-32-AM, depth: 26 m, altitude: 71 m; Purper & Ornellas 1991; plus depth: 27.3 and 121.3 m, pers. observ., M.G.).

#### 4.5.3 *olivencai* subgroup

**Species.** *C*. *olivencai* and *C*. *kroemmelbeini*.

**Characters.** Subovate–subtrapezoidal; medium sized; smooth, asulcate; no marginal spines; wide inner lamella with large anterior vestibulum; anterior short, branched or ramified (anteroventrally) marginal pore canals; hinge moderately long, approximately equally divided median element.

***Cyprideis olivencai* (Purper, 1979)**

Figs. 4l–m; Pl. 4, Figs. 1–8, 25, 28

**Table:** 

*	1979 *Paulacoutoia olivencai* Purper, gen. et sp. nov.—Purper: 235–236; Pl. 5, Figs. 10–17.
pars	1998 *Cyprideis olivencai* (Purper, 1979)—Muñoz-Torres *et al.*: 100; Pl. 4, Fig. 6. [non Pl. 4, Figs. 5, 7]
pars	1998 *Cyprideis olivencai* (Purper, 1979)—Whatley *et al.*: 236; Pl. 2, Figs. 1–2 [sic]. [non text-Fig. 2 (next to last row, right), non Pl. 2, Figs. 3–5 [sic]]
?	2010 *Cyprideis olivencai* (Purper, 1979)—Wesselingh & Ramos: 308; Figs. 18.5o–p.
?	2011 *Cyprideis olivencai*—Linhares *et al.*: 97; Figs. 4/1–2.
?	2013 *Cyprideis* ?*olivencai* (Purper, 1979)—Gross *et al.*: 229; Pl. 6, Figs. 21, 23–27.

**Material.** 34 valves; samples AM10/6–7.

**Dimensions** (total range over all samples). R♀ l = 0.75–0.80 (0.77), h = 0.38–0.41 (0.39; n = 7); L♀ l = 0.73–0.82 (0.77), h = 0.38–0.43 (0.41; n = 3); R♂ l = 0.76–0.79 (0.77), h = 0.35–0.36 (0.35; n = 3); L♂ l = 0.79, h = 0.42 (n = 1).

**Remarks.** The type material of Purper (1979) matches well with the present specimens, although a bit more elongated than the valves described here. We assume this slight difference in outline to be intraspecific variability.

Muñoz-Torres *et al.* (1998) and Whatley *et al.* (1998) synonymised several species under *C*. *olivencai* (*C*. *multiradiata*, *C*. *paralela*, *C*. *simplex*). Due to large and consistent differences (e.g. outline, inner lamella, hinge), we consider those three species as separate and valid taxa (see chapters 4.5.4. and 4.5.5.).

*C*. *olivencai* (sensu Purper (1979) and this work) is a smooth, asulcate, subovate–subtrapezoidal species with a wide anterior inner lamella, large vestibulum and narrow fused zone. Marginal pore canals are short, branched or ramified. The hinge (right valves) consists of denticulated, anterior long and posterior shorter elements. The crenulated, long median element is divided into a negative anteromedian and a positive posteromedian part. Sexual dimorphism is expressed by more elongated male valves with a more pointed posterior margin.

Figures given in Wesselingh & Ramos (2010) and Linhares *et al.* (2011) coincide well in outline with the current material. However, details (e.g. hinge) of the inner valve characters are not visible. Likewise, the rare specimens of Gross *et al.* (2013) correspond in outline, but have, quite unusually, smooth hinge elements.

*C*. *olivencai* is very similar to *C*. *kroemmelbeini* with which it co-occurs (see below). The most obvious divergence of *C*. *kroemmelbeini* is the inverse hinge and reverse right and left valve overlap respectively (constantly found in both sexes as well as in juveniles; Pl. 4, Figs. 9–24). By comparing the outlines of right valves of *C*. *olivencai* with mirrored (!) left valves of *C*. *kroemmelbeini* further differences are on hand. In left valves of the latter, the outline of the anterior proportion of the ventral margin displays a straight course over a short distance due to a slight projection of the flange (Purper 1979; Pl. 4, Figs. 25–28). Mirrored right valves of *C*. *kroemmelbeini* display a slightly more expressed ventral concavity in comparison to left valves of *C*. *olivencai* (compare Pl. 4, Fig. 2 (*C*. *olivencai*) with Pl. 4, Fig. 29 (*C*. *kroemmelbeini*, mirrored)).

If an inverse hinge and such rather subtle differences in outline are species diagnostic remains unclear (Kollmann 1960; Van Morkhoven 1962; Purper & Pinto 1985). It is possible that *C*. *kroemmelbeini* is synonymous with *C*. *olivencai* (but certainly not with *C*. *machadoi* as suggested by Muñoz-Torres *et al.* (1998) and Whatley *et al.* (1998)). Because we found two clearly differentiable morphotypes with females, males and juveniles within one sample, reproductively separated species are probable. For this reason, we decided to distinguish both forms here.

**Occurrence** (of *C*. *olivencai* sensu Muñoz-Torres *et al.* 1998). Western Amazonia (Brazil, Colombia, Peru), early Middle to early Late Miocene (*C*. *aulakos–C*. *cyrtoma* zone; Muñoz-Torres *et al.* 2006; chronostratigraphic correlation after Wesselingh & Ramos 2010).

***Cyprideis kroemmelbeini* (Purper, 1979)**

Fig. 4n; Pl. 4, Figs. 9–24, 26–27, 29

**Table:** 

*	1979 *Paulacoutoia kroemmelbeini* Purper, sp. nov.—Purper: 236–237; Pl. 5, Figs. 18–24.

**Material.** 62 valves; samples AM10/6–7.

**Dimensions** (total range over all samples). R♀ l = 0.81–0.84 (0.83), h = 0.43–0.44 (0.44; n = 2); L♀ l = 0.80–0.85 (0.83), h = 0.40–0.43 (0.42; n = 8); R♂ l = 0.84–0.88 (0.86), h = 0.41–0.43 (0.42; n = 4); L♂ l = 0.85–0.90 (0.88), h = 0.39–0.42 (0.41; n = 2); Rj(A-1) l = 0.64, h = 0.34 (n = 1); Lj(A-1) l = 0.62–0.69 (0.66), h = 0.32–0.36 (0.34; n = 2).

**Remarks.** The present specimens with their downward projecting anteroventral margin are identical in outline with *P*. *kroemmelbeini* as well as in all other characters. Purper (1979) did not explicitly mention an inverse hinge and had only two left valves available. However, based on her description as well as on the given figures (especially the dorsal views on Pl. 5, Figs. 19–20), an inverse hinge is probable (for differences from *C*. *olivencai* and *C*. *machadoi* see above).

Whatley *et al.* (1998) synonymised the genus *Paulacoutoia* Purper, 1979 with *Cyprideis*. By provisionally applying this concept, *P*. *kroemmelbeini* is named *Cyprideis kroemmelbeini* here.

**Occurrence** (of *C*. *machadoi* sensu Muñoz-Torres *et al.* 1998). Western Amazonia (Brazil, Colombia, Peru), early Middle to early Late Miocene (*C*. *aulakos–C*. *cyrtoma* zone; Muñoz-Torres *et al.* 2006; chronostratigraphic correlation after Wesselingh & Ramos 2010). Up to now only recognized in Brazil (core CPCAN-III-São Paulo de Olivença (depth: 19.50–20.78 m, altitude: ~33 m; Purper 1979).

#### 4.5.4 *paralela* subgroup

**Species.** *C*. *paralela*, *C*. *simplex*, *C*. *posterocompressus* (the latter not among the current material and possibly an extreme form of *C*. *paralela*).

**Characters.** Elongated-oval; small sized; smooth, asulcate; no marginal spines; moderately wide inner lamella with well-developed or reduced vestibulum, some with internal “eye-spot”; anterior long to short, bifurcated or simple pore canals; hinge: constantly inverse; rather short median element divided into a short anteromedian and an inconspicuous posteromedian element.

***Cyprideis paralela* (Purper, 1979)**

Fig. 4o; Pl. 4, Figs. 30–45

**Table:** 

*	1979 *Pseudoparakrithella paralela* Purper, gen. et sp.nov.—Purper: 238–239; Pl. 6, Figs. 7–14.
	1983 *Pseudoparakrithella paralela* Purper, 1979—Purper & Pinto: 118; Pl. 2, Figs. 14–16.
	1985 *Pseudoparakrithella paralela* Purper, 1979—Purper & Pinto: 427, 430; Fig. 2.

**Material.** 97 valves; samples AM10/15, 22–25, 30.

**Dimensions** (total range over all samples). R♀ l = 0.59–0.65 (0.62), h = 0.30–0.32 (0.31; n = 5); L♀ l = 0.62–0.67 (0.64), h = 0.30–0.32 (0.30; n = 5); R♂ l = 0.59–0.66 (0.63), h = 0.28–0.30 (0.29; n = 3); L♂ l = 0.57–0.63 (0.61), h = 0.28–0.29 (0.29; n = 4).

**Remarks.** These specimens belong to a group of rather small-sized *Cyprideis* species with approximately parallel dorsal and ventral margin, with an inverse hinge with long crenulated anterior and posterior bars, a short anteromedian crenulated groove and a short, crenulated, inconspicuous posteromedian tooth (left valves). The valves available here have an elongated-ovate shape in lateral view, are smooth (except normal pore canal openings), have a comparably small inner lamella with a narrow fused zone and simple marginal pore canals (a few branched canals can be observed on the anteroventral thickening of the inner lamella) and a well-developed vestibulum.

Variation in size and outline between samples is small as far as it can be examined on the limited material. However, specimens of AM10/30 (Pl. 4, Figs. 36–45) display a distinct internal “eye-spot” (Purper & Pinto 1985), which is missing in the samples up-section. Only in some badly preserved right valves of AM10/25 a slight thickening below the anterior crenulated hinge element may correspond to that remarkable structure.

Four species are comparable with the current material: *Botulocyprideis simplex* Sheppard & Bate, 1980, *Pseudoparakrithella paralela* Purper, 1979, *Nealecythere posterocompressus* Purper & Pinto, 1983 (Whatley *et al.* (1998) put these three genera into *Cyprideis*) and *Cyprideis* sp. 2 in Gross *et al.* (2013).

Muñoz-Torres *et al.* (1998) and Whatley *et al.* (1998) placed *C. simplex* and *C*. *paralela* as well as *Cyprideis multiradiata* (Purper, 1979) into the synonymy of *Cyprideis olivencai*. These authors used a broad species concept, which results in a “very variable” species definition. As shown in chapter 4.5.5., *C*. *multiradiata* is a valid taxon and cannot be joined with *C*. *olivencai*. Likewise, the species group around *C*. *paralela* (*C*. *simplex*, *C*. *paralela*, *C*. *posterocompressus*, *Cyprideis* sp. 2) forms a distinct lineage (Purper & Pinto 1985) and *C*. *simplex* and *C*. *paralela* are clearly not synonyms of *C*. *olivencai* (note: *C. paralela* (Purper, 1979) must not be confused with the Central European species *Cyprideis parallela* Krstić, 1968b).

*Cyprideis* sp. 2 from the Late Miocene of the Eirunepé region (Gross *et al.* 2013) differs from the core material by its coarsely punctate to reticulate ornamentation and its occasionally observable 2–3, inconspicuous posteroventral denticles. An eye-spot as well as the extension of the flange on the lower half of the posterior margin (left valves) are always missing.

*C*. *simplex* (see below) can be distinguished because it has no vestibulum, resulting in a wider anterior fused zone with more frequently bifurcated marginal pore canals (Sheppard & Bate 1980; Purper & Pinto 1983, 1985).

*C*. *posterocompressus* diverge insignificantly by a slightly narrower fused marginal zone and a somewhat wider vestibulum. These differences are vague compared to the variation observed within one sample between right and left valves (left valves seem to have a wider vestibulum than right valves). Due to the well-developed eye-spot, especially the specimens from AM10/30 are very close to *C*. *posterocompressus*. The main difference is that the current material lacks the diagnostic external posterior depression on left valves (Purper & Pinto 1983). Remarkably, already Purper & Pinto (1983) mentioned a transition of this trait between *C*. *posterocompressus* and *C*. *paralela* (pers. observ., M.I.F.R.: among the type material of *C. posterocompressus* only males display that depression; compare also Fig. 1 on plate 2 of Purper & Pinto (1983), where this feature appears to be lacking on the female carapace). Based on our material, we are not in the position to decide if the indistinct differences in the inner lamella, the expression of the eye-spot and the compressed posterior proportion in left valves are sufficient for a delineation of *C*. *posterocompressus* from *C*. *paralela*.

*C*. *paralela* has originally been described being a punctate species with simple marginal pore canals. Initially, an internal eye-spot has not been mentioned but can be anticipated on the figures in Purper (1979; e.g. Pl. 6, Fig. 12). However, in their re-evaluation of that species Purper & Pinto (1985: 427, 430) assigned that species to “a peculiar lineage of very smooth forms” and recognized an “internal eye protuberance”. The anterior part of the inner lamella is of “intermediate type” (narrower vestibulum but wider fused zone than *C*. *posterocompressus* but with vestibulum and shorter pore canals than *C*. *simplex*; Purper & Pinto 1983). The characters of our material fit best with *C*. *paralela* to which we here assign it.

In core 1AS-32-AM Purper & Pinto (1985) discussed an evolutionary trend starting in the lower part with *C*. *simplex* (no vestibulum, long, bifurcated, anterior marginal pore canals, no eye-spot) and leading to *C*. *posterocompressus* (large vestibulum, short, simple pore canals, prominent eye-spot) up-section, with *C*. *paralela* as an intermediate species in the middle part of the core. Comparably, at core 1AS-10-AM, *C*. *simplex* occurs only in the lower part (AM10/40–39), followed by *C*. *paralela* up-section (AM10/30–15). However, valves with well-developed eye-spots are restricted to AM10/30, which appears to contradict the observation of Pinto & Purper (1985; most prominent eye-spots in the upper part). Possibly, the expression of the eye-spot is rather ecologically controlled than a phylogenetic character.

**Occurrence** (of *C*. *olivencai* sensu Muñoz-Torres *et al.* 1998). Western Amazonia (Brazil, Colombia, Peru), early Middle to early Late Miocene (*C*. *aulakos–C*. *cyrtoma* zone; Muñoz-Torres *et al.* 2006; chronostratigraphic correlation after Wesselingh & Ramos 2010). Up to now only recognized in Brazil (cores CPCAN-III-São Paulo de Olivença and CPCAN-I-Tamanduá; Purper 1979; core 1AS-32-AM; Purper & Pinto 1983, 1985).

***Cyprideis simplex* (Sheppard & Bate, 1980)**

Pl. 4, Figs. 46–50

**Table:** 

*	1980 *Botulocyprideis simplex* sp. nov.—Sheppard & Bate: 104; Pl. 9, Figs. 6, 8–13.
	1983 *Botulocyprideis simplex* Sheppard et Bate emend Purper et Pinto—Purper & Pinto: 116–117; Pl. 2, Figs. 17–27.
	1985 *Botulocyprideis simplex* Sheppard & Bate, 1980—Purper & Pinto: 427, 430; Fig. 2.

**Material.** 6 valves; samples AM10/39–40.

**Dimensions** (total range over all samples). R♀ l = 0.73–0.76 (0.75), h = 0.38–0.40 (0.39; n = 2); L♀? l = 0.77, h = 0.39 (n = 1).

**Remarks.** Only six adult valves were found, which are slightly less elongated in lateral view and somewhat larger than the given synonyms. However, other features enable their attribution to *C*. *simplex* (approximately parallel dorsal and ventral margins; smooth surface; avestibulate; numerous, simple or sometimes bifurcated pore canals; inverse hinge; no internal eye-spot; Sheppard & Bate 1980; Purper & Pinto 1983, 1985). By following Whatley *et al.* (1998), *Botulocyprideis* Sheppard & Bate, 1980 is a junior synonym of *Cyprideis*. For differences to *C*. *paralela* see above.

**Occurrence.** Western Amazonia (Brazil: core 1AS-32-AM, depth: 107–132 m, altitude: -10 to -35 m; Purper & Pinto 1983, 1985; Peru: “Pichua, W Cochaquinàs”; Sheppard & Bate 1980), Middle Miocene (*C*. *caraionae–C*. *minipunctata* zone; according to Wesselingh *et al.* 2006b the outcrops around “Pichua” belong to mollusc zone 7; chronostratigraphic correlation after Wesselingh & Ramos 2010).

#### 4.5.5 “Smooth” species not attributed to subgroups

***Cyprideis multiradiata* (Purper, 1979)**

Figs. 4p–r; Pl. 5, Figs. 1–30

**Table:** 

	1977 Ostracoda A n.g.,n.sp.—Purper: 359; Pl. 1, Figs. 1–8.
*	1979 *Amazonacytheridea multiradiata* Purper, gen. et sp. nov.—Purper: 234–235; Pl. 5, Figs. 1–9.
	1985 *Amazonacytheridea multiradiata* Purper, 1979—Purper & Pinto: 427, 430; Fig. 3.
pars	1998 *Cyprideis olivencai* (Purper, 1979)—Muñoz-Torres *et al.*: 100; Pl. 4, Figs. 7, ?5. [non Pl. 4, Fig. 6]
pars	1998 *Cyprideis olivencai* (Purper, 1979)—Whatley *et al.*: 236; Text-fig. 2 (lowermost row, left drawing); Pl. 2, Figs. 4–5, ?3 [sic]. [non Pl. 2, Figs. 1–2 [sic]]

**Material.** 958 valves; samples AM10/3, 15, 19–30, 40, 42.

**Dimensions** (total range over all samples). R♀ l = 0.65–0.87 (0.77), h = 0.31–0.41 (0.36; n = 119); L♀ l = 0.72–0.89 (0.79), h = 0.36–0.42 (0.38; n = 16); R♂ l = 0.74–0.89 (0.82), h = 0.31–0.39 (0.35; n = 12); L♂ l = 0.74–0.89 (0.82), h = 0.33–0.40 (0.37; n = 11); Rj(A-1) l = 0.63–0.67 (0.65), h = 0.30–0.32 (0.31; n = 4); Lj(A-1) l = 0.62–0.66 (0.64), h = 0.30–0.33 (0.32; n = 6).

**Remarks.** This smooth, asulcate, elongated-ovate species occurs regularly throughout the productive samples of the studied core. Characteristic are: the very wide, anteroventrally additionally enlarged inner lamella with a vestibulum (Figs. 4p–r); the anterior polyfurcated, ventral and posterior simple or bifurcated marginal pore canals as well as the hinge (long, elongated, denticulated anterior and posterior elements; very short, inconspicuously divided, crenulated median element; Purper 1979). More elongated and posteriorly more tapered male valves express sexual dimorphism. While the inner lamella is frequently wider posteroventrally and the anterior vestibulum is usually larger in males, the hinge elements are more strongly developed in female specimens.

**Plate 5:**
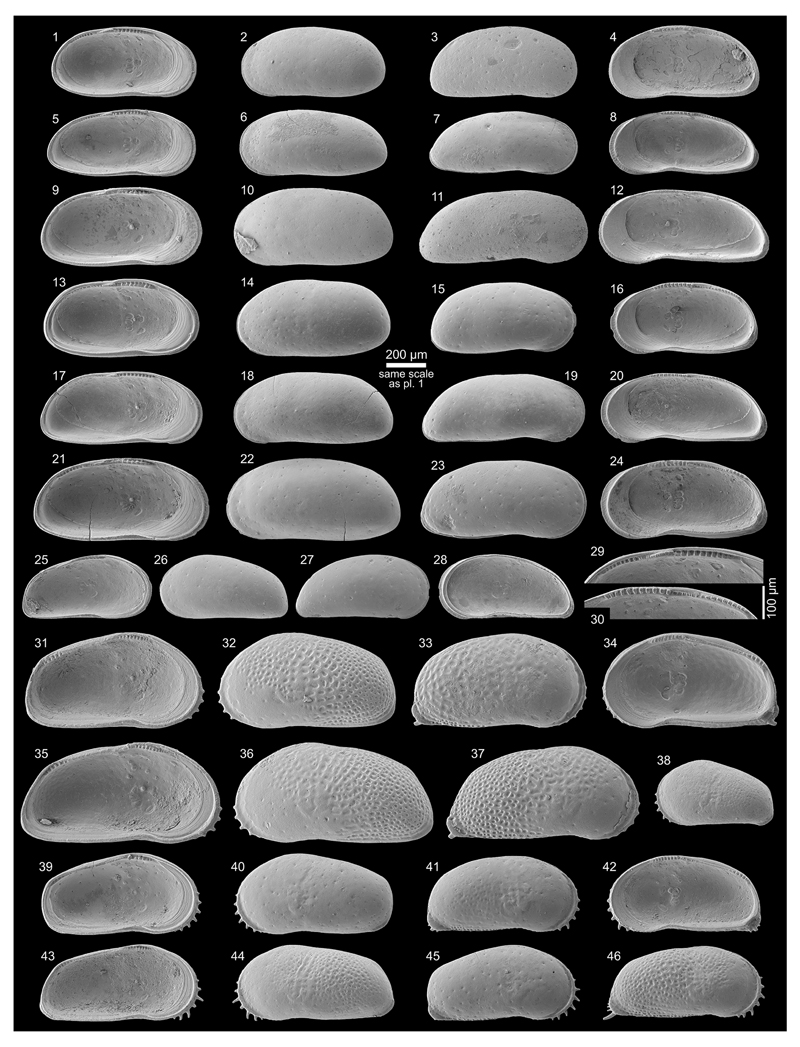
*Cyprideis multiradiata* (Purper, 1979) Figs. 1–30 1–2: L♀, i-e (0.73/0.36; AM10-15_01) 3–4: R♀, e-i (0.76/0.37; AM10-3_91) 5–6: L♂, i-e (0.74/0.33; AM10-15_02) 7–8: R♂, e-i (0.74/0.31; AM10-15_05) 9–10: L♀, i-e (0.81/0.39; AM10-26_02) 11–12: R♂, e-i (0.85/0.36; AM10-26_08) 13–14: L♀, i-e (0.77/0.38; AM10-27_24) 15–16: R♀, e-i (0.73/0.35; AM10-27_26) 17–18: L♂, i-e (0.81/0.36; AM10-27_23) 19–20: R♂, e-i (0.82/0.34; AM10-27_29) 21-22: L♀, i-e (0.87/0.42; AM10-30_24) 23–24: R♀, e-i (0.83/0.38; AM10-42_08) 25–26: Lj, i-e (0.64/0.32; AM10-30_38) 27–28: Rj, e-i (0.66/0.32; AM10-30_39) 29: = detail of Fig. 1; hinge 30: = detail of Fig. 16; hinge *Cyprideis cyrtoma* Muñoz-Torres, Whatley & Van Harten, 1998 Figs. 31–46 31–32: L♀, i-e (0.88/0.47; AM10-3_60) 33–34: R♀, e-i (0.87/0.47; AM10-3_70) 35–36: L♂, i-e (0.99/0.49; AM10-3_61) 37: R♂, e (0.97/0.46; AM10-3_67) 38: Rj, e (0.58/0.33; AM10-23_39) 39–40: L♀, i-e (0.78/0.40; AM10-23_19) 41–42: R♀, e-i (0.75/0.37; AM10-23_29) 43–44: L♂, i-e (0.77/0.37; AM10-23_25); ornamented variant 45: R♀, e (0.73/0.37; AM10-23_32); variant with truncated posterior margin 46: R♂, e (0.76/0.36; AM10-23_16); ornamented variant

Through its occurrence in our core, these diagnostic features remain stable. Evolutionary tendencies as described by Purper & Pinto (1985) have not been observed in our material (e.g. variations of the anteroventral inflection of the inner lamella). However, the outline varies notably (from ovate to very elongated) between samples but without clear, “directed” trend. On plate 5 we illustrate typical (Pl. 5, Figs. 9–10; according to the original description of Purper 1979) as well as extreme morphotypes. Variability of size is shown in figure 5.

Muñoz-Torres *et al.* (1998) and Whatley *et al.* (1998) synonymised *Amazonacytheridea multiradiata* as well as *Pseudoparakrithella paralela* Purper, 1979 and *Botulocyprideis simplex* Sheppard & Bate, 1980 with *Cyprideis olivencai* (Purper, 1979), which in turn became a collective species (see chapters 4.5.3. and 4.5.4.). Due to the constant occurrence of the *multiradiata*-morphotype, which does not show transitions to *C*. *olivencai*, *C*. *paralela* and *C*. *simplex*, *A*. *multiradiata* appears to form a distinct taxon and is revalidated here. According to the generic concept of Whatley *et al.* (1998)—which we follow with reservation—*Amazonacytheridea* Purper, 1979 is a junior synonym of *Cyprideis* and *A. multiradiata* turns into *Cyprideis multiradiata*.

**Occurrence** (of *C*. *olivencai* sensu Muñoz-Torres *et al.* 1998). Western Amazonia (Brazil, Colombia, Peru), early Middle to early Late Miocene (*C*. *aulakos–C*. *cyrtoma* zone; Muñoz-Torres *et al.* 2006; chronostratigraphic correlation after Wesselingh & Ramos 2010). Up to now only recognized in Brazil (cores CPCAN-III-São Paulo de Olivença and CPCAN-I-Tamanduá; Purper 1979; core 1AS-32-AM; Purper & Pinto 1985).

### 4.6 “Ornate” *Cyprideis* group

#### 4.6.1 *cyrtoma* subgroup

**Species.** *C*. *cyrtoma*, *C*. *schedogymnos*; probably *C*. *krsticae* (not among the present material, re-examination pending).

**Characters.** Subrectangular; medium sized; almost smooth to punctate, weakly sulcate; short anteromarginal spines (both valves) restricted to the lower half of anterior margin, some posteroventral denticles or spines only in right valves; moderately wide inner lamella, avestibulate; anterior simple or bifurcated marginal pore canals; generotypic hinge.

***Cyprideis cyrtoma* Muñoz-Torres, Whatley & Van Harten, 1998**

Figs. 6a–b; Pl. 5, Figs. 31–46; Pl. 6, Figs. 1–21, 23

**Table:** 

	1998 *Cyprideis* sp. 1—Whatley *et al.*: 236; Text-fig. 2; Pl. 3, Figs. 6–10.
*	1998 *Cyprideis cyrtoma* sp. nov.—Muñoz-Torres *et al.*: 94–96; Text-fig. 2; Pl. 2, Figs. 15–19.
	2011 *Cyprideis cyrtoma*—Linhares *et al.*: 95, 98; Figs. 3/7–8.

**Material.** 421 valves; samples AM10/3, 16, 22–23, 25, 27, 29–30.

**Dimensions** (total range over all samples). R♀ l = 0.68–0.87 (0.75), h = 0.34–0.47 (0.39; n = 77); L♀ l = 0.73–0.95 (0.82), h = 0.38–0.52 (0.43; n = 12); R♂ l = 0.74–0.97 (0.83), h = 0.35–0.46 (0.39; n = 10); L♂ l = 0.77–0.99 (0.87), h = 0.37–0.49 (0.42; n = 7); Rj(A-1) l = 0.57–0.66 (0.61), h = 0.32–0.38 (0.35; n = 5); Lj(A-1) l = 0.58–0.65 (0.61), h = 0.33–0.35 (0.34; n = 4).

**Remarks.** *C*. *cyrtoma* is characterised by its “parallel” anterodorsal and anteroventral margins, which cause a well-expressed oral concavity in both valves. Typically, the anterior valve surface is almost smooth, while the posterior proportion (but not peripherally) is ornamented with puncta (Muñoz-Torres *et al.* 1998). The differences to the closely related *C*. *schedogymnos* are discussed in the remarks for this species (see below).

Within core 1AS-10-AM considerable variability is observed in size (Fig. 5), outline, and especially in the degree of ornamentation: e.g. the rather large valves of sample AM10/30 are almost smooth (Pl. 6, Figs. 9–16); the small individuals in AM10/27 are coarsely punctated (also in front of the sulcus; Pl. 6. Figs. 1–8); in AM10/23 small, “smooth” and punctated valves as well as specimens with truncated and rounded posterior margin co-occur (Pl. 5, Figs. 39–46); the very large valves in AM10/3 are coarsely punctated (Pl. 5, Figs. 31–37).

Well ornamented specimens of *C*. *cyrtoma* come close to the herein described *C*. aff. *graciosa* (see chapter 4.6.1.) but have a more horizontally aligned posteroventral spine, sitting on a more prominent flange (right valves); the shorter anterior spines are restricted to the lower half of the anterior margin; the ornament along the anterior margin is smoother, and they are slightly smaller.

Further, *C*. *longispina* from the Eirunepé area (Gross *et al.* 2013) is similar but has a more elongated outline with an additional extension of the posteroventral flange (right valve) above the posteroventral projection, carrying regularly only one spine and no further denticles. The anterior proportion is more strongly ornamented.

**Occurrence.** Western Amazonia (Colombia), latest Middle to early Late Miocene (*C*. *obliquosulcata–C*. *cyrtoma* zone; Muñoz-Torres *et al.* 2006; chronostratigraphic correlation after Wesselingh & Ramos 2010).

***Cyprideis schedogymnos* Muñoz-Torres, Whatley & Van Harten, 1998**

Fig. 6c; Pl. 6, Figs. 22, 24–32

**Table:** 

*	1998 *Cyprideis schedogymnos* sp. nov.—Muñoz-Torres *et al.*: 100; Text-fig. 2; Pl. 4, Figs. 11–15.

**Material.** 62 valves; samples AM10/41–44.

**Dimensions** (total range over all samples). R♀ l = 0.76–0.82 (0.79), h = 0.39–0.40 (0.40; n = 4); L♀ l = 0.78–0.80 (0.79), h = 0.42–0.43 (0.43; n = 3); R♂ l = 0.87, h = 0.42 (n = 1); L♂ l = 0.88, h = 0.44 (n = 1).

**Remarks.** *C*. *schedogymnos* occurs only in the lowermost productive samples in the present core. It largely resembles *C*. *cyrtoma*, from which it differs by the lack of the “downturned” anterior margin causing a prominent ventral concavity in both valves of *C*. *cyrtoma*, which is almost missing in *C*. *schedogymnos* (Muñoz-Torres *et al.* 1998). *C*. *cyrtoma* has a more extended posteroventral flange with up to five denticles and usually one long spine in the posteroventral corner of right valves (Pl. 6, Fig. 23). *C*. *schedogymnos* lacks such a main spine and the denticles are smaller (Pl. 6, Fig. 24). *C*. *schedogymnos* is a smooth species except some posteroperipheral puncta and “plications” (a row of thick walled fossae) behind the strong anteromarginal rim (Pl. 6, Fig. 22; Muñoz-Torres *et al.* 1998). *C*. *cyrtoma* misses these plications; instead, it has a smooth groove following the course of the anterior border (Pl. 6, Fig. 21). Furthermore, *C. cyrtoma* has a weak, sinuous sulcus, whereas *C*. *schedogymnos* is almost asulcate.

Typically, the surface of *C*. *cyrtoma* is smooth in front of the sulcus and punctate behind it. However, considerable variability of ornamentation is observed, ranging from almost smooth to punctate (except the area behind the anterior margin) specimens, which weakens this diagnostic character (see above).

In the phylogenetic model of Muñoz-Torres *et al.* (2006) *C*. *schedogymnos* and *C*. *cyrtoma* are placed in two different lineages (*C*. *cyrtoma*: “ornate”, *C*. *schedogymnos*: “smooth lineage”). In contrast, we suggest that both are much closer related and belong to the same lineage (Fig. 3a).

**Occurrence.** Western Amazonia (Brazil (this study), Peru), Middle Miocene (*C*. *caraionae–C*. *minipunctata* zone; Muñoz-Torres *et al.* 2006; chronostratigraphic correlation after Wesselingh & Ramos 2010).

#### 4.6.2 *graciosa* subgroup

**Species.** *C*. *graciosa*, *C*. *reticulopunctata*, *C*. *minipunctata*, *C*. *curucae*, *C*. *longispina* (the latter not among the present material); possibly *C*. *anterospinosa*, *C*. *marginuspinosa*, *C*. *retrobispinosa* (all not among the present material and re-examination pending).

**Characters.** Subrectangular, subtriangular or subovate; medium to large sized; punctated to reticulated, sulcate; with spines/denticles along the entire or the two lower thirds of the anterior margin (both valves) and posteroventral spines in right valves (except *C*. *retrobispinosa*; due to reversed overlap in left valves), some with posteroventral spines in both valves (*C*. *graciosa*, *C*. *reticulopunctata*); essentially moderately wide inner lamella, avestibulate; anterior simple or few bifurcated marginal pore canals; generotypic hinge (*C*. *retrobispinosa* with inverse hinge).

***Cyprideis* aff. *graciosa* (Purper, 1979)**

Fig. 6d; Pl. 6, Figs. 33–34; Pl. 7, Figs. 1–20

**Table:** 

?		1977 *Cytheridea* sp.nov. D—Purper: 363; Pl. 3, Figs. 5–6.
?	*	1979 *Cytheridea graciosa* Purper, sp. nov.—Purper: 229–230; Pl. 3, Figs. 1–9.
?		1991 *Cytheridea graciosa* Purper, 1979—Purper & Ornellas: 26–28; Pl. 1, Figs. 10–15.
?		1998 *Cyprideis graciosa* (Purper, 1979)—Muñoz-Torres *et al.*: 96; Pl. 3, Figs. 1–3.
?		1998 *Cyprideis graciosa* (Purper, 1979)—Whatley *et al.*: 234; Text-fig. 2; Pl. 1, Figs. 11–15.
?		2006 *Cyprideis graciosa* Purper, 1979—Ramos: 92; Figs. 7d–h.
?		2009 *Cyprideis graciosa* Purper, 1979—Ramos *et al.*: 114; Fig. 289-I.
?		2010 *Cyprideis graciosa* Purper, 1979—Wesselingh & Ramos: 308; Figs. 18.5e–f.
?		2011 *Cyprideis graciosa*—Linhares *et al.*: 96; Figs. 3/9–10.
?		2013 *Cyprideis graciosa* (Purper, 1979)—Gross *et al.*: 225; Pl. 4, Figs. 1–17.

**Material.** 319 valves; samples AM10/15–16, 19, 22–30.

**Dimensions** (total range over all samples). R♀ l = 0.76–0.91 (0.82), h = 0.40–0.49 (0.44; n = 8); L♀ l = 0.72–0.92 (0.83), h = 0.38–0.49 (0.45; n = 10); R♂ l = 0.80–0.95 (0.89), h = 0.39–0.48 (0.43; n = 4); L♂ l = 0.83–0.98 (0.91), h = 0.42–0.49 (0.45; n = 6); Rj(A-1) l = 0.63–0.66 (0.65), h = 0.37–0.39 (0.38; n = 3); Lj(A-1) l = 0.64–0.70 (0.68), h = 0.38–0.40 (0.39; n = 3); Rj(A-2) l = 0.55–0.56 (0.56), h = 0.30–0.31 (0.30; n = 3); Lj(A-2) l = 0.52, h = 0.27 (n = 1).

**Remarks.** *C*. *graciosa* is a moderately large, subrectangular to subtriangular form with a pitted ornament, with about eight strong and widely spaced spines along the entire anterior margin and about four posteroventral spines of which the lowermost one is the most developed. The anteroventral and anterocentral surface ornament tends to be reduced, forming a low reticulum (sometimes with weakly developed, secondary fine puncta) or is almost smooth (Purper 1979; Purper & Ornellas 1991; Ramos 2006; Wesselingh & Ramos 2010; Gross *et al.* 2013).

The present material may range within the variability of *C*. *graciosa* in a wider sense. However, posteroventral spines are not developed in left valves of the present material, which are a characteristic feature according to Purper (1979) and Purper & Ornellas (1991). Equally, posteroventral spines are not visible in the figured specimens of Muñoz-Torres *et al.* (1998), Whatley *et al.* (1998) and Linhares *et al.* (2011). However, such spines are mentioned in the descriptions provided by the former authors. As discussed by Gross *et al.* (2013), it remains unclear if solely the occurrence or absence of posteroventral spines in left valves is species diagnostic or not. Provisionally, we attribute our specimens to *C*. aff. *graciosa*.

Similar species are *Cyprideis anterospinosa* Purper & Ornellas, 1991 and *Cyprideis marginuspinosa* (Purper & Ornellas, 1991). The former diverges by its more rounded dorsal margin, the less concave ventral margin, a punctate surface, small posteroventral spines in right valves and the anteromarginal spines are restricted to the anteroventral margin (Purper & Ornellas 1991).

*C*. *marginuspinosa* is punctate, has small posteroventral spines in right valves (but note the well-developed spine of the specimen on Pl. 2, Fig. 5 in Purper & Ornellas 1991), a more rounded posterocardinal angle and anteromarginal spines that only slightly exceed half of the valves’ height (Purper & Ornellas 1991).

Somewhat comparable is *C*. *longispina*, which differs from *C*. *graciosa* by: e.g. its significantly extended posteroventral flange in right valves, which holds usually one more horizontally directed posteroventral spine; a posteroventral spine in left valves is never developed; its anterior spines are restricted to the lower half of the anterior margin; and the ornament is reduced in the anteroventral area only (Gross *et al.* 2013).

**Occurrence** (of *C*. *graciosa*). Western Amazonia (Brazil, Colombia, Peru), latest Middle to early Late Miocene (*C*. *obliquosulcata–C*. *cyrtoma* zone; Muñoz-Torres *et al.* 2006; chronostratigraphic correlation after Wesselingh & Ramos 2010).

***Cyprideis reticulopunctata* (Purper, 1979)**

Pl. 7, Figs. 21–31

**Table:** 

	1977 Cytheridea sp.nov. B—Purper: 361; Pl. 2, Figs. 7–13.
*	1979 *Cytheridea reticulopunctata* Purper, sp. nov.—Purper: 227–228; Pl. 2, Figs. 1–10.

**Material.** 101 valves; sample AM10/3.

**Dimensions** (total range over all samples). R♀ l = 0.87–0.93 (0.89), h = 0.48–0.52 (0.49; n = 5); L♀ l = 0.89–0.93 (0.91), h = 0.49–0.54 (0.52; n = 4); R♂ l = 0.96–1.00 (0.99), h = 0.51–0.53 (0.52; n = 3); L♂ l = 0.95–1.06 (1.00), h = 0.48–0.56 (0.52; n = 5); ?Lj(A-1) l = 0.70–0.79 (0.75), h = 0.38–0.49 (0.44; n = 2).

**Remarks.** This subovate–subrectangular species is characterised by its ornamentation with a low, internally punctated reticulum (“compose puncture forming broad reticulum”; Purper 1979: 227). Along the entire anterior margin, little marginal spines are developed. One prominent posteroventral spine is developed in right and left valves. Right valves carry 3–4 additional denticles above this main spine (Purper 1979).

The current specimens perfectly coincide with the description of Purper (1979), which, however, remains the only evidence of that species up to now. In core 1AS-10-AM, it occurs only in the uppermost productive sample (AM10/3). Here we apply the generic concept of Whatley *et al.* (1998) and transfer this species to the genus *Cyprideis*.

*C*. *reticulopunctata* is extremely similar to the slightly smaller *C*. *graciosa*, which has a pitted ornament with the tendency to become reduced anteroventrally and anterocentrally. *C.* aff. *graciosa* (material herein, see above) additionally lacks posteroventral spines in left valves and has more prominent anteromarginal spines.

*C*. *longispina* is very close to *C*. *reticulopunctata* but the former is more elongated, typically with a very extended flange posteroventrally (right valves), and with an overall finer (punctate) ornament. Anterior spines are restricted to the lower half of the anterior margin and posteroventral spines are never developed in left valves (Purper 1979; Muñoz-Torres *et al.* 1998; Gross *et al.* 2013).

**Occurrence.** Western Amazonia (Brazil), latest Middle to early Late Miocene (*C*. *obliquosulcata–C*. *cyrtoma* zone; this study; chronostratigraphic correlation after Wesselingh & Ramos 2010). Up to now only known from core CPCAN-III-São Paulo de Olivença (depth: 31.52–32.62 m, altitude: ~21 m; Purper 1979).

***Cyprideis minipunctata* (Purper & Ornellas, 1991)**

Fig. 6e, Pl. 7, Figs. 32–35; Pl. 8, Figs. 1–9

**Table:** 

?pars	1980 *Cyprideis purperi purperi* subsp. nov.—Sheppard & Bate: 99–101; Text-fig. 2; Pl. 7, Figs. 1–11; Pl. 8, Figs. 1–2. [non Pl. 7, Fig. 13 and probably non Fig. 12]
*	1991 *Cytheridea minipunctata* Purper & Ornellas, sp. nov.—Purper & Ornellas: 28–30; Pl. 2, Figs. 7–12.
	1998 *Cyprideis minipunctata* (Purper & Ornellas, 1991)—Muñoz-Torres *et al.*: 98; Pl. 3, Figs. 18–20.
	1998 *Cyprideis minipunctata* (Purper & Ornellas, 1991)—Whatley *et al.*: 235–236; Pl. 2, Figs. 11–15 [sic].

**Material.** 103 valves; samples AM10/31, 39–40, 42.

**Dimensions** (total range over all samples). R♀ l = 0.80–0.85 (0.82), h = 0.43–0.45 (0.44; n = 5); L♀ l = 0.80–0.85 (0.83), h = 0.42–0.47 (0.45; n = 3); R♂ l = 0.91–0.96 (0.94), h = 0.45–0.47 (0.46; n = 3); L♂ l = 0.93–0.98 (0.96), h = 0.44–0.50 (0.47; n = 4).

**Remarks.** Our subovate–subrectangular, finely punctate specimens match well with the material of Purper & Ornellas (1991), Muñoz-Torres *et al.* (1998) and Whatley *et al.* (1998).

In part (except the specimens which clearly (Pl. 7, Fig. 13) or probably (Pl. 7, Fig. 12) belong to *C*. *sulcosigmoidalis*) *C*. *purperi purperi* of Sheppard & Bate (1980) is very similar to *C*. *minipunctata*. As far as the available descriptions and figurations of both taxa enable a comparison, the main difference is the “smooth anterior marginal zone” (Sheppard & Bate 1980: 100) in *C*. *purperi purperi*, whereas this area is covered by “shallow oval pits” (Muñoz-Torres *et al.* 1998: 98) in *C*. *minipunctata*. Considering the variability herein observed and also mentioned by Muñoz-Torres *et al.* (1998: 98; some “have coarser puncta”), it is possible that *C*. *minipunctata* is a junior synonym of *C*. *purperi purperi* (compare variability of ornament in sample AM10/39; Pl. 7, Figs. 33, 35). However, this claim requires a reinvestigation of the material of Sheppard & Bate (1980).

*C*. *minipunctata* is very close to *C*. *longispina*, which is more coarsely punctated, anteroperipherally punctated or almost smooth anteroventrally, and has a weak “double” sulcus as well as an extended posteroventral flange, the latter carrying a longer posteroventral main spine on right valves (Purper 1979; Muñoz-Torres *et al.* 1998; Gross *et al.* 2013).

**Occurrence.** Western Amazonia (Brazil, Colombia, Peru), Middle Miocene (*C*. *aulakos–C*. *minipunctata* zone; Muñoz-Torres *et al.* 2006; chronostratigraphic correlation after Wesselingh & Ramos 2010).

***Cyprideis curucae* nom. nov.**

Fig. 6f; Pl. 8, Figs. 10–33

**Table:** 

*	1983 *Sohnicythere tuberculata* Purper et Pinto gen. et sp. nov.—Purper & Pinto: 119; Pl. 3, Figs. 1-11.
	1998 *Cyprideis* sp. 1—Muñoz-Torres *et al.*: 102; Pl. 5, Figs. 1–2.
	1998 *Cyprideis* sp. 2—Muñoz-Torres *et al.*: 102; Pl. 5, Figs. 3–4 [sic].
	1998 *Cyprideis* sp. 2—Whatley *et al.*: 237; Pl. 3, Figs. 11–13.
	1998 *Cyprideis* sp. 3—Whatley *et al.*: 237; Pl. 3, Figs. 14–15.
	2011 *Cyprideis* sp. 1—Linhares *et al.*: 95, 98; Figs. 4/7–8.

**Material. 72 valves; samples AM10/3, 19, 22–23, 27–28, 30.**

**Dimensions** (total range over all samples). R♀ l = 0.78–0.86 (0.83), h = 0.42–0.44 (0.43; n = 5); L♀ l = 0.85–0.89 (0.87), h = 0.46–0.47 (0.47; n = 2); R♂ l = 0.84–0.97 (0.92), h = 0.40–0.46 (0.43; n = 3); L♂ l = 0.87–0.99 (0.93), h = 0.43–0.48 (0.46; n = 4).

**Remarks.** The specimens of sample AM10/30 (Pl. 8, Figs. 22–29) coincide with the original description of this species (Purper & Pinto 1983) as well as with *Cyprideis* sp. 2 and *Cyprideis* sp. 3 of Muñoz-Torres *et al.* (1998), and Whatley *et al.* (1998), respectively. It is a subtriangular to subrectangular, reticulated to coarsely punctated *Cyprideis* with regularly spaced tubercles, one very long (mostly broken) posteroventral spine above which three shorter spines originate (only in right valves; Pl. 8, Figs. 30–31) and a comparably wide marginal zone (Purper & Pinto 1983; Muñoz-Torres *et al.* 1998).

In samples AM10/27 and 19—where further well-preserved specimens are available—tubercles are less distinct, which cause the punctate ornamentation as well as the sulcus to come to the fore (Pl. 8, Figs. 10–21). Nevertheless, tubercles, which correspond to normal pore openings, are still observable (compare Pl. 8, Figs. 32 and 33) while other valve characters remain constant (compare for example Pl. 8, Figs. 18–19 with Figs. 26–27). Thus, the expression of tubercles/pore conuli as well as the reticulate/punctate ornamentation is considered to be ecologically controlled (compare i.e., tubercles in ilyocypridids; Yang *et al.* 2002; Gross *et al.* 2013).

Based on this observation the species *Cyprideis* sp. 1 of Muñoz-Torres *et al.* (1998 = *Cyprideis* sp. 2 of Whatley *et al.* 1998) can be included into this species (note that some tubercles are still visible on posterior (right valve) and anteroventral (left valve) parts of the shells on Figs. 11 and 13 (Pl. 3) of Whatley *et al.* 1998). Similarly, *Cyprideis* sp. 1 of Linhares *et al.* (2011) represents the weaker tuberculated variant of this taxon.

If tubercles are not very prominent, this species resembles *C*. aff. *graciosa* (see above; compare e.g. Pl. 6, Figs. 41–42 with Pl. 8, Figs. 18–19). However, *C. curucae* is well differentiated by: i) a wider inner lamella, ii) a more distal course of the selvage, iii) a restriction of anterior spines to the lower two-thirds of the anterior margin, iv) the lack of a smooth area at the anterior valve part, and, to a lesser degree, v) a thinner anterior marginal ridge and weaker developed hinge elements.

Originally, the species under discussion here has been named *Sohnicythere tuberculata* by Purper & Pinto (1983). However, Whatley *et al.* (1998) consider the genus *Sohnicythere* Purper & Pinto, 1983 as a junior synonym of *Cyprideis*. By applying this generic concept, *S. tuberculata* turns into “*Cyprideis tuberculata* (Purper & Pinto, 1983)” (see Muñoz-Torres *et al.* 1998: 102), which is a secondary homonym (ICZN articles 53.3, 57.3) of the Central European, Late Miocene *Cyprideis tuberculata* (Méhes, 1908). For this reason the substitute name *Cyprideis curucae* nom. nov. is introduced here (ICZN article 60.3; derivation of name: Rio Curuçá, where well 1AS-32-AM was drilled and the type material comes from; Purper & Pinto 1983, 1985).

**Occurrence** (of the synonyms *Cyprideis* sp. 1–2 of Muñoz-Torres *et al.* 1998). Western Amazonia (Colombia), latest Middle to early Late Miocene (*C*. *obliquosulcata–C*. *cyrtoma* zone; Muñoz-Torres *et al.* 2006; chronostratigraphic correlation after Wesselingh & Ramos 2010).

#### 4.6.3 *pebasae* subgroup

**Species.** *C*. *pebasae*, *C*. *munoztorresi*, *C*. *ituiae*, *C*. *matorae*; possibly *C*. *purperi colombiaensis* but status unclear and re-examination pending.

**Characters.** Subrectangular to subtriangular; moderately small to medium sized; coarsely punctated to reticulated, asulcate to prominent sulcus; with antero- (both valves) and posteroventral spines (only right valves); moderately wide inner lamella, avestibulate; anterior essentially simple marginal pore canals; generotypic hinge.

***Cyprideis pebasae* (Purper, 1979)**

Fig. 6g; Pl. 8, Figs. 34–43; Pl. 9, Figs. 1–24; Pl. 10, Figs. 45, 47, 51

**Table:** 

	1977 *Cytheridea* sp.nov. C—Purper: 363; Pl. 3, Figs. 1–4.
*	1979 *Cytheridea pebasae* Purper, sp. nov.—Purper: 228–229; Pl. 2, Figs. 11–23.
	1998 *Cyprideis lacrimata* sp. nov.—Muñoz-Torres *et al.*: 96; Text-fig. 2; Pl. 3, Figs. 7–11.
non	1998 *Cyprideis pebasae* (Purper, 1979)—Muñoz-Torres *et al.*: 100; Pl. 4, Figs. 8–10.
non	1998 *Cyprideis pebasae* (Purper, 1979)—Whatley *et al.*: 236; Text-fig. 2; Pl. 2, Figs. 16–20.
pars	2006 *Cyprideis pebasae* (Purper, 1979) Whatley *et al.*, 1998 emend.—Ramos: 90–91; Figs. 6i–y. [non Figs. 6e–h]
	2009 *Cyprideis pebasae* (Purper, 1979)—Ramos *et al.*: 114; Figs. 295–298-I.
	2010 *Cyprideis pebasae* (Purper, 1979)—Wesselingh & Ramos: 308; Figs. 18.5c–d.
	2011 *Cyprideis lacrimata*—Linhares *et al.*: 98; Fig. 3/12.
	2011 *Cyprideis pebasae*—Linhares *et al.*: 95, 98; Figs. 4/3–4.
	2013 *Cyprideis pebasae* (Purper, 1979)—Gross *et al.*: 225–227; Pl. 5, Figs. 1–17.

**Material.** 66 valves; samples AM10/3, 19, 23, 27, 42.

**Dimensions** (total range over all samples). R♀ l = 0.64–0.80 (0.71), h = 0.34–0.40 (0.37; n = 7); L♀ l = 0.67–0.86 (0.73), h = 0.36–0.45 (0.40; n = 8); R♂ l = 0.79–0.93 (0.86), h = 0.37–0.41 (0.39; n = 2); L♂ l = 0.71–0.91 (0.82), h = 0.34–0.43 (0.40; n = 4); Rj(A-1) l = 0.43–0.62 (0.54), h = 0.28–0.35 (0.33; n = 4); Lj(A-1) l = 0.55–0.60 (0.58), h = 0.34–0.37 (0.35; n = 4).

**Remarks.** This subrectangular–subtrapezoidal, coarsely punctate, asulcate species is similar to *C*. *munoztorresi* (see below and Gross *et al.* 2013) from which it differs by its widely spaced (“about seven”; Purper 1979) anteromarginal spines (compare for example Pl. 10, Figs. 47 and 48), the robust anteromarginal and posteroventral flange as well as the selvage runs more distally (right valves). *C*. *munoztorresi* exhibits up to eight, almost uniform posteroventral denticles (e.g. Pl. 10, Fig. 52), whereas they are only inconspicuously developed in *C*. *pebasae* (occasionally the lowermost develops into a short spine; Pl. 10, Fig. 51; compare i.e., Pl. 3, Fig. 11 in Muñoz-Torres *et al.* 1998). The inter- and intra-sample variability (mainly size, outline, ornament) of *C*. *pebasae* is shown on plates 8–9.

*C*. *lacrimata* of Muñoz-Torres *et al.* (1998) as well as *C*. *pebasae* with a “well pronounced anterior and ventroposterior marginal border” and with a “coarsely spinose” flange of Ramos (2006; compare Linhares *et al.* 2011) are synonyms of *C*. *pebasae* (see below and Gross *et al.* 2013).

**Plate 7:**
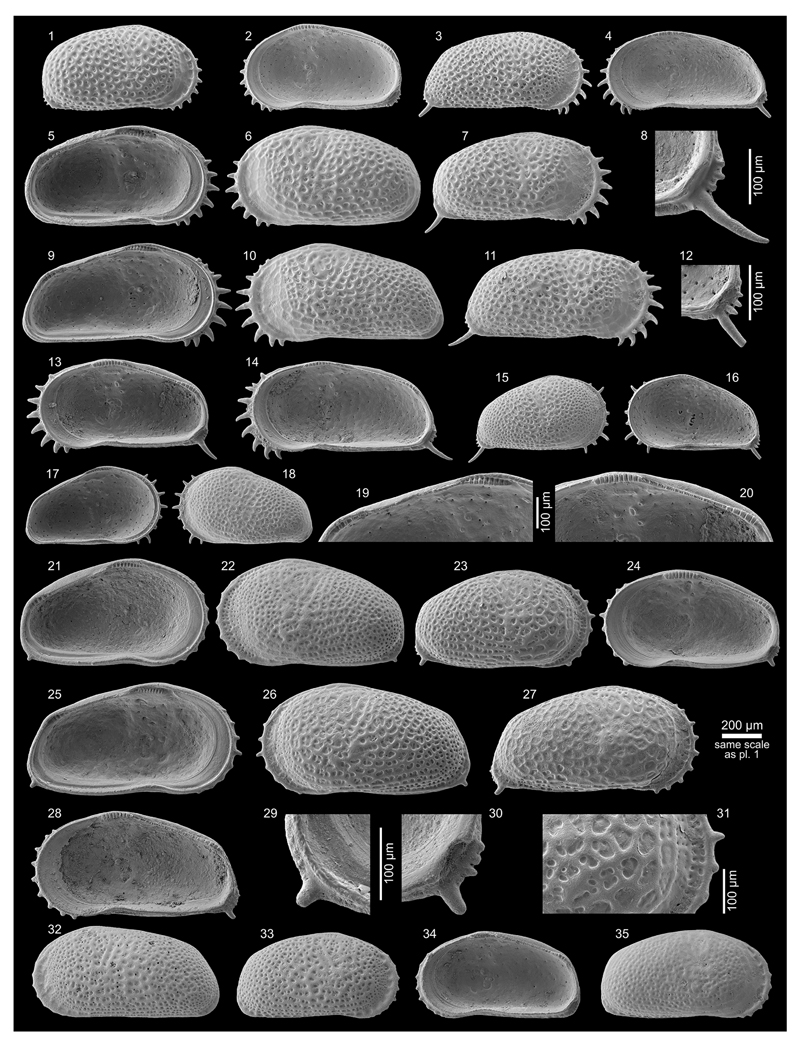
*Cyprideis* aff. *graciosa* (Purper, 1979) Figs. 1–20 1–2: R♀, e-i (0.78/0.42; AM10-27_12) 3–4: R♂, e-i (0.85/0.40; AM10-27_03) 5–6: L♀, i-e (0.92/0.49; AM10-30_61) 7: R♀, e (0.84/0.44; AM10-30_67) 8: = detail of Fig. 14; posteroventral part 9–10: L♂, i-e (0.98/0.49; AM10-30_62) 11: R♂, e (= Fig. 8; 0.94/0.44; AM10-30_69) 12: = detail of Fig. 16; posteroventral part 13: = Fig. 7, i 14: = Fig. 11, i 15–16: Rj, e-i (= Fig. 12; 0.65/0.37; AM10-30_70) 17–18: Lj, i-e (0.70/0.38; AM10-30_65) 19: = detail of Fig. 9; hinge 20: = detail of Fig. 13; hinge *Cyprideis reticulopunctata* (Purper, 1979) Figs. 21–31 21–22: L♀, i-e (0.93/0.53; AM10-3_38) 23–24: R♀, e-i (0.89/0.49; AM10-3_49) 25–26: L♂, i-e (1.06/0.56; AM10-3_40) 27–28: R♂, e-i (1.00/0.53; AM10-3_50) 29: = detail of Fig. 25; posteroventral part 30: = detail of Fig. 24; posteroventral part 31: = detail of Fig. 23; anterior ornamentation pattern *Cyprideis minipunctata* (Purper & Ornellas, 1991) Figs. 32–35 32: L♂, e (0.96/0.46; AM10-39_06) 33–34: R♀, e-i (0.81/0.43; AM10-39_10) 35: R♀, e (0.85/0.44; AM10-39_12)

**Plate 8:**
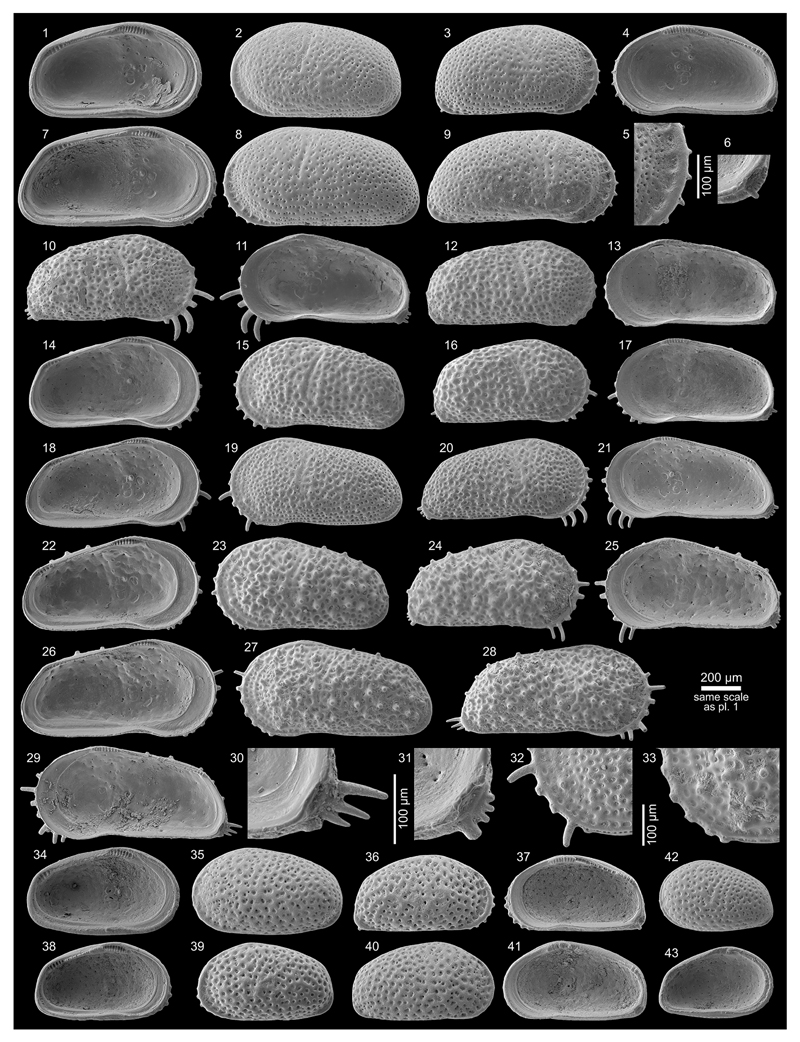
*Cyprideis minipunctata* (Purper & Ornellas, 1991) Figs. 1–9 1–2: L♀, i-e (0.85/0.47; AM10-42_12) 3–4: R♀, e-i (0.83/0.45; AM10-42_17) 5: = detail of Fig. 3; anterior part 6: = detail of Fig. 4; posteroventral part 7–8: L♂, i-e (0.98/0.50; AM10-42_11) 9: R♂, e (0.96/0.47; AM10-42_16) *Cyprideis curucae* nom. nov. Figs. 10–33 10–11: R♀, e-i (0.85/0.44; AM10-19_12) 12–13: R♀, e-i (0.84/0.42; AM10-19_13) 14–15: L♀, i-e (0.85/0.46; AM10-27_68) 16–17: R♀, e-i (0.78/0.42; AM10-27_69) 18–19: L♂, i-e (0.87/0.44; AM10-27_05) 20–21: R♂, e-i (0.84/0.40; AM10-27_04) 22–23: L♀, i-e (0.89/0.47; AM10-30_03) 24–25: R♀, e-i (0.86/0.44; AM10-30_06) 26–27: L♂, i-e (0.98/0.48; AM10-30_01) 28–29: R♂, e-i (0.97/0.46; AM10-30_04) 30: = detail of Fig. 29; posteroventral part 31: = detail of Fig. 25; posteroventral part 32: = detail of Fig. 19; anteroventral part 33: = detail of Fig. 27; anteroventral part *Cyprideis pebasae* (Purper, 1979) Figs. 34–43 34–35: L♀, i-e (0.73/0.40; AM10-3_23) 36–37: R♀, e-i (0.65/0.35; AM10-3_30) 38–39: L♀, i-e (0.67/0.37; AM10-3_25) 40–41: R♀, e-i (0.70/0.38; AM10-3_26) 42–43: Lj, e-i (0.55/0.34; AM10-3_32)

**Plate 9:**
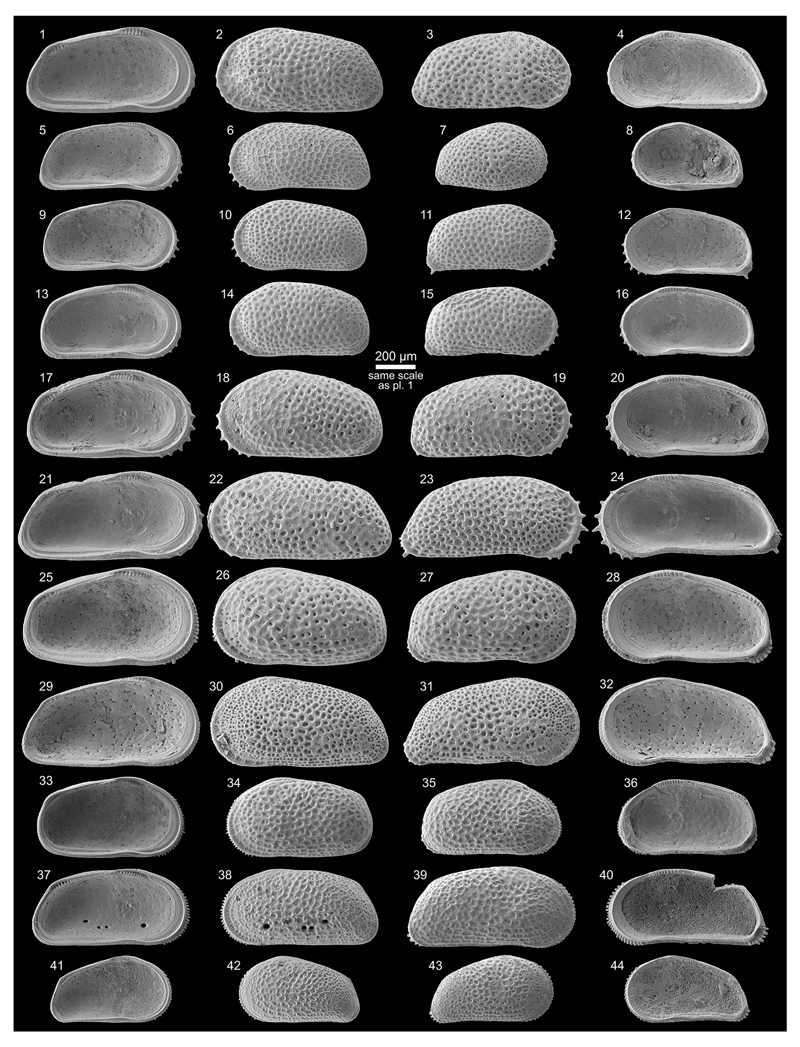
*Cyprideis pebasae* (Purper, 1979) Figs. 1–24 1–2: L♂, i-e (0.84/0.43; AM10-3_96) 3–4: R♂, e-i (0.79/0.37; AM10-3_29) 5–6: L♂, i-e (0.71/0.34; AM10-19_08) 7–8: Rj, e-i (0.55/0.33; AM10-3_35) 9–10: L♀, i-e (0.67/0.36; AM10-23_102) 11–12: R♀, e-i (0.64/0.34; AM10-23_103) 13–14: L♀, i-e (0.70/0.37; AM10-27_70) 15–16: R♀, e-i (0.67/0.34; AM10-27_71) 17–18: L♀, i-e (0.86/0.45; AM10-42_09) 19–20: R♀, e-i (0.80/0.40; AM10-42_14) 21–22: L♂, i-e (0.91/0.43; AM10-42_10) 23–24: R♂, e-i (0.93/0.41; AM10-42_18) *Cyprideis munoztorresi* nom. nov. Figs. 25–44 25–26: L♀, i-e (0.86/0.48; AM10-3_20) 27–28: R♀, e-i (0.83/0.45; AM10-3_19) 29–30: L♂, i-e (0.89/0.45; AM10-3_16) 31–32: R♂, e-i (0.87/0.43; AM10-3_18) 33–34: L♀, i-e (0.73/0.40; AM10-7_90) 35–36: R♀, e-i (0.69/0.37; AM10-7_32) 37–38: L♂, i-e (0.78/0.38; AM10-7_28) 39: R♂, e (0.84/0.41; AM10-7_92) 40: R♂, i (0.80/0.37; AM10-7_30) 41–42: Lj, i-e (0.60/0.33; AM10-7_29) 43–44: Rj, e-i (0.62/0.34; AM10-7_93)

*Cyprideis purperi purperi* Sheppard & Bate, 1980 and *Cyprideis purperi colombiaensis* Sheppard & Bate, 1980, which are placed in the synonymy of *C*. *pebasae* by Ramos (2006), are sulcate *Cyprideis* taxa and display a finer ornament (Sheppard & Bate 1980; Gross *et al.* 2013). Moreover, at least the specimen on plate 7, Fig. 13 (and probably Fig. 12) of Sheppard & Bate (1980) clearly belongs to *C*. *sulcosigmoidalis* (Purper & Pinto 1985).

**Occurrence** (of *C*. *lacrimata* sensu Muñoz-Torres *et al.* 1998). Western Amazonia (Brazil, Colombia, Peru), late Middle to early Late Miocene (*C*. *caraionae–C*. *cyrtoma* zone; Muñoz-Torres *et al.* 2006; chronostratigraphic correlation after Wesselingh & Ramos 2010).

***Cyprideis munoztorresi* nom. nov.**

Figs. 6h–i; Pl. 9, Figs. 25–44; Pl. 10, Figs. 1–44, 46, 48–50, 52

**Table:** 

non	1979 *Cytheridea pebasae* Purper, sp. nov.—Purper: 228–229; Pl. 2, Figs. 11–23.
	1979 *Hulingsina*? sp.—Purper: 239–240; Pl. 7, Figs. 1–5.
non	1998 *Cyprideis lacrimata* sp. nov.—Muñoz-Torres *et al.*: 96; Text-fig. 2; Pl. 3, Figs. 7–11.
	1998 *Cyprideis pebasae* (Purper, 1979)—Muñoz-Torres *et al.*: 100; Pl. 4, Figs. 8–10.
	1998 *Cyprideis pebasae* (Purper, 1979)—Whatley *et al.*: 236; Text-fig. 2; Pl. 2, Figs. 16–20.
pars	2006 *Cyprideis pebasae* (Purper, 1979) Whatley *et al.*, 1998 emend.—Ramos: 90–91; Figs. 6e–h. [non Figs. 6i–y]
	2006 *Cyprideis lacrimata* Muñoz-Torres *et al.*, 1998—Ramos: 92; Figs. 7a–c.
	2009 *Cyprideis lacrimata* Muñoz-Torres *et al.*, 1998—Ramos et al.: 114; Fig. 290-I.
	2010 *Cyprideis lacrimata* Muñoz-Torres *et al.*, 1998—Wesselingh and Ramos: 308; Figs. 18.5g–h.
?	2011 *Cyprideis* sp. 4—Linhares *et al.*: 97; Fig. 4/12.
	2013 *Cyprideis* aff. *pebasae* (Purper, 1979)—Gross *et al.*: 227; Pl. 5, Figs. 18–34.

**Material.** 300 valves; samples AM10/3, 6–7, 15, 22–25, 27–30.

**Dimensions** (total range over all samples). R♀ l = 0.60–0.83 (0.69), h = 0.31–0.45 (0.37; n = 59); L♀ l = 0.64–0.86 (0.73), h = 0.33–0.48 (0.40; n = 10); R♂ l = 0.67–0.87 (0.78), h = 0.35–0.43 (0.38; n = 9); L♂ l = 0.66–0.89 (0.75), h = 0.33–0.45 (0.38; n = 8); Rj(A-1) l = 0.53–0.64 (0.59), h = 0.30–0.34 (0.32; n = 6); Lj(A-1) l = 0.53–0.63 (0.58), h = 0.30–0.33 (0.32; n = 6); Rj(A-2) l = 0.44, h = 0.26 (n = 1); Lj(A-2) l = 0.45, h = 0.27 (n = 1).

**Remarks.** This subrectangular to subtriangular, reticulate to coarsely punctate species is very distinct due to its numerous denticles along the entire anterior margin. Juvenile individuals already display this character (e.g. Pl. 10, 49–50). This species occurs in low numbers (except AM10/7 and 30) throughout the upper part of this core. Considerable variations are observed concerning valve size within and between samples (Fig. 5) as well as in the development of its surface ornamentation ranging from reticulated (e.g. sample AM10/30; Pl. 10, Figs. 29–44) to coarsely punctated (e.g. sample AM10/23; Pl. 10, Figs. 9–16). Occasionally, the anteromarginal (both valves) and the posteroventral (only in right valves) denticulation is almost obscured by the flange (but can be clearly seen in internal view; e.g. Pl. 10, Figs. 11–12).

As already discussed by Gross *et al.* (2013), *Cyprideis pebasae* of Muñoz-Torres *et al.* (1998) and Whatley *et al.* (1998) is not conspecific with *C*. *pebasae* as established by Purper (1979), but represents a discrete taxon. Conversely, *Cyprideis lacrimata*, described as a new species by Muñoz-Torres *et al.* (1998) is synonymous with *C*. *pebasae* in its original sense (see above).

Possibly, both species names are simply inverted in the publications of Muñoz-Torres *et al.* (1998) and Whatley *et al.* (1998). In any case, this taxonomic confusion must be clarified and can not be solved using the invalid species name “*lacrimata*” for this taxon as done before (Ramos 2006, in part; Ramos *et al.* 2009; Wesselingh & Ramos 2010; ICZN article 49). A new species name for *C*. *pebasae* sensu Muñoz-Torres *et al.* (1998) and sensu Whatley *et al.* (1998) is required (ICZN article 23.3.5). Here, we name this species *Cyprideis munoztorresi* nom. nov. in honour of Fernando Muñoz-Torres. The specimens described by Muñoz-Torres *et al.* (1998) under *C*. *pebasae* form the type series of *C*. *munoztorresi* among which the male left valve (FM\UAB\F60, Pl. 4, Figs. 8–9 in Muñoz-Torres *et al.* 1998) is designated as lectotype (ICZN article 74.7).

**Occurrence** (of *C*. *pebasae* sensu Muñoz-Torres *et al.* 1998). Western Amazonia (Brazil, Colombia), latest Middle to early Late Miocene (*C*. *obliquosulcata–C*. *cyrtoma* zone; Muñoz-Torres *et al.* 2006; chronostratigraphic correlation after Wesselingh & Ramos 2010).

***Cyprideis ituiae* n. sp.**

Figs. 6j–k; Pl. 11, Figs. 1–50

**Holotype.** Right female valve, AM10-23_18 (Inv. No. MPEG-309-M; coll. Museu Paraense Emílio Goeldi, Belém; Pl. 11, Figs. 19–20, 47–49).

**Paratypes.** Additionally figured specimens (Figs. 6j–k; Pl. 11, Figs. 1–18, 21–46, 50; coll. Museu Paraense Emílio Goeldi, Belém).

**Additional material.** 315 adult and 13 juvenile specimens from samples AM10/15, 19, 22–23, 25, 27, 29–30 (Inv. No. UMJG&P 211.038; coll. Universalmuseum Joanneum, Department for Geology & Palaeontology, Graz).

**Type locality.** Borehole 1AS-10-AM at Sucuriju close to Rio Ituí (S 04°50’/W 70°22’, ~62 km SW Benjamin Constant; municipality Atalaia do Norte, state of Amazonia, Brazil).

**Type horizon.** Sample AM10/23 (= depth: 118 m, altitude: -33 m).

**Derivation of name.** Rio Ituí (river in western Amazonia), where well 1AS-10-AM has been drilled.

**Diagnosis.** A reticulated to coarsely punctated *Cyprideis*-species with strong, distally broadened and indented anteromarginal denticles and distinct, groove-like sulcus.

**Description.** Subrectangular (female) or subtrapezoidal (male) outline in lateral view. Surface reticulated to coarsely punctated; dorsomedian sulcus prominent and anteroventrally orientated; flange anteromarginally and posteroventrally thick, forming a robust rim; reticulum anteroperipherally reduced to a row of low, sub-squarish meshes behind which the surface is smooth or micro-punctate. About 7, distally expanding, indented anteromarginal denticles of which the uppermost and smallest one is cone-shaped and located just above the half of valves’ height; up to 6, well-developed posteroventral spines in right valves with the spine in the posteroventral corner being the longest one; below, already on the ventral margin, one additional, strong posteroventral spine is sometimes developed. Numerous, roundish normal pores of sieve type. Inner lamella moderately wide with numerous straight, occasionally branched marginal pore canals; avestibulate. Hinge (right valve): anterior element elongate with ~9 toothlets; short anteromedian element with 3 larger sockets; long posteromedian element consisting of a crenulated bar; posterior element with ~7 toothlets; hinge elements of left valve complementary. Central muscle scars consist of 4 adductor scars, 1 U- or V-shaped frontal and 2 mandibular scars (the upper one round–elongate; the lower, oval one located close to the ventral margin); prominent fulcral point (knob). Sexual dimorphism distinct: males are more elongated with the posterior margin more oblique. Juveniles are subtriangular in lateral view; the hinge and especially the inner lamella are weakly developed.

**Dimensions** (total range over all samples). R♀ l = 0.59–0.78 (0.66), h = 0.29–0.36 (0.33; n = 50); L♀ l = 0.62–0.76 (0.68), h = 0.31–0.40 (0.35; n = 34); R♂ l = 0.69–0.81 (0.74), h = 0.31–0.37 (0.34; n = 17); L♂ l = 0.69–0.82 (0.76), h = 0.34–0.39 (0.36; n = 22); Rj(A-1) l = 0.52–0.61 (0.57), h = 0.27–0.31 (0.29; n = 2); Lj(A-1) l = 0.60–0.61 (0.61), h = 0.31–0.32 (0.32; n = 2). See also figure 5.

**Remarks.** This species is very characteristic due to its distally widened, teeth-like anterior spines. *C*. *ituiae* is similar to *C*. *pebasae* from which it differs by the shape of the anterior spines (in *C*. *pebasae* they are pointed) and the well-developed sulcus, which forms a dorsomedian groove (*C*. *pebasae* is asulcate). While in *C*. *pebasae* posteroventral spines (right valves) are inconspicuous, *C*. *ituiae* bears longer spines with the most developed spine placed in the posteroventral-corner (occasionally an additional, long spine is observable on the ventral margin (e.g. Pl. 11, Figs. 23–24, 49). Further, the ornament of *C*. *ituiae* is reduced anteroperipherally (tiny puncta to almost smooth), behind a thick flange (e.g. Pl. 11, Figs. 45, 47).

**Occurrence.** Western Amazonia (Brazil, this study), latest Middle to early Late Miocene (*C*. *obliquosulcata–C*. *cyrtoma* zone; this study; chronostratigraphic correlation after Wesselingh & Ramos 2010).

***Cyprideis matorae* n. sp.**

Figs. 6l–m; Pl. 12, Figs. 1–14

**Table:** 

?	2011 *Cyprideis* sp. 3—Linhares *et al.*: 95, 98; Fig. 3/15.

**Holotype.** Right female valve, AM10-30_103 (Inv. No. MPEG-445-M; coll. Museu Paraense Emílio Goeldi, Belém; Pl. 12, Figs. 3–4, 7, 12–13).

**Paratypes.** Additionally figured specimens (Figs. 6l–m; Pl. 12, Figs. 1–2, 5–6, 9–10; coll. Museu Paraense Emílio Goeldi, Belém).

**Additional material.** 91 adult and 14 juvenile specimens from samples AM10/22, 30 (Inv. No. UMJG&P 211.038; coll. Universalmuseum Joanneum, Department for Geology & Palaeontology, Graz).

**Type locality.** Borehole 1AS-10-AM at Sucuriju close to Rio Ituí (S 04°50’/W 70°22’, ~62 km SW Benjamin Constant; municipality Atalaia do Norte, state of Amazonia, Brazil).

**Type horizon.** Sample AM10/30 (= depth: 141.2 m, altitude: -56.2 m).

**Derivation of name.** After “Sucuriju”, native name for a mythic giant anaconda, which was called “matora” (bull eater) by conquistadores.

**Diagnosis.** A subtriangular *Cyprideis*-species ornamented with a low, polygonal reticulum and 7–9, thick anterior spines along the entire anterior margin. Right valves with one massive posteroventral spine and additional 3–4 smaller spines above; left valves with one, robust posteroventral spine.

**Description.** Subtriangular outline in lateral view (females and males). Surface reticulated, anteroperipherally almost smooth; the dorsomedian sulcus forms a dorsomedian depression; flange anteromarginally and posteroventrally very thick, forming a robust rim and a posteroventral extension in left valves; 7–9, blunt or robust-conical anteromarginal spines along the entire anterior margin; up to 5 posteroventral spines in right valves with the spine in the posteroventral corner being the longest; one robust posteroventral spine in left valves. Scattered, roundish normal pores of sieve type. Inner lamella moderately wide with numerous straight, simple, occasionally branched marginal pore canals; avestibulate. Hinge (right valve): anterior element elongate with ~10 toothlets; short, crenulated anteromedian element; moderately long posteromedian element consisting of a crenulated bar; posterior element with ~7 toothlets; hinge elements of left valve complementary. Central muscle scars with 4 adductor scars, 1 U- or V-shaped frontal and 2 mandibular scars (the upper one roundish; the lower, oval one located close to the ventral margin); prominent fulcral point (knob). Sexual dimorphism: males are slightly more elongated with a more oblique posterior margin; the posterior proportion is wider in females (dorsal view) than in males.

**Dimensions** (total range over all samples). R♀ l = 0.73–0.79 (0.76), h = 0.39–0.41 (0.40; n = 3); L♀ l = 0.80, h = 0.43 (n = 1); R♂ l = 0.71–0.81 (0.76), h = 0.35–0.39 (0.37; n = 2); L♂ l = 0.77–0.85 (0.80), h = 0.38–0.42 (0.40; n = 3).

**Remarks.** Only few, well-preserved specimens are available from sample AM10/30 (sample AM10/22 yields more, but badly preserved material). Nevertheless, this species is well characterised by its distinct subtriangular outline, its low, polygonal reticulum and its massive posteroventral flange in left valves, armed with a strong posteroventral spine. Possibly, *Cyprideis* sp. 3 of Linhares *et al.* (2011) represents a male specimen of this species.

The most similar species are *C*. *curucae* and, especially, *C*. *ituiae*. However, the latter (Pl. 11) is subrectangular (the dorsal margin is less inclined towards the posterior); the muri of its reticulum are much thicker (tending towards a punctated surface ornament); it has a groove-like sulcus (in *C*. *matorae* the sulcus forms a dorsomedian depression); it lacks a posteroventral spine in left valves; its posteroventral flange in left valves is less prominent; characteristic distally widened spines occur not along its entire anterior margin. *C. ituiae* is smaller where it co-occurs with *C*. *matorae*.

*C*. *curucae* (Pl. 8, Figs. 10–23) has a less tapered posterior end (the posterior margin is less oblique); it lacks a posteroventral spine in left valves and the extended posteroventral flange; its inner lamella is much wider; its anteromarginal and posteroventral spines as well as the ornament are completely different compared to *C. matorae*. *C. curucae* is larger where both species co-occur.

*C*. *matorae* resembles to some degree *Cyprideis longispina* (Purper, 1979) and *C*. *graciosa* but clearly diverge in outline, ornament and development of spines (e.g. Purper 1979; Muñoz-Torres *et al.* 1998; Gross *et al.* 2013; see chapter 4.6.2.).

**Occurrence.** Western Amazonia (Brazil), latest Middle to early Late Miocene (*C*. *obliquosulcata–C*. *cyrtoma* zone; this study; possibly as *Cyprideis* sp. 3 of Linhares *et al.* 2011 in core 1AS-31-AM (depth: 172.40 m, altitude -70.40 m); assigned to *C. caraionae* zone (Linhares *et al.* 2011) but possibly younger (?*C*. *obliquosulcata* zone) due to occurrence of *C*. *cyrtoma*).

#### 4.6.4 “Ornate” species not attributed to subgroups

***Cyprideis inversa* (Purper & Pinto, 1983)**

Fig. 6n; Pl. 12, Figs. 15–19

**Table:** 

*	1983 *Sohnicythere inversa* Purper et Pinto sp. nov.—Purper & Pinto: 119–120; Pl. 3, Figs. 12–24.
	1998 *Cyprideis inversa* (Purper & Pinto, 1983)—Muñoz-Torres *et al.*: 96; Pl. 3, Figs. 4–6.
	1998 *Cyprideis inversa* (Purper & Pinto, 1983)—Whatley *et al.*: 234–235; Text-fig. 2; Pl. 1, Figs. 16–20.
	2011 *Cyprideis inversa*—Linhares *et al.*: 95, 98; Fig. 3/11.

**Material.** 60 valves; samples AM10/15, 22, 28, 30.

**Dimensions** (total range over all samples). R♀ l = 0.70, h = 0.37 (n = 1); L♀ l = 0.67, h = 0.36 (n = 1); R♂ l = 0.63, h = 0.33 (n = 1); L♂ l = 0.58, h = 0.32 (n = 1).

**Remarks.** This comparably small *Cyprideis* species is characterised by its subtriangular outline in lateral view, its punctate ornamentation with scattered tubercles (pore conuli), well-developed, densely arranged spines along the entire anterior margin, up to seven posteroventral spines (only in left valves) as well as its inverse hinge and valve overlap (Purper & Pinto 1983; Whatley *et al.* 1998). *C*. *inversa* occurs only sporadically within the core material, however, the available specimens match well with the given synonyms.

**Occurrence.** Western Amazonia (Brazil, Colombia, Peru), late Middle to early Late Miocene (*C*. *caraionae–C*. *cyrtoma* zone; Muñoz-Torres *et al.* 2006; chronostratigraphic correlation after Wesselingh & Ramos 2010).

***Cyprideis sulcosigmoidalis* (Purper, 1979)**

Fig. 6o; Pl. 12, Figs. 20–34; Pl. 13, Figs. 1–21; Pl. 14, Figs. 1–21

**Table:** 

	1977 *Cytheridea* sp.nov. A—Purper: 361; Pl. 2, Figs. 1–6.
*	1979 *Cytheridea sulcosigmoidalis* Purper, sp. nov.—Purper: 226–227; Pl. 1, Figs. 11–18.
pars	1980 *Cyprideis purperi purperi* subsp. nov.—Sheppard & Bate: 99–101; Pl. 7, Fig. 13 and probably Fig. 12. [non Text-fig. 2; Pl. 7, Figs. 1–11; Pl. 8, Figs. 1–2]
	1998 *Cyprideis sulcosigmoidalis* (Purper, 1979)—Whatley *et al.*: 236; Text-fig. 2; Pl. 3, Figs. 1–5.
	1998 *Cyprideis* sp. 4—Whatley *et al.*: 237; Pl. 3, Figs. 16–20.
	1998 *Cyprideis aulakos* sp. nov.—Muñoz-Torres *et al.*: 94; Text-fig. 2; Pl. 2, Figs. 7–11.
	1998 *Cyprideis sulcosigmoidalis* (Purper, 1979)—Muñoz-Torres *et al.*: 100; Pl. 4, Figs. 16–18.
	1998 *Cyprideis* sp. aff. *C. retrobispinosa* Purper and Pinto—Swain: 3; Pl. 6, Figs. 1–8.
	2011 *Cyprideis aulakos*—Linhares *et al.*: 95; Figs. 3/3–4.
	2011 *Cyprideis sulcosigmoidalis*—Linhares *et al.*: 95; Figs. 4/5–6.

**Material.** 1,839 valves; samples AM10/3, 6–7, 15–16, 19–31, 33, 35, 39–44.

**Dimensions** (total range over all samples). R♀ l = 0.84–1.16 (0.99), h = 0.50–0.68 (0.58; n = 186); L♀ l = 0.92–1.17 (1.04), h = 0.57–0.72 (0.63; n = 14); R♂ l = 0.98–1.13 (1.08), h = 0.51–0.66 (0.60; n = 15); L♂ l = 0.96–1.22 (1.08), h = 0.57–0.73 (0.63; n = 13).

**Remarks.** The current material complies with *C*. *sulcosigmoidalis* of Purper (1979) and Linhares *et al.* (2011) and *C*. *aulakos* of Muñoz-Torres *et al.* (1998 = *Cyprideis* sp. 4 in Whatley *et al.* 1998; Linhares *et al.* 2011). The specimen figured by Sheppard & Bate (1980) on plate 7, figure 13 (and most probably figure 12; Purper & Pinto 1985) as well as *Cyprideis* sp. aff. *C*. *retrobispinosa* in Swain (1998) actually belong to *C*. *sulcosigmoidalis*.

*C*. *sulcosigmoidalis* is a subovate–subtrapezoidal, punctate, avestibulate species with pronounced, sinuous sulcus. While the ventral margin of left valves is only slightly concave, the anteroventral margin is more curved in right valves, which cause a more pronounced ventral concavity. Puncta are randomly arranged centrally but form concentric rows along the free valve margin. The hinge (right valve) is formed by robustly denticulated anterior and posterior elements; the long, crenulated median element is composed of a short anteromedian groove and a long posteromedian bar. Marginal pore canals are numerous, simple or rarely bifurcated; normal pores are of sieve-type. Males are more elongated and have a more oblique posterior margin than females (Purper 1979; Muñoz-Torres *et al.* 1998; Whatley *et al.* 1998; note: due to its outline, the “female” holotype represents in fact a male individual). Anterior (both valves) and posterior denticles (only right valves) as illustrated by Muñoz-Torres *et al.* (1998) and Whatley *et al.* (1998) have not been mentioned in the original description by Purper (1979). Conversely, Purper (1979: 226) stated for *C*. *sulcosigmoidalis*: “it does not present spines on the anterior margin”.

By examining the original descriptions and figurations of *C*. *sulcosigmoidalis* and *C*. *aulakos*, there is virtually no clear-cut difference between both taxa (neither in outline, development of the sulcus, the inner lamella, pore canals nor in the hinge). In addition to the trait of marginal denticulation, which was later introduced by Muñoz-Torres *et al.* (1998) and Whatley *et al.* (1998), the only difference is that *C*. *aulakos* “lacks parallel punctuation peripherally and the ornament is smoother […] although some specimens are densely punctate anteriorly” (Whatley *et al.* 1998: 237; see also Muñoz-Torres *et al.* 1998: 94).

Within the present material considerable variations in ornamentation within samples (e.g. AM10/23: Pl. 13, Figs. 8, 11) and, especially, between samples are evident (e.g. AM10/7: Pl. 12, Fig. 27; AM10/3: Pl. 12, Fig. 21). Specimens from the lower part of the core (AM10/43–25) and the uppermost sample (AM10/3) have a *sulcosigmoidalis*-type ornament. In-between, transitional morphotypes occur (AM10/23–15) and only in samples AM10/7–6 smooth, *aulakos*-type valves dominate (Fig. 7). Based on this observation, the diagnostic feature “ornament” sensu Muñoz-Torres *et al.* (1998) is problematic and most likely ecologically controlled.

Anterior marginal denticles are only poorly developed in our specimens (rather present as a crenulation; frequently only seen in internal view; Fig. 7). As far as the material enables, anterior denticulations are restricted to the lower part of 1AS-10-AM (~up to AM10/27). However, referring to the original description of *C*. *sulcosigmoidalis,* in this case the degree of marginal denticulation seems to be a weak diagnostic trait. After personal observation (M.G.), it can be stated that in core 1AS-33-AM (sample depth: 290.1 and 356.3 m), *C*. *sulcosigmoidalis* with numerous anteromarginal denticles on right valves occur in the strata where *Cyprideis caraionae* was described by Purper & Pinto (1985). The ornamentation (almost smooth to coarsely punctate) varies considerably within these samples, analogous to the material of 1AS-10-AM. Conversely, *C*. *sulcosigmoidalis* from the uppermost layers (sample depth: 37.5 and 44.1 m) of core 1AS-4a-AM lacks these denticles but equals the specimens from AM10/30 in outline, ornament and, in particular, in the position of the selvage (see below). Possible evolutionary trends (e.g. progressive reduction of spines) require further investigations.

For these reasons, we suppose that ornament and marginal denticulation are not sufficient characters to delineate *C*. *aulakos* from *C*. *sulcosigmoidalis* and regard them as synonyms.

Throughout the core no significant variations of the inner lamella, pore canals, hinge or muscle scars were found. Nonetheless, *C*. *sulcosigmoidalis* varies noticeably in size in core 1AS-10-AM (e.g. large valves in AM10/30, small ones in AM10/27; Fig. 7), but this is not straightforwardly correlated with the characters discussed above and probably linked to another ecological parameter.

A further observation concerns the length/height ratio (Fig. 7): specimens of the lowermost samples (AM10/43–39) are more elongated in outline than the specimens up-section (e.g. Pl. 14, Figs. 18–21). Because of the limited and badly preserved material from this part of the core, it remains highly speculative to discuss a possible phylogenetic trend here.

Nevertheless, one—admittedly subtle—character seems to change gradually within the core section: the course of the selvage on the anterior margin of right valves (Fig. 7) and, connected with this (but less well expressed), the course of the anterodorsal outline. In specimens from samples up to AM10/29, the selvage is located proximally in the anterior and anterodorsal valve region. It shifts distally in the anterodorsal region in specimens from samples AM10/28-15, while in specimens from the uppermost samples AM10/7-3, the selvage is placed distally in both the anterior and anterodorsal valve regions. Potentially, this shift of the selvage marks a delicate evolutionary change within *C*. *sulcosigmoidalis*, which could be of further biostratigraphic importance. Additional analyses of other cores are necessary to prove or refute this claim.

However, *C*. *sulcosigmoidalis* and *C*. *aulakos* are placed in two different lineages (*C*. *sulcosigmoidalis* in the “ornate”, *C*. *aulakos* in the “smooth” lineage) in the phylogenetic scheme of Whatley *et al.* (1998) and Muñoz-Torres *et al.* (2006). Following our observations, both are not only very closely related but are synonyms. Thus, the proposed phylogeny of Amazonian *Cyprideis* needs substantial reorganisation (Fig. 3a). Furthermore, the “first appearance datum” of *C*. *aulakos* marks the lower boundary of the *C*. *aulakos* zone (Muñoz-Torres *et al.* 2006). With the rejection of this species, this zone is largely challenged (Fig. 8). It is left open to further investigations, if the occurrence of the *aulakos*-morphotype has an ecological implication and is at least of (local) ecostratigraphic value.

**Occurrence** (including the synonymous *C*. *aulakos*). Western Amazonia (Brazil, Colombia, Peru), early Middle to early Late Miocene (*C*. *aulakos–C*. *cyrtoma* zone; Muñoz-Torres *et al.* 2006; chronostratigraphic correlation after Wesselingh & Ramos 2010).

## 5 Discussion

### 5.1 Biostratigraphic implications

Muñoz-Torres *et al.* (2006) proposed an ostracod-based biozonation for the Pebas/Solimões Formation, which is—similar to mollusc zones (Wesselingh *et al.* 2006b)— tightly linked to earlier palynological zonations (Hoorn 1993, 1994b; Fig. 8). Intensive debates about definitions of pollen zones and their chronostratigraphic allocation still characterise significant uncertainties of western Amazonia’s stratigraphy (Jaramillo *et al.* 2010; Latrubesse *et al.* 2010; Silva-Caminha *et al.* 2010; Dino *et al.* 2012; Gross *et al.* 2013; compare Jaramillo *et al.* 2011 for detailed palynological zonations of the Llanos Basin, Colombia). Here, we apply the stratigraphic concept of Wesselingh & Ramos (2010; correlation of mollusc and ostracod zones; chronostratigraphy) as well as of Wesselingh *et al.* (2006b; correlation of pollen and mollusc zones; Fig. 9), being aware that considerable readjustments of biozone correlations and chronology are required.

Among the ostracod index species sensu Muñoz-Torres *et al.* (2006) found in core 1AS-10-AM, *C*. *minipunctata* has its last occurrence in sample AM10/31 (Fig. 10). As the last appearance of this species defines the top of the *C*. *minipunctata* zone (Muñoz-Torres *et al.* 2006), the productive samples below AM10/30 (>141.2 m depth) can be assigned to the *C*. *minipunctata* zone and/or to the downward succeeding *C*. *caraionae* zone. *C*. *schedogymnos* is restricted to samples AM10/40–44 in the present core. It points to the same direction, because it appears in the *C*. *caraionae* zone and vanishes at the end of the *C*. *minipunctata* zone (Muñoz-Torres *et al.* 2006). Similarly, *C*. *simplex* (as far as it is differentiated as a separate species) occurs in the *C*. *caraionae* zone. The latter zone is characterised by the first and last appearance of *C*. *caraionae*, which is, however, missing in the current materials. Unpublished palynological evaluations (M. Ebner, Tübingen) of core 1AS-10-AM recorded the pollen index taxon *Grimsdalea magnaclavata* Germeraad, Hopping & Muller, 1968 throughout the core (down to 336 m depth, sample AM10/48, altitude: -251 m). The onset of the *Grimsdalea* zone coincides with the lower boundary of the *C*. *minipunctata* zone as suggested by Wesselingh & Ramos (2010). Based on this, and supported by the absence of *C*. *caraionae*, we assign the core interval from AM10/31–44 (>141.2 to 218.1 m depth) to the *C*. *minipunctata* zone.

Up-section (AM10/30), *C*. *cyrtoma* and *C*. *curucae* (= *Cyprideis* sp. 1 and 2 in Muñoz-Torres *et al.* 2006) occur for the first time in our record. Both species have their first appearance at the base of the *C*. *obliquosulcata* zone (Muñoz-Torres *et al.* 2006). This ostracod zone is characterised by the first and last appearance of *C*. *obliquosulcata*, which has not been identified here. Accordingly, the core proportion between AM10/30 and AM10/3 can be attributed to the *C*. *obliquosulcata* and the following *C*. *cyrtoma* zone only, and a differentiation of both zones is tentative.

Muñoz-Torres *et al.* (2006) mentioned that the minute limnocytherid *Skopaeocythere tetrakanthos* Whatley, Muñoz-Torres & Van Harten, 2000 is restricted to the *C*. *obliquosulcata* zone and the presence of the foraminifer *Elphidium* is also characteristic for this interval. *S*. *tetrakanthos* has been found in AM10/22, associated with dwarfed elphidiid foraminifers (also in AM10/23; pers. observ., M.G.). Thus, the boundary between the *C*. *obliquosulcata* and *C*. *cyrtoma* zones rests questionably somewhere above AM10/22 (<116.4 m depth).

### 5.2 Remarks to the western Amazonian *Cyprideis* species flock

The appraisal of western Amazonian *Cyprideis* basically depends on two works: the initial monograph by Purper (1979) and the publication of Whatley *et al.* (1998).

Purper (1979) discovered the endemism of this fauna, leading to the erection of several new species and genera (later extended by Sheppard & Bate 1980; Purper & Pinto 1983, 1985; Purper & Ornellas 1991). These studies provided 26 formally described “*Cyprideis*” species, placed in nine different genera (seven are endemic for western Amazonia).

Conversely, Whatley *et al.* (1998; and complementarily Muñoz-Torres *et al.* 1998) applied a “broader” concept—on species and genus level alike (a discussion of the latter is beyond the scope of the present contribution). Seven earlier established species were considered as synonyms, five new species were added and nine species of Sheppard & Bate (1980), Purper & Pinto (1983, 1985), Purper & Ornellas (1991) were not treated. With the emendation of the generic diagnosis of *Cyprideis* (Whatley *et al.* 1998), all these species were transferred to that genus, hence comprising 24 (inclusively synonyms: 31) formally described Amazonian *Cyprideis* species.

Here, we encounter 20 *Cyprideis* species. Five species, placed together by Whatley *et al.* (1998), are revalidated (one additional remains unclear), two species of these authors are synonymized, and two new species are defined. Thus, 30 species now form the Miocene Amazonian *Cyprideis* inventory (Fig. 3).

Although each separate valve character can be subject to serious intraspecific variation in *Cyprideis*, detailed observations on a considerable number of valves (~16 % out of ~7,200 counted valves (ESM 1, 2) revealed sufficient traits for species delineations (discussion in 4.2.). In particular, between samples, valves’ sizes and ornaments of species repeatedly vary and are obviously environmentally controlled (a discussion of potential ecological influxes is the subject of upcoming works). Nevertheless, within samples (fossil populations), size and “basic” patterns of ornamentation of species form valuable discriminating traits. Similarly, variations in marginal denticulation of specimens between strata occur, but their principal expression (e.g. shape, position) provides a valuable diagnostic character, frequently already perceptible in juvenile stages. By application of the generic conception of Whatley *et al.* (1998), the development of the inner lamella and of marginal pore canals also offer an effective taxonomic tool for species differentiation. In addition, hinge structures (inclusively reversed hinges) contribute to species diagnoses.

The observed intraspecific plasticity (especially between samples) necessitates an extensive photographic illustration. We are aware that this work is just a further step to register *Cyprideis*’ radiation in western Amazonia. Future studies, focussing on selected species only, may render some species, as outlined here, to be species complexes or lineages.

Whatley *et al.* (1998) and Muñoz-Torres *et al.* (2006) proposed a hypothetical phylogeny for Amazonian *Cyprideis* through Miocene times, which speculatively originates from one or two ancestor(s), giving rise to a “smooth” and an “ornate” lineage. Limited by the comparably short time interval covered by well 1AS-10-AM (? <2 Ma), we do not attempt to redesign that phylogeny here. Nonetheless, solely based on comparative morphology a further characterisation of the “smooth” and “ornate” groups was possible, accompanied by a reorganisation of species relations and (sub-)groupings, respectively (see 4.3.–4.5.). These taxonomic adjustments substantially concern *Cyprideis*’ phylogeny and biozonations as well.

The current study underlines once more *Cyprideis*’ remarkable capability to produce species flocks. Western Amazonian *Cyprideis* comply with the criteria of a species flock (Lecointre *et al.* 2013; see also Schön & Martens 2004; Sturmbauer 2008):

i) endemicity: up to now not a single species has been recorded in adjacent areas;
ii) monophyly: to date this criterion is hardly verifiable, and probably Amazonian *Cyprideis* is not monophyletic in a strict sense. Perhaps Purper’s original generic subdivision reflects the situation more properly; in any case, several closely related, quite rapidly evolving species (or species complexes) are proved;
iii) speciosity: due to the present study, 30 formally described *Cyprideis* species exist in western Amazonia; additionally, several other species are recorded in the literature, although left in open nomenclature until now. This strongly hints to a much higher, still unrecorded species richness within this vast and little explored region;
iv) ecological diversity: this criterion remains difficult to attest due to limited research and the fact of dealing with extinct taxa; based on rare sedimentologic cross-references, ecological diversity within a highly structured wetland is possible (Gross *et al.* 2013); the current results demonstrate the sympatric occurrence of up to 12 *Cyprideis* species, which may indicate adaptations to different microhabitats;
v) habitat dominance: regularly, *Cyprideis* holds more than 90 % in western Amazonian ostracod assemblages during the Early and Middle Miocene (Muñoz-Torres *et al.* 1998; Whatley *et al.* 1998; and this study), however, it decreases to c. 36 % close to its disappearance in Late Miocene times (Ramos 2006; Gross *et al.* 2013).

## 6 Conclusions

The micropalaeontologic investigation of well 1AS-10-AM (Solimões Basin; Brazil) permits a review of about 2/3 of hitherto described Miocene *Cyprideis* species from western Amazonia. More than 7,000 valves (out of estimated ~12,000) were counted, of which >1,000 were subject to detailed examination (light and SEM photography; basic morphometrics). This allows for conclusions on the taxonomic value of observable valve characters as well as for a demonstration of intraspecific variability (as far as possible including sexual and ontogenetic polymorphism).

Based on our observations, we refine existing *Cyprideis* species definitions (5 species are revalidated, 2 synonymised, 2 renamed and 2 defined) as well as earlier proposed species (sub-)groupings. Due to the occurrence of some ostracod index species, the core comprises sediments of the *C*. *minipunctata* to *C*. *cyrtoma* ostracod zones, corresponding to the *Grimsdalea* pollen zone and a late Middle to early Late Miocene age.

We regard the current study as a base for upcoming, more advanced analyses (e.g. geometric morphometric approaches or geochemical analyses) as well as a further step in illuminating the amazing *Cyprideis* species flock of western Amazonia’s geological past.

## Electronic supplementary material

ESM1 Dataset of the occurrences of *Cyprideis* species in core 1AS-10-AM.

ESM2 Dataset of measured and photographed specimens.

## Figures and Tables

**Plate 1:**
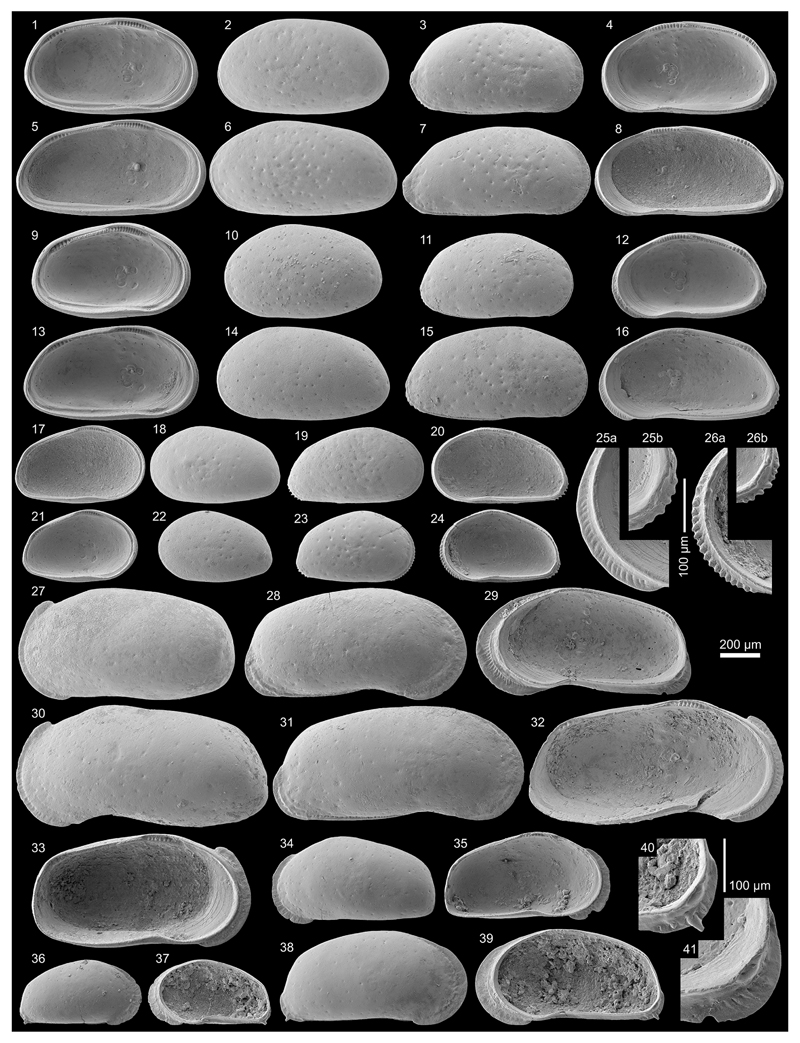
*Cyprideis amazonica* Purper, 1979 (Figs. 1–26). 1–2: L♀, i-e (0.88/0.49; AM10-7_85); 3–4: R♀, e-i (0.87/0.46; AM10-7_18); 5–6: L♂, i-e (0.96/0.49; AM10-7_86); 7–8: R♂, e-i (0.94/0.44; AM10-7_22); 9–10: L♀, i-e (0.78/0.46; AM10-39_21); 11–12: R♀, e-i (0.79/0.43; AM10-39_26); 13–14: L♂, i-e (0.89/0.48; AM10-39_18); 15: R♂, e (0.91/0.46; AM10-39_23); 16: R♂, i (0.91/0.47; AM10-39_38); 17–18: Lj, i-e (0.64/0.38; AM10-7_34); 19–20: Rj, e-i (0.69/0.38; AM10-7_66); 21–22: Lj, i-e (0.56/0.35; AM10-39_31); 23–24: Rj, e-i (0.59/0.35; AM10-39_34); 25: = details of Fig. 16; 25a = anterior and 25b posteroventral part; 26: = details of Fig. 24; 26a = anterior and 26b posteroventral part *Cyprideis machadoi* (Purper, 1979) (Figs. 27–41) 27: L♀, e (1.08/0.55; AM10-23_65) 28: R♀, e (1.11/0.55; AM10-23_71) 29: R♀, i (1.11/0.54; AM10-23_73) 30: L♂, e (1.26/0.61; AM10-23_64) 31: R♂, e (1.26/0.57; AM10-23_70) 32: = Fig. 30, i 33: = Fig. 27, i 34–35: Lj, e-i (0.81/0.43; AM10-23_67) 36–37: Rj, e-i (0.60/0.32; AM10-23_57) 38–39: Rj, e-i (0.90/0.45; AM10-23_33) 40: = detail of Fig. 39; posteroventral part 41: = detail of Fig. 29; posteroventral part

**Plate 2:**
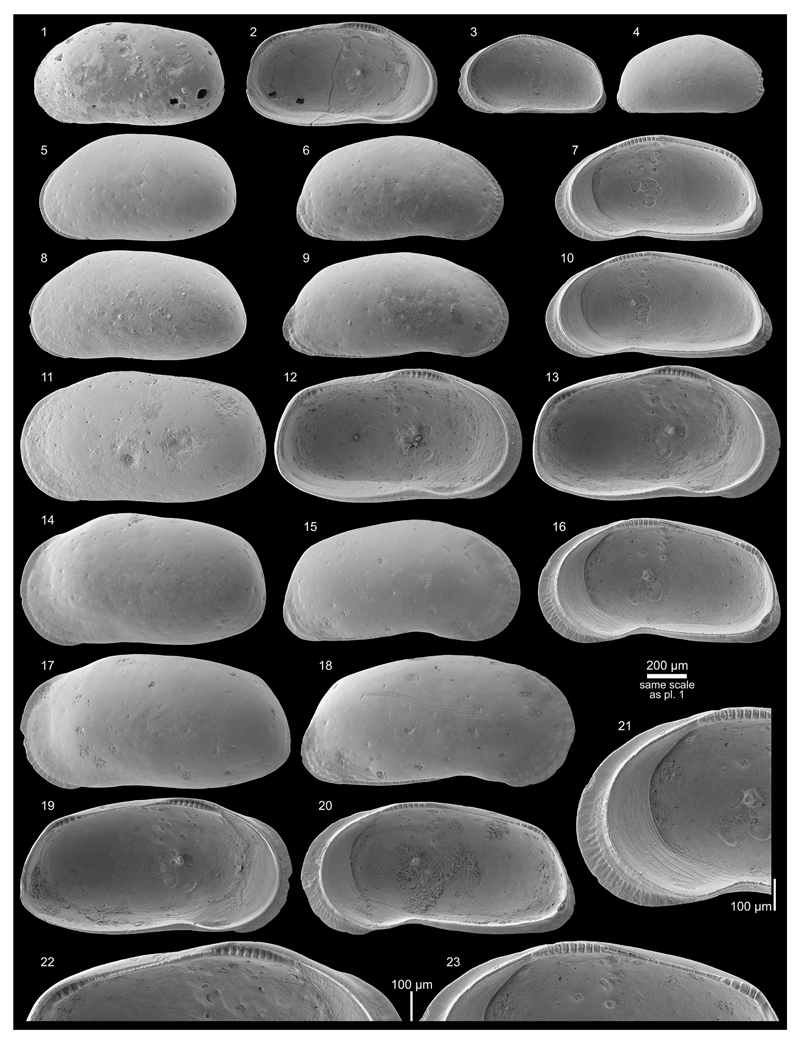
*Cyprideis machadoi* (Purper, 1979) Figs. 1–23. 1–2: L♀, e-i (0.95/0.51; AM10-25_21); 3–4: Rj, i-e (0.74/0.39; AM10-27_39); 5: L♀, e (0.99/0.54; AM10-27_32); 6-7: R♀, e-i (1.03/0.52; AM10-27_36); 8: L♂, e (1.10/0.55; AM10-27_33); 9–10: R♂, e-i (1.13/0.53; AM10-27_37); 11–12: R♀, e-i (1.25/0.67; AM10-29_15); 13–14: L♀, i-e (1.25/0.67; AM10-30_95); 15–16: R♀, e-i (1.20/0.63; AM10-30_33); 17: L♂, e (1.38/0.68; AM10-30_94); 18: R♂, e (1.39/0.66; AM10-30_96); 19: = Fig. 17, i; 20: = Fig. 18, i; 21: = detail of Fig. 16; anterior part; 22: = detail of Fig. 13; hinge; 23: = detail of Fig. 16; hinge.

**Plate 3:**
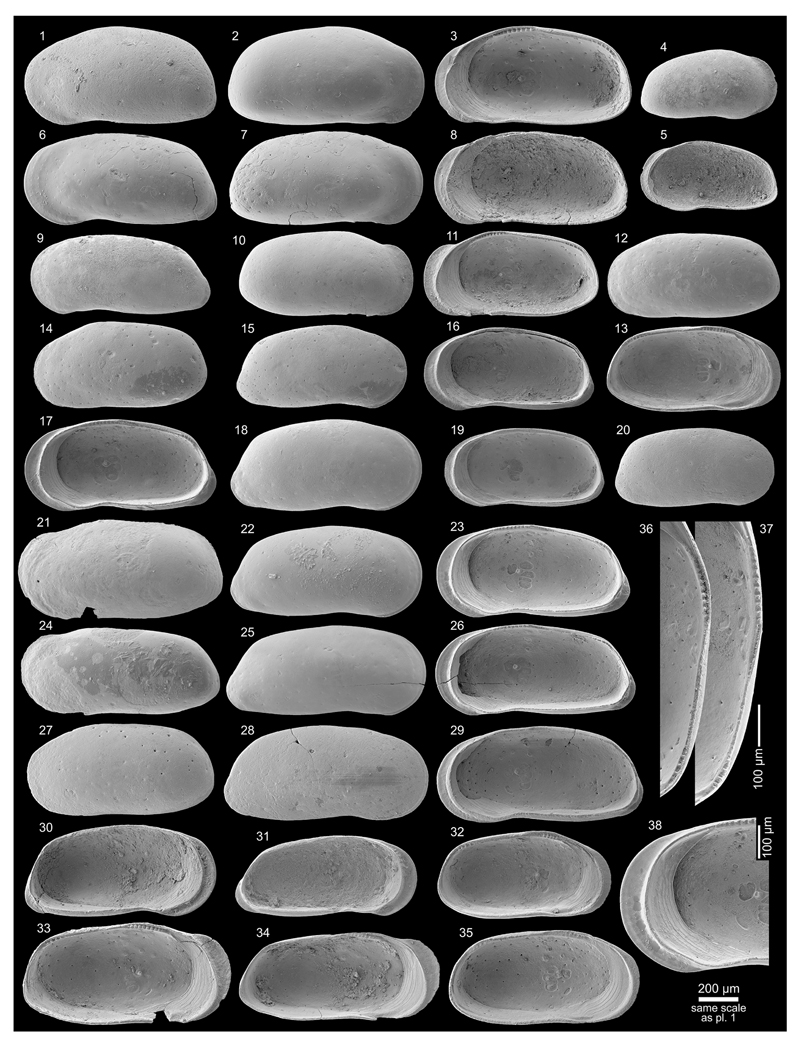
*Cyprideis kotzianae* (Purper & Ornellas, 1991) Figs. 1–38 1: L♀, e (0.93/0.48; AM10-3_01); inverse form 2–3: R♀, e-i (0.97/0.49; AM10-3_03); inverse form 4–5: Rj, e-i (0.68/0.34; AM10-23_75); inverse form 6: L♂, e (0.96/0.46; AM10-3_02); inverse form 7–8: R♂, e-i (0.96/0.45; AM10-3_05); inverse form 9: L♂, e (0.91/0.40; AM10-23_106); inverse form 10–11: R♀, e-i (0.87/0.42; AM10-23_74); inverse form 12–13: L♀, e-i (1AS-32-AM; 0.87/0.41; AM32-5_26) 14: L♀, e (0.87/0.43; AM10-25_33) 15–16: R♀?, e-i (0.86/0.41; AM10-25_34) 17–18: R♀, i-e (0.97/0.45; AM10-29_07) 19–20: R♀, i-e (1AS-32-AM; 0.80/0.40; AM32-2_26) 21: L♀, e (1.00/0.49; AM10-30_34) 22–23: R♀, e-i (0.95/0.44; AM10-30_35) 24: L♂, e (1.01/0.43; AM10-30_92) 25–26: R♂, e-i (0.99/0.44; AM10-30_31) 27: L♀, e (0.96/0.47; AM10-40_05) 28–29: R♀, e-i (1.04/0.48; AM10-40_06) 30: = Fig. 6, i 31: = Fig. 9, i 32: = Fig. 14, i 33: = Fig. 21, i 34: = Fig. 24, i 35: = Fig. 27, i 36: = detail of Fig. 11; inverse hinge 37: = detail of Fig. 23; hinge 38: = detail of Fig. 23; anterior part

**Plate 4:**
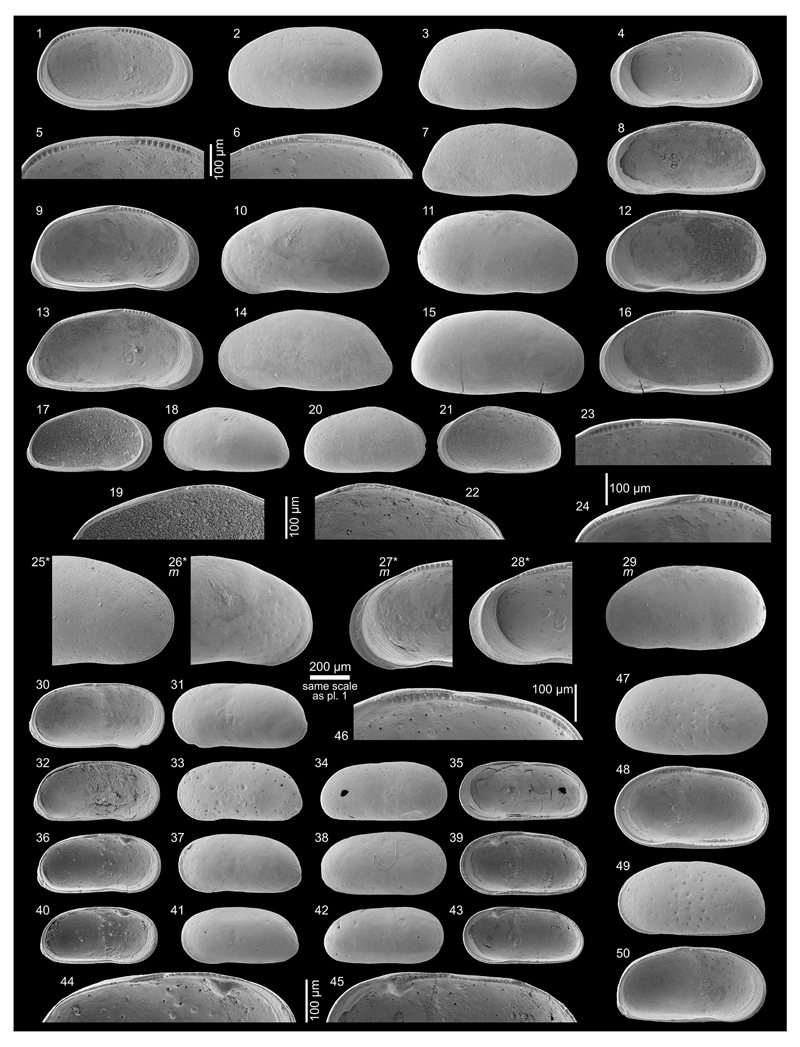
*Cyprideis olivencai* (Purper, 1979) Figs. 1–8, 25, 28 1-2: L♀, i-e (0.77/0.41; AM10-7_27) 3: R♀, e (0.78/0.40; AM10-7_24) 4: R♀, i (0.77/0.38; AM10-7_82) 5: = detail of Fig. 1; hinge 6: = detail of Fig. 4; hinge 7-8: R♂, e-i (AM10-7_83) 25: = detail of Fig. 3; anterior part (*not to scale) 28: = detail of Fig. 4; anterior part (*not to scale) *Cyprideis kroemmelbeini* (Purper, 1979) Figs. 9–24, 26–27, 29 (all: “inverse” specimens) 9–10: L♀, i-e (0.85/0.43; AM10-7_69) 11–12: R♀, e-i (0.81/0.43; AM10-7_76) 13–14: L♂, i-e (0.90/0.42; AM10-7_78) 15–16: R♂, e-i (0.88/0.43; AM10-7_74) 17–18: Lj, i-e (0.62/0.32; AM10-7_40) 19: = detail of Fig. 17; hinge 20–21: Rj, e-i (0.64/0.34; AM10-7_87) 22: = detail of Fig. 21; hinge 23: = detail of Fig. 16; hinge 24: = detail of Fig. 9; hinge 26–27: = details of Figs. 10 and 9; anterior part (mirrored, *not to scale) 29: = Fig. 11 (mirrored) *Cyprideis paralela* (Purper, 1979) Figs. 30–45 (all: “inverse” specimens) 30–31: L♀, i-e (0.67/0.32; AM10-15_03) 32–33: L♀, i-e (0.63/0.30; AM10-25_39) 34–35: R♂, e-i (0.65/0.30; AM10-25_42) 36–37: L♀?, i-e (0.61/0.29; AM10-30_41) 38–39: R♀, e-i (0.62/0.31; AM10-30_44) 40–41: L♂?, i-e (0.58/0.28; AM10-30_42) 42–43: R♂, e-i (0.59/0.28; AM10-30_43) 44: = detail of Fig. 36; hinge 45: = detail of Fig. 43; hinge *Cyprideis simplex* (Sheppard & Bate, 1980) Figs. 46–50 (all: “inverse” specimens) 46: = detail of Fig. 48; hinge 47–48: R♀, e-i (0.76/0.40, AM10-39_03) 49-50: L♀?, e-i (0.77/0.39; AM10-39_01)

**Plate 6:**
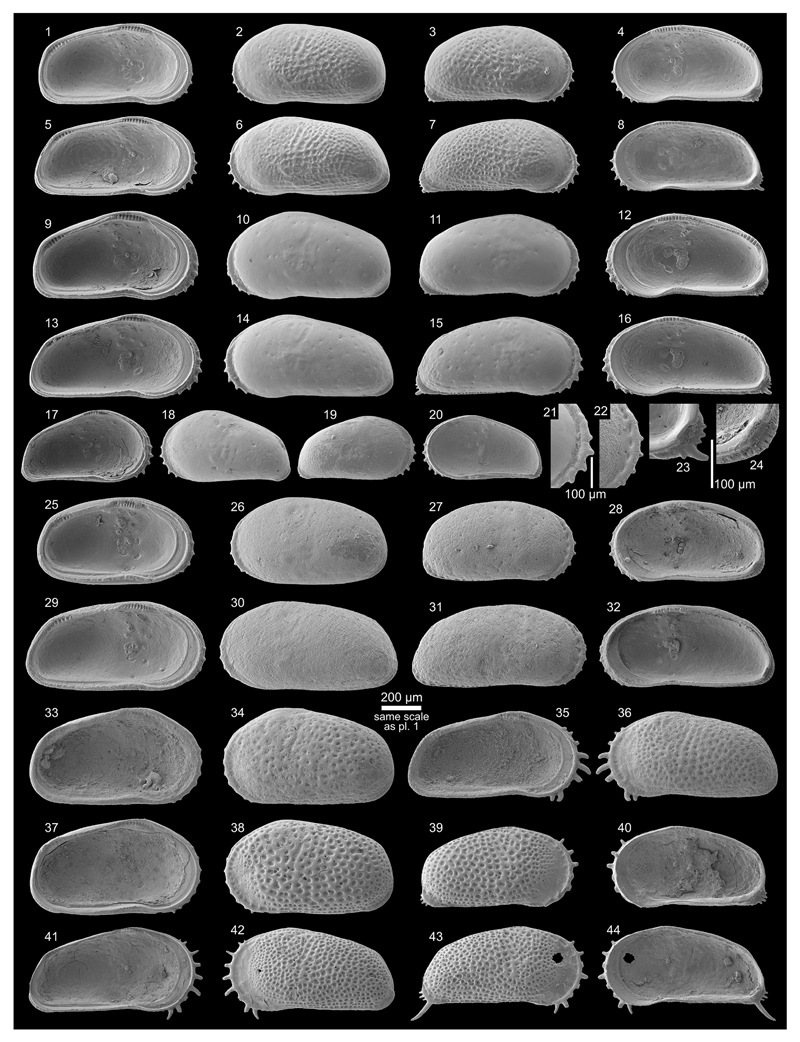
*Cyprideis cyrtoma* Muñoz-Torres, Whatley & Van Harten, 1998 Figs. 1–21, 23 1–2: L♀, i-e (0.79/0.41; AM10-27_10) 3–4: R♀, e-i (0.74/0.38; AM10-27_16) 5–6: L♂, i-e (0.81/0.39; AM10-27_09) 7: R♂, e (0.79/0.37; AM10-27_13) 8: R♂, i (0.74/0.35; AM10-27_67) 9–10: L♀, i-e (0.81/0.43; AM10-30_46) 11–12: R♀, e-i (0.80/0.41; AM10-30_50) 13–14: L♂, i-e (0.85/0.42; AM10-30_47) 15–16: R♂, e-i (0.83/0.39; AM10-30_52) 17–18: Lj, i-e (0.65/0.35; AM10-30_48) 19–20: Rj, e-i (0.58/0.32; AM10-30_109) 21: = detail of Fig. 15; anterior part 23: = detail of Fig. 8; posteroventral part *Cyprideis schedogymnos* Muñoz-Torres, Whatley & Van Harten, 1998 Figs. 22, 24–32 22: = detail of Fig. 27; anterior part 24: = detail of Fig. 28; posteroventral part 25–26: L♀, i-e (0.78/0.43; AM10-43_04) 27–28: R♀, e-i (0.76/0.39; AM10-43_09) 29–30: L♂, i-e (0.88/0.44; AM10-43_03) 31–32: R♂, e-i (0.87/0.42; AM10-43_08) *Cyprideis* aff. *graciosa* (Purper, 1979) Figs. 33–44 33–34: L♀, i-e (0.86/0.48; AM10-22_20) 35–36: L♂, i-e (0.85/0.43; AM10-22_21) 37–38: L♀, i-e (0.85/0.47; AM10-25_56) 39–40: R♀, e-i (0.76/0.40; AM10-25_52) 41–42: L♂, i-e (0.83/0.42; AM10-25_55) 43–44: R♂, e-i (0.80/0.39; AM10-25_50)

**Plate 10:**
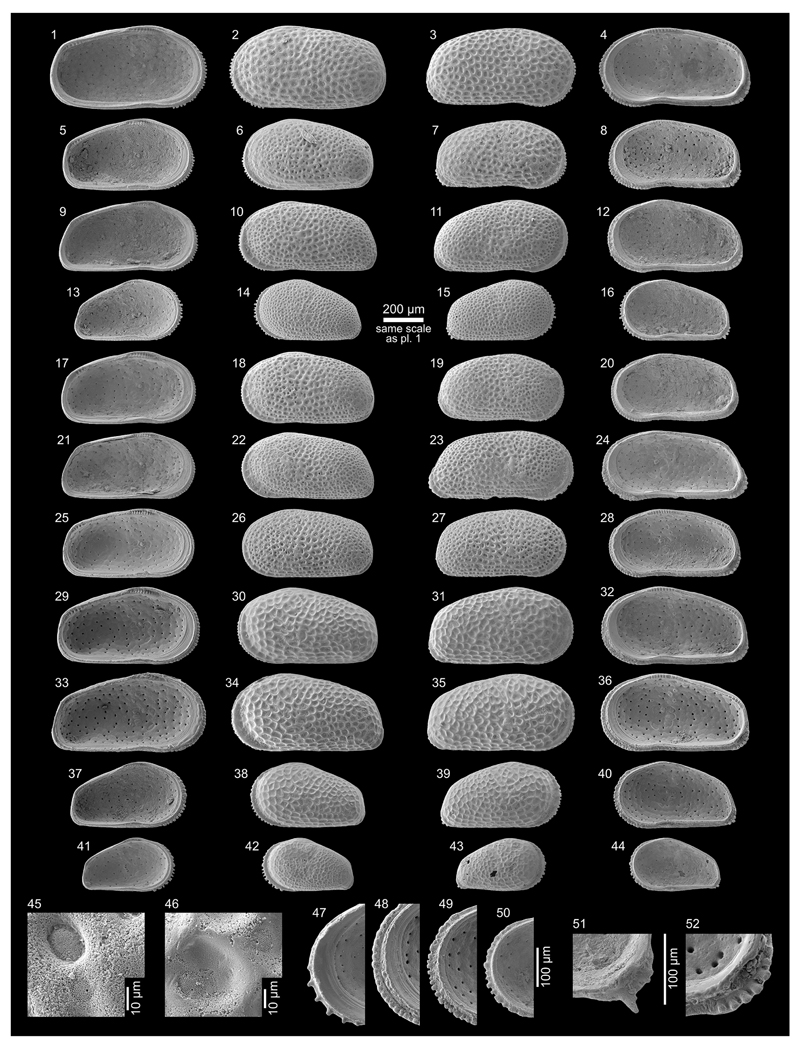
*Cyprideis munoztorresi* nom. nov. Figs. 1–44, 46, 48–50, 52 1–2: L♀, i-e (0.77/0.42; AM10-15_34) 3–4: R♀, e-i (0.74/0.39; AM10-15_33) 5–6: L♀, i-e (0.65/0.35; AM10-23_01) 7–8: R♀, e-i (0.65/0.34; AM10-23_04) 9–10: L♂, i-e (0.69/0.35; AM10-23_94) 11–12: R♂, e-i (0.67/0.36; AM10-23_95) 13–14: Lj, i-e (0.53/0.30; AM10-23_03) 15–16: Rj, e-i (0.53/0.30; AM10-23_96) 17–18: L♀, i-e (0.66/0.36; AM10-25_46) 19–20: R♀, e-i (0.63/0.34; AM10-25_48) 21–22: L♂, i-e (0.66/0.33; AM10-25_47) 23–24: R♂, e-i (0.71/0.35; AM10-25_49) 25–26: L♀, i-e (0.64/0.33; AM10-27_01) 27–28: R♀, e-i (0.65/0.33; AM10-27_02) 29–30: L♀, i-e (0.69/0.37; AM10-30_13) 31–32: R♀, e-i (0.72/0.39; AM10-30_108) 33–34: L♂, i-e (0.75/0.38; AM10-30_12) 35–36: R♀, e-i (0.72/0.38; AM10-30_17) 37–38: Lj, i-e (0.56/0.32; AM10-30_15) 39–40: Rj, e-i (0.60/0.32; AM10-30_18) 41–42: Lj, i-e (0.45/0.27; AM10-30_111) 43–44: Rj, e-i (0.44/0.26; AM10-30_112) 46: L♂, e (0.78/0.38; AM10-30_10); sieve pores 48: = detail of Fig. 36; anterior margin 49: = detail of Fig. 40; anterior margin 50: = detail of Fig. 44; anterior margin 52: = detail of Fig. 36; posteroventral part *Cyprideis pebasae* (Purper, 1979) Figs. 45, 47, 51 45: = detail of Fig. 14 on pl. 9; sieve pores 47: = detail of Fig. 15 on pl. 9; anterior margin 51: = detail of Fig. 12 on pl. 9; posteroventral part

**Plate 11:**
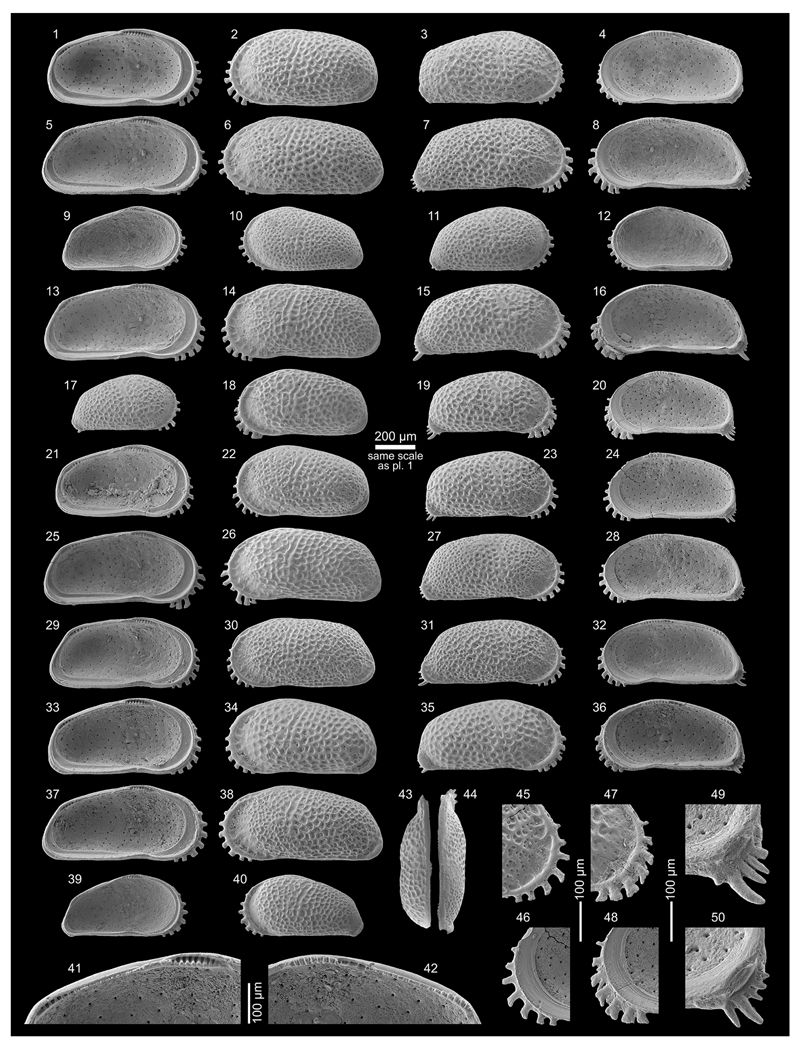
*Cyprideis ituiae* **n. sp.** Figs. 1–50 1–2: L♀, i-e (0.76/0.37; AM10-15_15) 3–4: R♀, e-i (0.72/0.36; AM10-15_20) 5–6: L♂, i-e (0.82/0.39; AM10-15_14) 7–8: R♂, e-i (0.80/0.37; AM10-15_18) 9–10: Lj, i-e (0.61/0.32; AM10-15_17) 11–12: Rj, e-i (0.61/0.31; AM10-15_21) 13–14: L♂, i-e (0.77/0.37; AM10-19_07) 15–16: R♂, e-i (0.74/0.35; AM10-19_09) 17: Rj, e (0.52/0.27; AM10-23_43) 18: L♀, e (0.65/0.33; AM10-23_107) 19–20: R♀, e-i (0.62/0.30; AM10-23_18) 21–22: L♀, i-e (0.66/0.35; AM10-23_08) 23–24: R♀, e-i (0.63/0.32; AM10-23_14) 25–26: L♂, i-e (0.76/0.36; AM10-23_100) 27–28: R♂, e-i (0.69/0.32; AM10-23_17) 29–30: L♂, i-e (0.72/0.34; AM10-27_20) 31–32: R♂, e-i (0.70/0.31; AM10-27_19) 33–34: L♀, i-e (0.73/0.37; AM10-30_56) 35–36: R♀, e-i (0.78/0.34; AM10-30_58) 37–38: L♂, i-e (0.78/0.37; AM10-30_55) 39–40: Lj, i-e (0.60/0.31; AM10-30_106) 41: = detail of Fig. 33; hinge elements 42: = detail of Fig. 36; hinge elements 43: = Fig. 18, d 44: = Fig. 27, d 45: = detail of Fig. 23; anterior part 46: = detail of Fig. 24; anterior part 47: = detail of Fig. 19; anterior part 48: = detail of Fig. 20; anterior part 49: = detail of Fig. 20; posteroventral part 50: = detail of Fig. 24; posteroventral part

**Plate 12:**
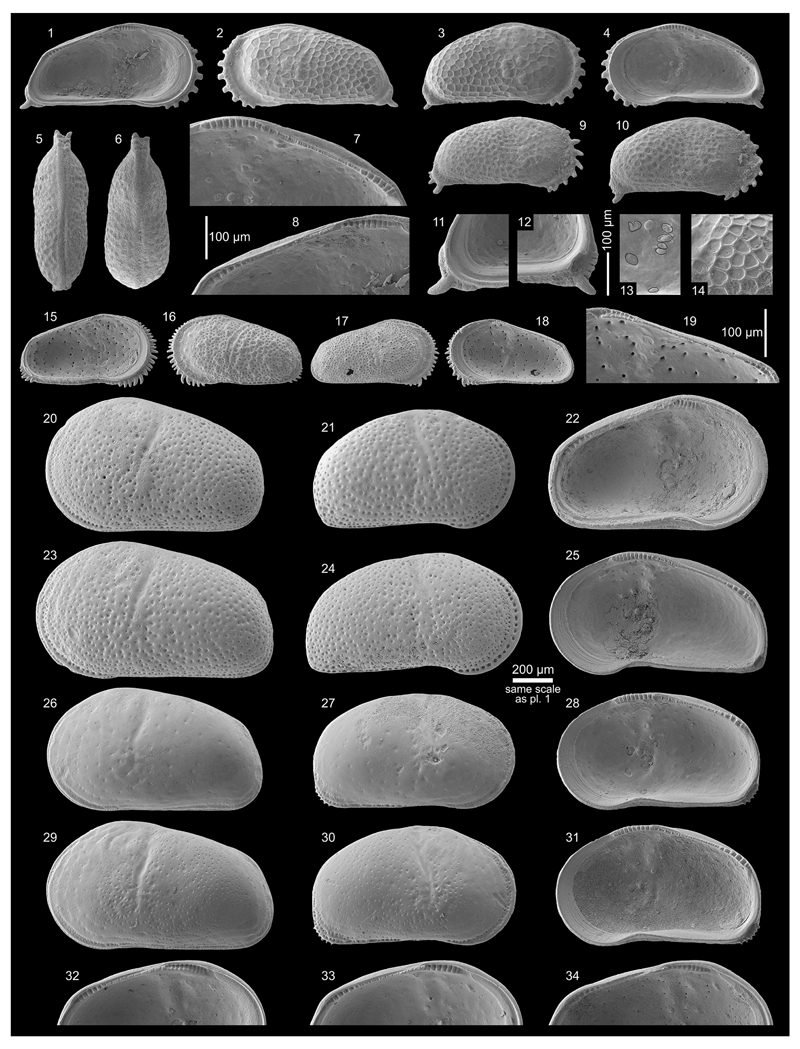
*Cyprideis matorae* **n. sp.** Figs. 1–14 1–2: L♂, i-e (0.85/0.42; AM10-30_104) 3–4: R♀, e-i (0.79/0.41; AM10-30_103) 5: C♂, d (0.78/0.39; AM10-22_28) 6: C♀, d (0.75/0.41; AM10-22_27) 7: = detail of Fig. 4; hinge 8: = detail of Fig. 1; hinge 9: R♂, e (0.71/0.35; AM10-22_24) 10: R♀, e (0.73/0.39; AM10-22_23) 11: = detail of Fig. 1; posteroventral part 12: = detail of Fig. 4; posteroventral part 13: = detail of Fig. 4; central muscle scars (retraced) 14: = detail of Fig. 2; ornamentation *Cyprideis inversa* (Purper & Pinto, 1983) Figs. 15–19 15–16: L♀, i-e (0.67/0.36; AM10-30_07) 17–18: R♂, e-i (0.63/0.33; AM10-30_09) 19: = detail of Fig. 18; hinge *Cyprideis sulcosigmoidalis* (Purper, 1979) Figs. 20–34 20: L♀, e (1.00/0.65; AM10-3_08) 21: R♀, e (1.02/0.60; AM10-3_83) 22: = Fig. 20, i 23: L♂, e (1.10/0.66; AM10-3_06) 24–25: R♂, e-i (1.10/0.51; AM10-3_09) 26: L♀, e (1.10/0.61; AM10-7_01) 27–28: R♀, e-i (1.00/0.57; AM10-7_09) 29: L♂, e (1.10/0.64; AM10-7_02) 30–31: R♀, e-i (1.00/0.59; AM10-7_11) 32: L♀, i (1.00/0.60; AM10-7_04); dorsal proportion 33: = Fig. 26, i; dorsal proportion 34: = Fig. 29, i; dorsal proportion

**Plate 13:**
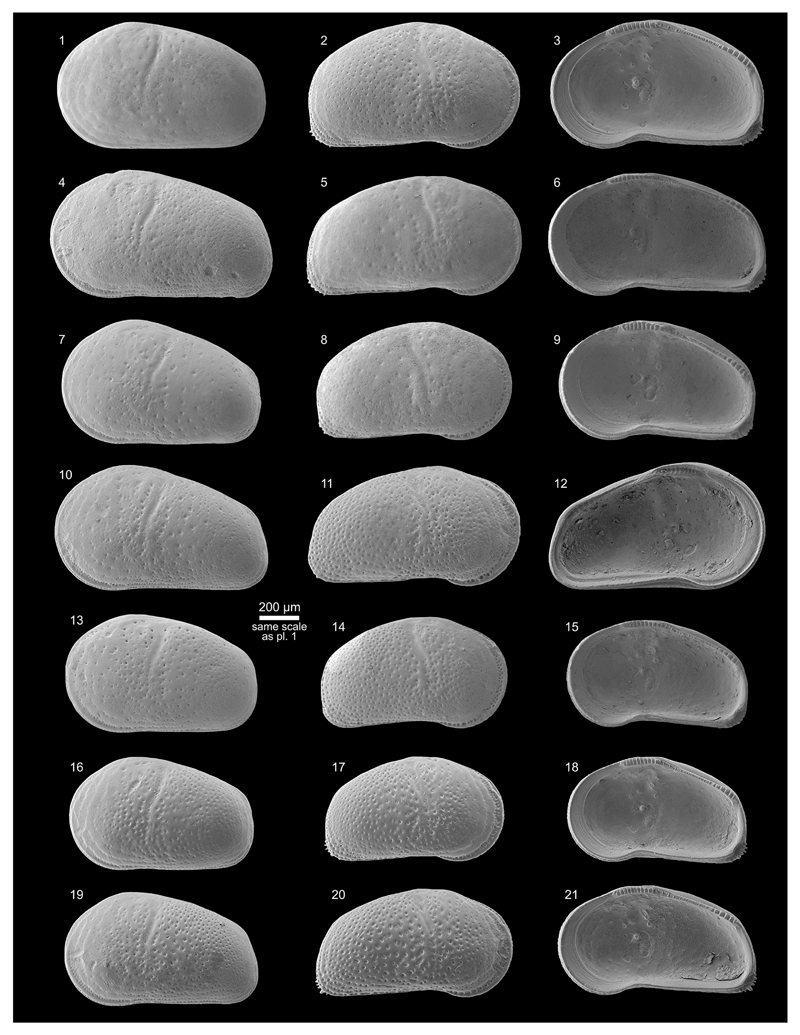
*Cyprideis sulcosigmoidalis* (Purper, 1979) Figs. 1–21. 1: L♀, e (1.10/0.64; AM10-15_10); 2–3: R♀, e-i (1.06/0.64; AM10-15_12); 4: L♂, e (1.12/0.64; AM10-15_09); 5–6: R♂, e-i (1.10/0.61; AM10-15_11); 7: L♀, e (0.99/0.62; AM10-23_77); 8–9: R♀, e-i (0.98/0.58; AM10-23_82); 10: L♂, e (1.07/0.62; AM10-23_78); 11: R♂, e (1.05/0.58; AM10-23_80); 12: = Fig. 10, i; 13: L♀, e (0.97/0.60; AM10-25_57); 14: R♀, e (0.93/0.56; AM10-25_01); 15: R♀, i (0.89/0.53; AM10-25_05); 16: L♀, e (0.92/0.57; AM10-27_42); 17–18: R♀, e-i (0.89/0.52; AM10-27_46); 19: L♂, e (0.96/0.57; AM10-27_43); 20–21: R♂, e-i (0.98/0.54; AM10-27_44).

**Plate 14:**
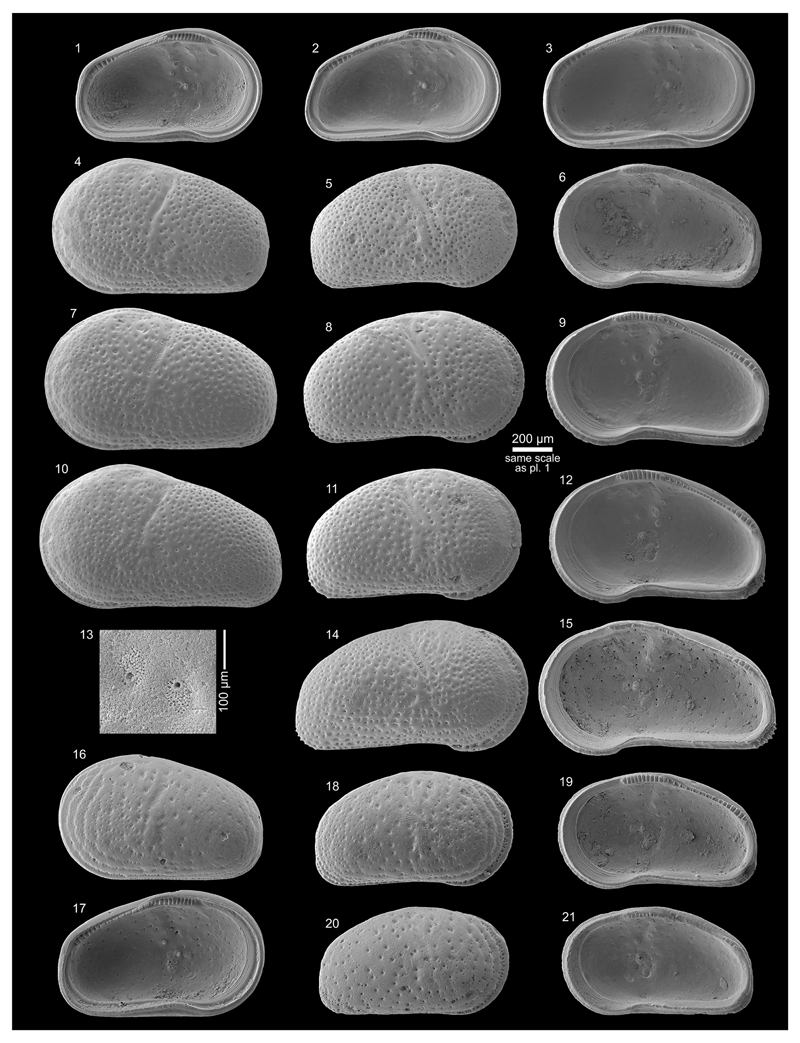
*Cyprideis sulcosigmoidalis* (Purper, 1979) Figs. 1–21. 1: L♀, i (= Fig. 16, pl. 13); 2: L♂, i (= Fig. 19, pl. 13); 3: L♀, i (= Fig. 1, pl. 13); 4: L♀, e (1.09/0.69; AM10-29_19); 5–6: R♀, e-i (1.05/0.62; AM10-29_03); 7: L♀, e (1.17/0.72; AM10-30_124); 8–9: R♀, e-i (1.12/0.65; AM10-30_77); 10: L♂, e (1.22/0.73; AM10-30_123); 11–12: R♀, e-i (1.09/0.65; AM10-30_76); 13: = detail of Fig. 10; sieve pores; 14–15: R♂, e-i (1.10/0.66; AM10-30_21); 16–17: L♀, e-i (1.00/0.62; AM10-42_02); 18–19: R♀, e-i (1.02/0.59; AM10-42_04); 20–21: R♀, e-i (0.94/0.53; AM10-43_07)
